# Synergistic efficacy of the EGFR inhibitor osimertinib with the XPO1 inhibitor selinexor in T-cell acute lymphoblastic leukemia

**DOI:** 10.1186/s40364-026-01031-w

**Published:** 2026-10-28

**Authors:** Rushuang Xu, Xiangtao Huang, Jia Liu, Yingying Shang, Na Zhang, Jiegang Xu, Shidi Cheng, Shuangnian Xu, Xi Li

**Affiliations:** 1 Department of Hematology, Daping Hospital (Army Medical Center of PLA), Army Medical University (Third Military Medical University), Chongqing, China; 2 Chongqing Key Laboratory of Hematology and Microenvironment, Chongqing, China; 3 Center for Hematology, Southwest Hospital, Army Medical University (Third Military Medical University), Chongqing, China

**Keywords:** T-cell acute lymphoblastic leukemia, Nucleolar stress, Ribosome biogenesis, c-MYC, Osimertinib, Selinexor, Biomarker

## Abstract

**Background:**

T-cell acute lymphoblastic leukemia (T-ALL) is an aggressive malignancy with poor prognosis, especially in relapsed/refractory patients. Aberrant PI3K/AKT/mTOR and NOTCH1 signaling converge on c-MYC to drive proliferation and ribosome biogenesis, but single-agent inhibitors targeting these pathways have shown limited clinical efficacy.

**Methods:**

An FDA-approved drug library screen identified osimertinib as a candidate anti-T-ALL agent. Its efficacy and mechanism were evaluated in four T-ALL cell lines (MOLT-4, Jurkat, CCRF/CEM, and MOLT-3) with different *TP53* statuses and in primary T-ALL blasts. Combination studies with selinexor, an exportin 1 (XPO1) inhibitor, were performed. Multi-omics, biochemical, and functional assays were used, and efficacy and toxicity were assessed in T-ALL xenograft models.

**Results:**

Osimertinib suppressed leukemic progression in vivo by promoting proteasome-dependent degradation of c-MYC through a non-canonical, EGFR-independent pathway that cannot be fully explained by inhibition of PI3K/AKT/mTOR or NOTCH1 signaling alone. This c-MYC suppression impaired ribosome biogenesis, and combining osimertinib with selinexor further disrupted this process. The combination increased apoptosis at low concentrations (0.5-2 µM osimertinib plus 20 nM selinexor), with clear synergy at 2 µM in all four T-ALL cell lines. Mechanistically, the combination caused nuclear retention of RPS6, nuclear accumulation of RPL5/RPL11, nucleolar stress (fibrillarin dispersal), decreased MDM2 protein levels, p53 stabilization, BAX activation (6A7), cytochrome c release, c-MYC depletion, and translational suppression. In vivo, the combination improved survival and reduced leukemic burden with minimal toxicity to normal cells at low doses. Potential biomarkers include c-MYC dependency and *TP53* status as candidate stratification factors, and treatment-induced c-MYC depletion and RPL5/RPL11 nuclear accumulation as candidate pharmacodynamic biomarkers.

**Conclusions:**

Osimertinib plus selinexor depletes c-MYC, induces nucleolar stress, and suppresses translation, which together drive T-ALL cell apoptosis. This mechanism-driven combination shows preclinical efficacy with limited toxicity in the normal-cell and xenograft models tested. Further studies incorporating pharmacokinetic monitoring, PDX models, and prospective validation of candidate biomarkers such as c-MYC dependency, *TP53* status, and RPL5/RPL11 nuclear accumulation are warranted before clinical translation.

**Supplementary Information:**

The online version contains supplementary material available at https://doi.org/10.1186/s40364-026-01031-w.

## Key points

Osimertinib synergizes with selinexor to induce nucleolar stress, translational suppression, and c-MYC depletion, which cooperatively drive T-ALL cell apoptosis.

The combination significantly prolongs survival in T-ALL xenograft models with minimal toxicity to normal cells, supporting further preclinical evaluation.

**Supplementary Information:**

The online version contains supplementary material available at https://doi.org/10.1186/s40364-026-01031-w.

## Introduction

T-cell acute lymphoblastic leukemia (T-ALL) is an aggressive hematologic malignancy with poor outcomes, especially in adults and in patients with relapsed or refractory disease [1, 2]. Despite intensive multi-agent chemotherapy, outcomes remain unsatisfactory, highlighting the need for novel therapeutic strategies [2]. The pathogenesis of T-ALL is driven by aberrant activation of oncogenic pathways, most frequently NOTCH1 and PI3K/AKT/mTOR, which converge on the master regulator c-MYC to promote proliferation, survival, and metabolic reprogramming [3, 4]. However, clinical translation of single-agent inhibitors targeting these pathways, such as γ-secretase inhibitors and PI3K/AKT/mTOR inhibitors, has been hampered by suboptimal efficacy and dose-limiting toxicities [1, 4, 5].

Ribosome biogenesis is essential for highly proliferative cancers and integrates oncogenic signaling with cellular stress responses [6–8]. Disruption of ribosome assembly triggers nucleolar stress, leading to sequestration of the E3 ubiquitin ligase MDM2 by free ribosomal proteins such as RPL5 and RPL11, with consequent stabilization and activation of p53 [9–11]. Conversely, hyperactivation of c-MYC drives elevated ribosome synthesis, creating a selective dependency on this anabolic pathway in malignant cells [7, 12, 13]. Thus, targeting ribosome biogenesis represents a promising therapeutic strategy in hematologic malignancies. However, the optimal strategy to exploit this vulnerability in T-ALL remains unclear, and biomarkers to identify patients most likely to benefit are lacking.

Through a repurposing screen of an FDA-approved drug library comprising 3,067 compounds, we identified osimertinib, a third-generation epidermal growth factor receptor (EGFR) tyrosine kinase inhibitor [14, 15], as a potent anti-leukemia agent against T-ALL cells. Although T-ALL cells express low levels of EGFR, osimertinib exhibited marked anti-leukemic activity and reduced c-MYC protein levels. Mechanistically, we found that osimertinib promotes proteasome-dependent degradation of c-MYC through a non-canonical, EGFR-independent pathway that cannot be fully explained by inhibition of PI3K/AKT/mTOR or NOTCH1 signaling alone. Given that c-MYC is a key driver of ribosome biogenesis, we hypothesized that osimertinib-mediated c-MYC suppression would impair this anabolic process.

Selinexor, a selective inhibitor of exportin 1 (XPO1), blocks nuclear export of multiple tumor suppressor proteins and ribosomal subunits, leading to their nuclear retention and apoptosis of cancer cells [16–18]. It is FDA-approved for multiple myeloma and diffuse large B-cell lymphoma, but exhibits limited single-agent activity in T-ALL, underscoring the need for rational combination strategies [19–21]. We therefore reasoned that co-targeting ribosome biogenesis with selinexor would synergize with osimertinib-mediated c-MYC suppression to cooperatively induce apoptosis.

Here, we show that osimertinib and selinexor cooperatively induce apoptosis in T-ALL cells in vitro and in vivo by disrupting ribosome biogenesis, which in turn triggers nucleolar stress and translational suppression, ultimately driving T-ALL cell apoptosis. This combination is effective at low concentrations in four genetically distinct T-ALL cell lines and in primary T-ALL blasts, and it retains a favorable safety window in normal cells. The combination also showed preclinical efficacy in a T-ALL xenograft model, supporting further evaluation in high-risk T-ALL. Moreover, our findings suggest c-MYC dependency and *TP53* status as potential stratification factors; treatment-induced c-MYC depletion and RPL5/RPL11 nuclear accumulation may serve as candidate pharmacodynamic biomarkers, which warrant further validation.

## Methods

### Cell lines and primary samples

Human T-ALL cell lines (MOLT-4, Jurkat, CCRF/CEM, and MOLT-3) were obtained from the American Type Culture Collection (ATCC; Manassas, VA, USA) and cultured in RPMI-1640 medium (C11875500BT, Gibco, USA) supplemented with 10% fetal bovine serum (FBS; FSP500, ExCell, Uruguay) and 1% penicillin/streptomycin (15140122, Gibco, USA). HEK293T and A549 cells (human non-small cell lung cancer cell line) were maintained in DMEM (12491015, Gibco, USA) with 10% FBS and 1% penicillin/streptomycin. All cell lines were authenticated by short tandem repeat (STR) profiling and tested negative for mycoplasma. Primary T-ALL blasts and normal human bone marrow mononuclear cells (BMMNCs) were isolated from bone marrow aspirates of newly diagnosed T-ALL patients and healthy donors, respectively, at Southwest Hospital affiliated to Army Medical University (approval no. (A) KY2026060), with written informed consent and institutional approval. BMMNCs were separated by Ficoll density gradient centrifugation. All experiments were performed with cells in logarithmic growth phase.

### Reagents and antibodies

Osimertinib (S7297, Selleck, USA) and selinexor (S7252, Selleck, USA) were dissolved in dimethyl sulfoxide (DMSO) and stored at -80℃. For in vivo studies, osimertinib and selinexor were formulated in corn oil, separately. Z-VAD-FMK (20 µM, HY-16658B), necrostatin-1 (Nec-1; 5 µM, HY-15760), ferrostatin-1 (Fer-1; 2 µM, HY-100579), MG132 (0.5 µM, HY-13259), cycloheximide (CHX; 5–10 µg/mL, HY-12320), pathway activators Yhhu-3792 (2.5 µM, HY-120782), 740 Y-P (30 µM, HY-P0175), and MHY1485 (10 µM, HY-B0795), and inhibitors LY294002 (10 µM, HY-10108), rapamycin (0.1 µM, HY-10219), and DAPT (10 µM, HY-13027) were purchased from MedChemExpress (MCE). EGF (100 ng/mL, HY-P7109, MCE) and puromycin (10 µg/mL, A23031, ABclonal) were used as indicated. All primary antibodies, along with their dilutions for immunoblotting, immunofluorescence, and immunohistochemistry, are listed in Supplementary Table 1. Secondary antibodies conjugated to HRP or fluorophores were from Beyotime (China).

### Cell proliferation assays

Jurkat, MOLT-4, CCRF/CEM, or MOLT-3 cells (1.5 × 10^4^ cells per well) were seeded in 96-well plates in 100 µL of cell suspension. For single-agent treatment, cells were incubated with increasing concentrations of osimertinib (0, 0.5, 1, 2, 3, 4, 5, 6, 7, 8, 9, 10 µM) for 24–48 h. For combination treatment, cells were incubated with osimertinib (0, 0.25, 0.5, 1, 2, 5 µM) plus a fixed dose of selinexor (20 nM) for 48 h. For necroptosis and ferroptosis inhibitor experiments, cells were pre-treated with Nec-1 (5 µM) or Fer-1 (2 µM) for 2 h and then treated with osimertinib (5 µM) for 48 h. Normal human BMMNCs and HEK293T cells were treated with vehicle, osimertinib (2, 5, or 10 µM) alone, selinexor (20, 50, or 100 nM) alone, or the corresponding combinations for 24–48 h. Then, 10 µL of CCK-8 reagent (BG0025, Bgbiotech, China) was added to each well and incubated for an additional 2–4 h. The optical density (OD) values were measured by a microplate reader at 450 nm. Cell viability was calculated as (OD treated − OD blank)/(OD control − OD blank) × 100%. IC_50_ values were calculated by non-linear regression using GraphPad Prism 9.0.

### Cell apoptosis assay

Four T-ALL cell lines (MOLT-4, Jurkat, CCRF/CEM, and MOLT-3) were treated for 24 h with osimertinib (0.5, 1, 2, 5, or 10 µM), selinexor (20 nM), or osimertinib (0.5, 1, or 2 µM) plus selinexor (20 nM). Primary T-ALL blasts were treated with osimertinib (5 or 10 µM) for 24 h. For Z-VAD-FMK rescue, cells were pre-treated with Z-VAD-FMK (20 µM) for 2 h and then treated with osimertinib (5 µM) or osimertinib (2 µM) plus selinexor (20 nM) for 24 h. For EGF rescue, cells were treated with EGF (100 ng/mL) alone or in combination with osimertinib (5 µM) for 24 h. For pathway modulation, cells were co-treated for 24 h with activators (740 Y-P, 30 µM; MHY1485, 10 µM; Yhhu-3792, 2.5 µM) or inhibitors (LY294002, 10 µM; rapamycin, 0.1 µM; DAPT, 10 µM) either alone or in combination with osimertinib (5 µM). After treatment, cells were washed with ice-cold PBS and incubated in 200 µL of binding buffer containing 10 µL of propidium iodide (PI) and 5 µL Annexin-V-APC (A214-01, Vazyme, China) for 15 min at room temperature in the dark. Cells were analyzed on a BD FACSCanto II flow cytometer, and the data were analyzed using FlowJo version 10. Apoptotic cells were defined as Annexin V-positive (early + late apoptosis).

### Flow cytometry

Mitochondrial membrane potential was assessed using tetramethylrhodamine ethyl ester (TMRE; C2001S, Beyotime, China). MOLT-4 and Jurkat cells were treated with osimertinib (5 or 10 µM) alone for 24 h, or with osimertinib (2 µM), selinexor (20 nM), or the combination for 24 h. Cells were then incubated with TMRE for 30 min at 37 °C. For in vivo engraftment assessment, bone marrow cells were stained with anti-human CD45 antibody (304005, BioLegend, China). All samples were acquired on a BD FACSCanto II flow cytometer and analyzed with FlowJo version 10.

### Immunoblotting

Cells were lysed in cold RIPA buffer (P0013, Beyotime, China) supplemented with protease and phosphatase inhibitor cocktails (P1045, Beyotime, China). Protein concentration was measured by bicinchoninic acid (BCA) assay (P0012, Beyotime, China). Equal amounts of proteins were separated by SDS-PAGE using One-Step PAGE Gel Fast Preparation Kit (PMK1014D, Biopmk, China) and transferred to PVDF membranes (IPFL00010, Merck, Germany). Membranes were blocked with non-fat milk in TBST and incubated overnight at 4 °C with primary antibodies. After washing, membranes were incubated with HRP-conjugated secondary antibodies for 1 h at room temperature. Blots were developed with ECL substrate (32109, Thermo, USA) and imaged on a ChemiDoc Touch system (Bio-Rad). Band intensities were quantified using Image Lab software and normalized to loading control (GAPDH or β-tubulin).

MOLT-4 and Jurkat cells were used for most immunoblotting experiments; CCRF/CEM and MOLT-3 cells were used for validation where indicated. For single-agent treatments, cells were incubated with osimertinib (5 or 10 µM) for 12–24 h. For combination treatments, cells were incubated with osimertinib (2 µM) plus selinexor (20 nM) for 12–24 h. For Z-VAD-FMK rescue, cells were pre-treated with Z-VAD-FMK (20 µM) for 2 h and then treated with osimertinib (5 µM) for 24 h. For pathway validation, cells were treated with activators (740 Y-P, 30 µM; MHY1485, 10 µM; Yhhu-3792, 2.5 µM) alone for 24 h to confirm target activation, or with inhibitors (LY294002, 10 µM; rapamycin, 0.1 µM; DAPT, 10 µM) alone or in combination with osimertinib (5 µM) for 24 h. For CHX chase, cells were treated with CHX (5 µg/mL) in the presence or absence of osimertinib (5 µM) for 0, 30, 60, or 90 min. For MG132 treatment, cells were pre-treated with MG132 (0.5 µM) for 2 h followed by osimertinib (5 µM) for 12 h. For nuclear-cytoplasmic fractionation, cells were lysed using Nuclear and Cytoplasmic Extraction Reagents (P0028, Beyotime, China) according to the manufacturer’s instructions; Lamin B and β-tubulin were used as nuclear and cytoplasmic loading controls, respectively. For mitochondrial/cytoplasmic fractionation, cells were processed using a Mitochondria Isolation Kit (C3601, Beyotime, China) following the manufacturer’s protocol. Cytochrome c was detected in both fractions by immunoblotting, with COX IV and GAPDH as mitochondrial and cytoplasmic markers, respectively, as previously described [22]. Vehicle and single-agent controls were included in all experiments.

### BAX activation assay

MOLT-4, Jurkat, CCRF/CEM, and MOLT-3 cells were treated with vehicle, osimertinib (2 µM), selinexor (20 nM), or the combination for 24 h. Activated BAX was detected by immunoblotting using the anti-BAX 6A7 monoclonal antibody (AB026, Beyotime, China), which specifically recognizes the conformational epitope of activated BAX, as previously described [22, 23]. GAPDH was used as a loading control.

### Puromycin incorporation assay

MOLT-4 and Jurkat cells were treated with vehicle, osimertinib (2 µM), selinexor (20 nM), or the combination (2 µM osimertinib + 20 nM selinexor) for 24 h. For the CHX alone group, cells were cultured for 12–24 h without drug treatment, and CHX (10 µg/mL) was added 1 h before sample collection as a positive control for translation inhibition. For the combination plus CHX group, cells were treated with the combination for 12–24 h, followed by CHX (10 µg/mL) 1 h before sample collection. Puromycin (10 µg/mL) was added to all groups 30 min before sample collection to label newly synthesized proteins. Cells were then lysed, and puromycin incorporation was detected by immunoblotting. GAPDH was used as a loading control.

### Immunofluorescence

MOLT-4 and Jurkat cells were treated with vehicle, osimertinib (2 µM), selinexor (20 nM), or the combination for 12 h. Cells were then cytospun onto glass slides, fixed with 4% paraformaldehyde for 15 min, permeabilized with 0.1% Triton X-100 for 10 min, and blocked with 5% normal goat serum/1% BSA for 1 h. Slides were incubated overnight at 4 °C with primary antibodies against RPS6, fibrillarin (FBL), or RPL5 (Supplementary Table 1). After washing, cells were incubated with fluorophore-conjugated secondary antibodies for 1 h at room temperature in the dark. Nuclei were counterstained with DAPI. Slides were mounted with anti-fade mounting medium (P0131, Beyotime, China). Fluorescence images were captured using a Leica DM4B fluorescence microscope. For quantification, at least 30 cells per group from three independent experiments were analyzed using ImageJ/Fiji. Nuclear/cytoplasmic mean fluorescence intensity (MFI) ratios for RPS6 and RPL5 were calculated. For FBL, total nuclear fluorescence intensity was measured to assess nucleolar stress, as previously described [8].

### Immunohistochemistry

Formalin-fixed, paraffin-embedded tissue sections  (4 μm) of liver and spleen were deparaffinized, rehydrated, and subjected to heat-induced antigen retrieval in EDTA buffer (1 mM EDTA, pH 8.0). Endogenous peroxidase was blocked with 3% H_2_O_2_, and non-specific binding was blocked with 5% normal serum/1% BSA. Sections were incubated overnight at 4 °C with primary antibodies against Ki-67, cleaved caspase-3, and c-MYC (Supplementary Table 1). HRP-conjugated polymer secondary antibodies were applied, and signals were developed with diaminobenzidine (DAB) substrate. Sections were counterstained with hematoxylin, dehydrated, and mounted. Stained slides were digitized using NDP.view2 (Hamamatsu, Japan). Quantification of positive cells was performed using ImageJ with the IHC Profiler plugin. For each section, three randomly selected, non-overlapping 20 × fields per section were analyzed. Percentage of positive cells was calculated. Naive non-leukemic NSG mice of the same age were used as histological controls.

### Quantitative real-time PCR

MOLT-4 and Jurkat cells were treated with vehicle or osimertinib (5 or 10 µM) for 24 h, or with vehicle, osimertinib (2 µM), selinexor (20 nM), or the combination for 24 h. Total RNA was extracted using the RNeasy Mini Kit (R0019M, Beyotime, China) and reverse transcribed with PrimeScript RT reagent Kit (RR036A, Takara, Japan) according to the manufacturer’s instructions. Quantitative PCR was performed on a CFX96 Touch Real-Time PCR Detection System (Bio-Rad) using SYBR Green qPCR Master Mix (K0221, Thermo, USA). Each reaction was run in triplicate. Relative gene expression was calculated by the 2^−ΔΔCt^ method using GAPDH as internal control. Primer sequences are listed in the Supplementary Table 2.

### Cellular thermal shift assay (CETSA)

CETSA was performed as previously described [24, 25]. Briefly, MOLT-4 cell lysates were prepared by freeze-thaw cycles in liquid nitrogen and centrifuged. For the temperature-gradient assay, equal aliquots were incubated with 80 µM osimertinib or vehicle control (DMSO) for 4 h at room temperature, then heated at indicated temperatures (37–55℃) for 3 min, cooled at room temperature for 3 min, and placed on ice. After centrifugation, soluble fractions were analyzed by immunoblotting for c-MYC. For the time-course assay, equal aliquots were incubated with 80 µM osimertinib or vehicle control at room temperature for 0, 0.5, 1, or 2 h, and soluble fractions were analyzed by immunoblotting for c-MYC.

### Drug affinity responsive target stability (DARTS)

DARTS assay was performed as previously described [26]. Briefly, MOLT-4 cell lysates were incubated with osimertinib (100 µM) for 1 h at room temperature, followed by digestion with pronase (1.25 mg/mL) at the indicated pronase-to-protein ratios (0, 1:200, 1:500, 1:1000, 1:2000, 1:5000) for 30 min. Reactions were terminated by adding loading buffer and boiling. Samples were analyzed by immunoblotting for c-MYC.

### In vivo xenograft studies

Six- to eight-week-old male NSG mice were purchased from Nanjing Jinzhihe Technology Co., Ltd. (Jiangsu, China) and housed under specific pathogen-free (SPF) conditions. All animal experiments were approved by the Institutional Animal Care and Use Committee of Daping Hospital, Army Medical University (approval no. AMUWEC20257056). Mice were injected intravenously via tail vein with 2 × 10^6^ MOLT-4 cells in 100 µL PBS (> 90% viability). Seven days post-engraftment, mice were randomized.

Two independent xenograft experiments were performed. In the first experiment, the single-agent activity of osimertinib was evaluated. Mice were randomized to receive vehicle (corn oil) or osimertinib (20 mg/kg, oral gavage, 5-days-on/2-days-off) from days 7 to 21. In the second experiment, mice were randomized to receive vehicle (corn oil), osimertinib (20 mg/kg, oral gavage, 5-days-on/2-days-off), selinexor (5 mg/kg, oral gavage, three times weekly), or the combination (osimertinib 10 mg/kg + selinexor 5 mg/kg) from days 7 to 21. In the combination group, osimertinib and selinexor were administered on the same schedules as the respective single agents.

For both experiments, two separate cohorts were used: one for survival analysis and one for predetermined endpoint analysis. For survival analysis, mice were monitored daily and euthanized upon reaching humane endpoints defined as > 20% body weight loss, hindlimb paralysis, severe respiratory distress, or inability to access food/water. Survival was analyzed by the Kaplan-Meier method. For endpoint analyses, a separate cohort of mice was euthanized on day 21 (treatment endpoint). Body weight was recorded every 2 days in this cohort. At endpoint, white blood cell counts, bone marrow hCD45^+^ frequency, spleen weight, histology, and immunohistochemistry were assessed. Naive non-leukemic NSG mice of the same age were used as histological controls. Spleen weights were recorded individually and presented as scatter plots with mean ± SD. Investigators were not blinded to group allocation during outcome assessment.

### Bioinformatics analysis

For transcriptomic analysis, MOLT-4 cells were treated with osimertinib (5 µM) for 16 h, with vehicle-treated cells as controls. RNA sequencing was performed by Shanghai Applied Protein Technology Co., Ltd. (Shanghai, China) using Illumina. For proteomic analysis, MOLT-4 cells were treated with osimertinib (2 µM) plus selinexor (20 nM) for 12 h, with vehicle-treated cells as controls. Proteomic analysis was performed by Beijing Qinglian Biotech Co., Ltd. (Beijing, China) using Bruker tims TOF HT. Differentially expressed genes/proteins were identified using |log₂ fold change| > 1.5 and adjusted *p* < 0.05. Kyoto Encyclopedia of Genes and Genomes (KEGG) pathway enrichment and Gene Set Enrichment Analysis (GSEA) were performed using Sangerbox 2.0 (http://www.sangerbox.com/tool) [27]. Enrichment was considered significant at FDR < 0.25 for GSEA and *p* < 0.05 for KEGG.

### Human protein atlas cell line database

The transcript expression profile of EGFR across hematopoietic cancer cell lines was obtained from the Human Protein Atlas (HPA) Cell Line resource (version 25.0) [28]. A total of 93 hematopoietic cancer cell lines with available EGFR expression data were included in the analysis. EGFR expression levels are reported as normalized transcripts per million (nTPM). Data were downloaded from https://www.proteinatlas.org.

### Statistical analysis

All statistical analyses were performed with GraphPad Prism (version 9.0). Data are presented as mean ± standard deviation (SD). All experiments were performed in at least three independent replicates, unless otherwise indicated. Comparisons between two groups were made using a two-tailed unpaired Student’s t test; multiple-group comparisons were assessed by one-way or two-way ANOVA with Tukey’s or Dunnett’s post hoc tests. Survival curves were generated by the Kaplan-Meier method and compared using the log-rank test. Drug synergy was quantified using SynergyFinder 2.0 with the Highest Single Agent (HSA) model; synergy scores > 10 were considered synergistic. A p value < 0.05 was considered statistically significant. Sample sizes and experimental replicates are indicated in the figure legends.

## Results

### Osimertinib induces apoptosis in T-ALL cells and suppresses leukemia progression in vivo

**Fig. 1:**
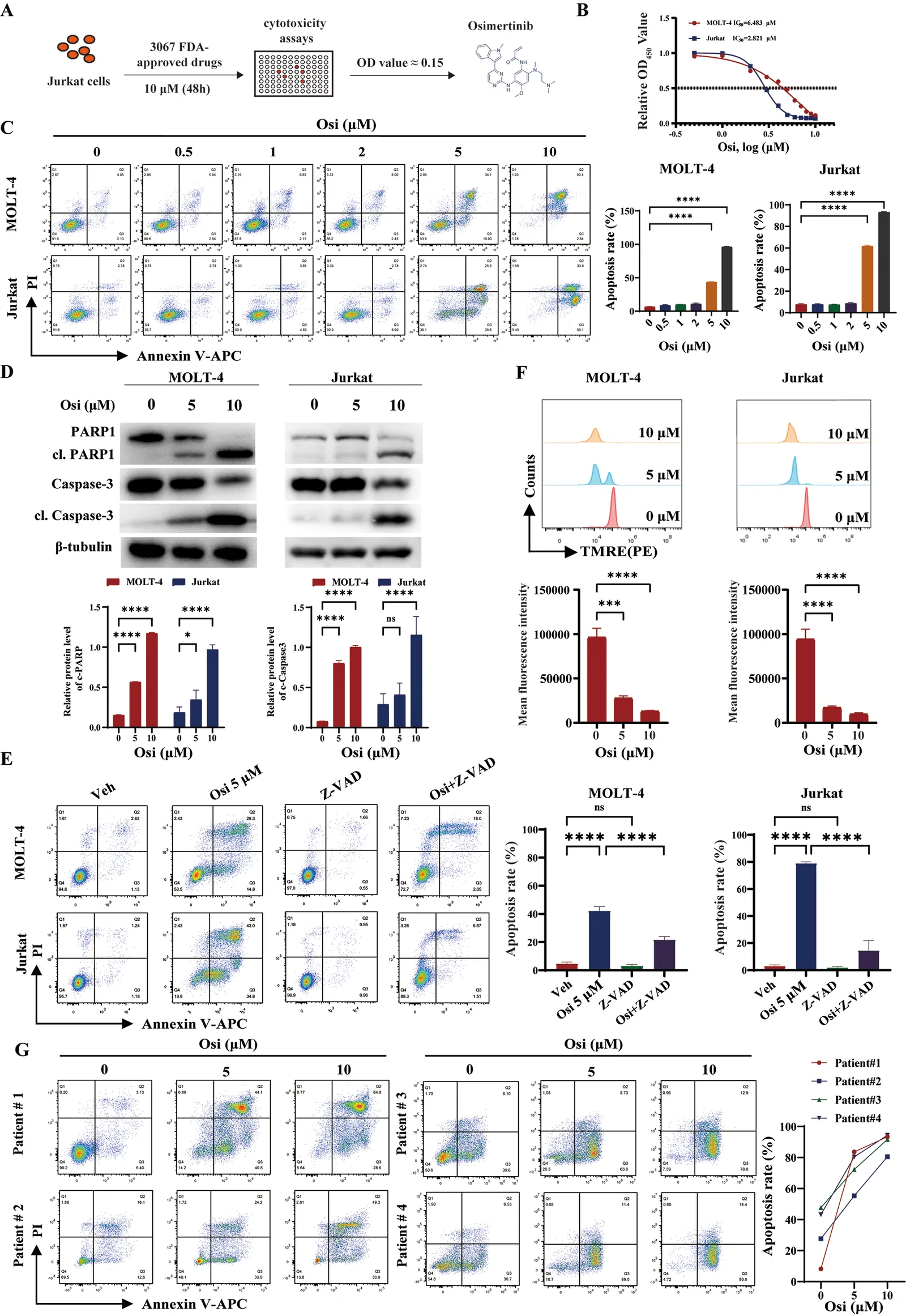
Osimertinib induces apoptosis in T-ALL cell lines and primary patient samples. (**A**) Schematic of the FDA-approved drug repurposing screen that identified osimertinib. (**B**) Dose-response curves and calculated IC_50_ values of osimertinib in MOLT-4 and Jurkat cells after 24 h of treatment. (**C**) Apoptosis measured by Annexin V-APC/PI staining in MOLT-4 and Jurkat cells treated with indicated concentrations of osimertinib (0, 0.5, 1, 2, 5, or 10 µM) for 24 h (n = 3). (**D**) Immunoblot analysis (top) and quantification (bottom) of cleaved PARP1 and cleaved Caspase-3 in MOLT-4 and Jurkat cells treated with osimertinib (0, 5, or 10 µM) for 12 h. β-tubulin, loading control (n = 3). (**E**) Apoptosis in MOLT-4 and Jurkat cells treated with vehicle, vehicle plus Z-VAD-FMK (20 µM), osimertinib (5 µM) alone, or osimertinib (5 µM) plus Z-VAD-FMK (20 µM). For Z-VAD-FMK-containing groups, cells were pretreated with Z-VAD-FMK for 2 h, followed by vehicle or osimertinib for an additional 24 h (n = 3). (**F**) Mitochondrial membrane potential assessed by TMRE staining (top) and quantification of mean fluorescence intensity (MFI, bottom) in MOLT-4 and Jurkat cells treated with osimertinib (0, 5, or 10 µM) for 24 h (n = 3). (**G**) Apoptosis measured by Annexin V-APC/PI staining in primary T-ALL blasts obtained from four newly diagnosed T-ALL patients and treated ex vivo with osimertinib (0, 5, or 10 µM) for 24 h. Data are presented as mean ± SD. *p < 0.05, **p < 0.01, ***p < 0.001, ****p < 0.0001; ns, not significant

To identify novel therapeutic agents for T-ALL, we performed a repurposing screen of an FDA-approved drug library comprising 3,067 compounds. This screen identified osimertinib, a third-generation EGFR tyrosine kinase inhibitor, as a potent candidate (Fig. 1A). We first evaluated the anti-leukemic activity of osimertinib in four T-ALL cell lines (MOLT-4, Jurkat, CCRF/CEM, and MOLT-3), which differ in *TP53* status: MOLT-3 is wild-type, Jurkat harbors a truncating R196* mutation, CCRF/CEM harbors R175H and R248Q missense mutations, and MOLT-4 harbors R248Q and R306* mutations [29, 30]. Osimertinib induced dose-dependent cytotoxicity in all four cell lines at 24 and 48 h, with half-maximal inhibitory concentration (IC_50_) values at 24 h of 6.5 µM in MOLT-4, 2.8 µM in Jurkat, 4.6 µM in CCRF/CEM, and 3.5 µM in MOLT-3, and lower IC_50_ values at 48 h (Fig. 1B; Supplementary Figure S1A). Low-dose osimertinib (0.5, 1, and 2 µM) alone induced minimal apoptosis, whereas 5 or 10 µM osimertinib significantly increased Annexin V-positive cells at 24 h and induced cleavage of PARP1 and caspase-3 in all four cell lines (Figs. 1 C and 1D; Supplementary Figures S1B and S1C). Cell death was caspase-dependent; the pan-caspase inhibitor Z-VAD-FMK significantly blocked osimertinib-induced apoptosis (Fig. 1E; Supplementary Figure S1D). In contrast, the necroptosis inhibitor Nec-1 and ferroptosis inhibitor Fer-1 did not rescue osimertinib-induced reduction in cell viability and neither rescued the growth inhibition (Supplementary Figure S1E). Osimertinib also disrupted mitochondrial membrane potential (ΔΨm), consistent with engagement of the intrinsic apoptotic pathway (Fig. 1F). Finally, osimertinib induced dose-dependent apoptosis in primary T-ALL blasts obtained from four newly diagnosed T-ALL patients (Fig. 1G).

**Fig. 2:**
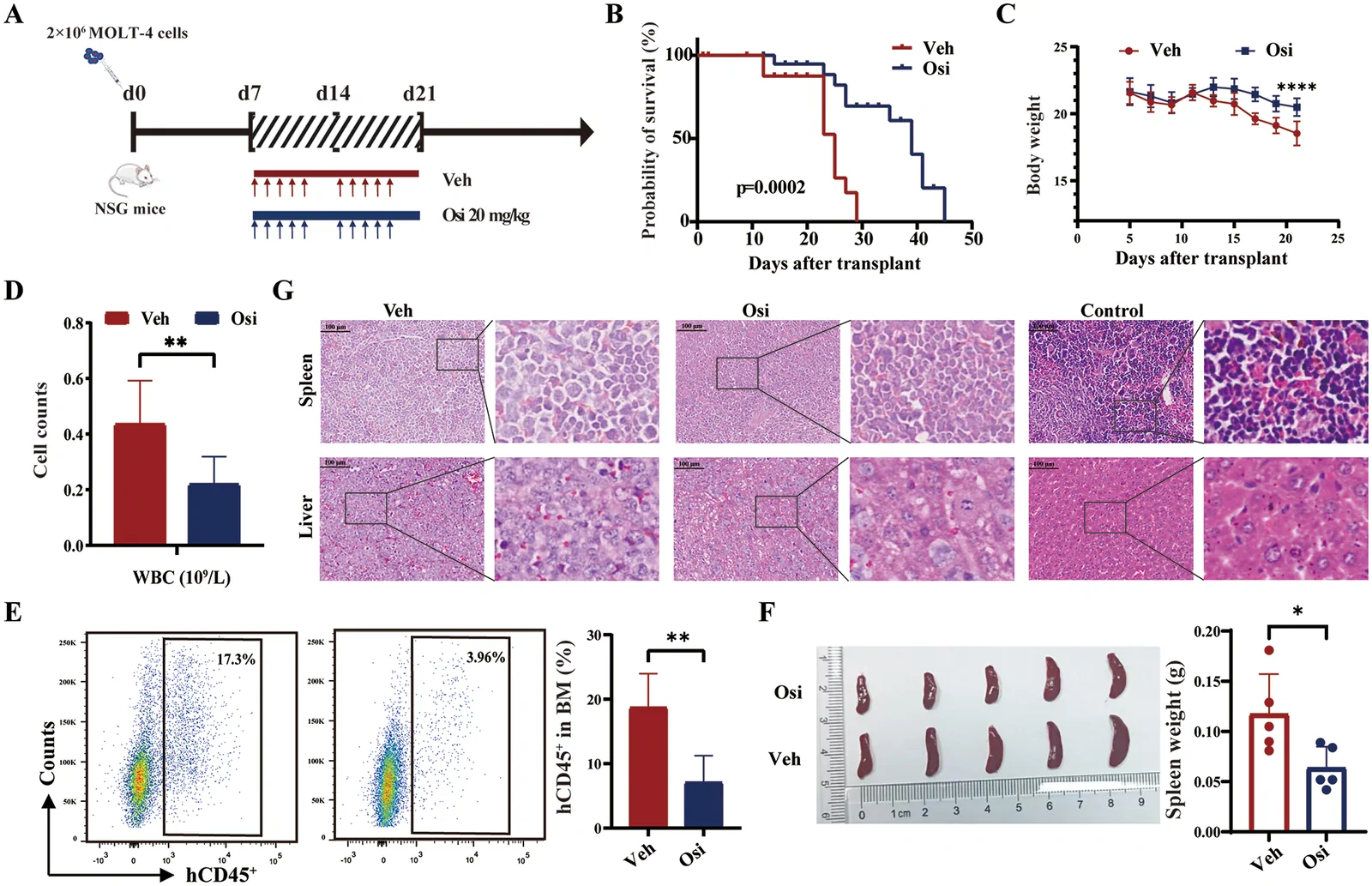
Osimertinib monotherapy suppresses leukemia progression and extends survival in vivo. (**A**) Schematic of the MOLT-4 cell-derived T-ALL xenograft model and treatment schedule. Mice were treated with vehicle (corn oil) or osimertinib (20 mg/kg, oral gavage) on a 5-days-on/2-days-off regimen from days 7 to 21 post-engraftment. (**B**) Kaplan-Meier survival curves of mice treated with vehicle (n = 12) or osimertinib (n = 11). Survival was monitored until humane endpoints (> 20% body weight loss, hindlimb paralysis, severe respiratory distress, or inability to access food/water; see Methods). (**C**-**G**) A separate cohort of mice (n = 5 per group) was used for body-weight monitoring (**C**) and endpoint analyses at day 21 (**D**-**G**); survival was assessed in an independent cohort (**B**). (**C**) Body-weight change over time of mice treated with vehicle (n = 5) or osimertinib (n = 5). (**D**) White blood cell counts at endpoint (n = 5 mice per group). (**E**) Flow cytometric analysis (left) and quantification (right) of hCD45^+^ leukemia cells in bone marrow at endpoint (n = 5 mice per group). (**F**) Representative spleen images (left) and individual spleen-weight quantification (right) at endpoint (n = 5 mice per group). (**G**) Representative H&E-stained sections of spleen and liver at endpoint (scale bars, 100 μm). Naive non-leukemic NSG mice were used as histological controls. Data are presented as mean ± SD. *p < 0.05, **p < 0.01, ***p < 0.001, ****p < 0.0001; ns, not significant

We next evaluated the in vivo efficacy of osimertinib in a MOLT-4 xenograft model (Fig. 2A). Mice engrafted with MOLT-4 cells were randomized to receive osimertinib (20 mg/kg) or vehicle on a 5-days-on/2-days-off regimen from days 7 to 21 post-engraftment. In the survival cohort, osimertinib treatment prolonged overall survival (Fig. 2B). In a separate endpoint analysis cohort, body weight remained stable throughout the treatment period (Fig. 2C). At endpoint (day 21), osimertinib-treated mice showed lower white blood cell (WBC) counts compared with vehicle-treated controls (Fig. 2D). The treatment also reduced bone marrow leukemic burden, as measured by a lower frequency of hCD45^+^ cells (Fig. 2E). Treated mice exhibited a reduction in spleen size and weight (Fig. 2F). Individual spleen weights are shown as scatter plots, confirming that the difference was not driven by a single outlier (Fig. 2F, right). Sensitivity analysis excluding the largest vehicle spleen confirmed that the reduction in spleen weight remained significant (*p* = 0.0332). Histologic examination (H&E) revealed decreased leukemic infiltration in the liver and spleen of osimertinib-treated mice compared with vehicle controls, with naive non-leukemic NSG mice used as histological references (Fig. 2G).

### Osimertinib induces c-MYC destabilization through a non-canonical, EGFR-independent mechanism

**Fig. 3:**
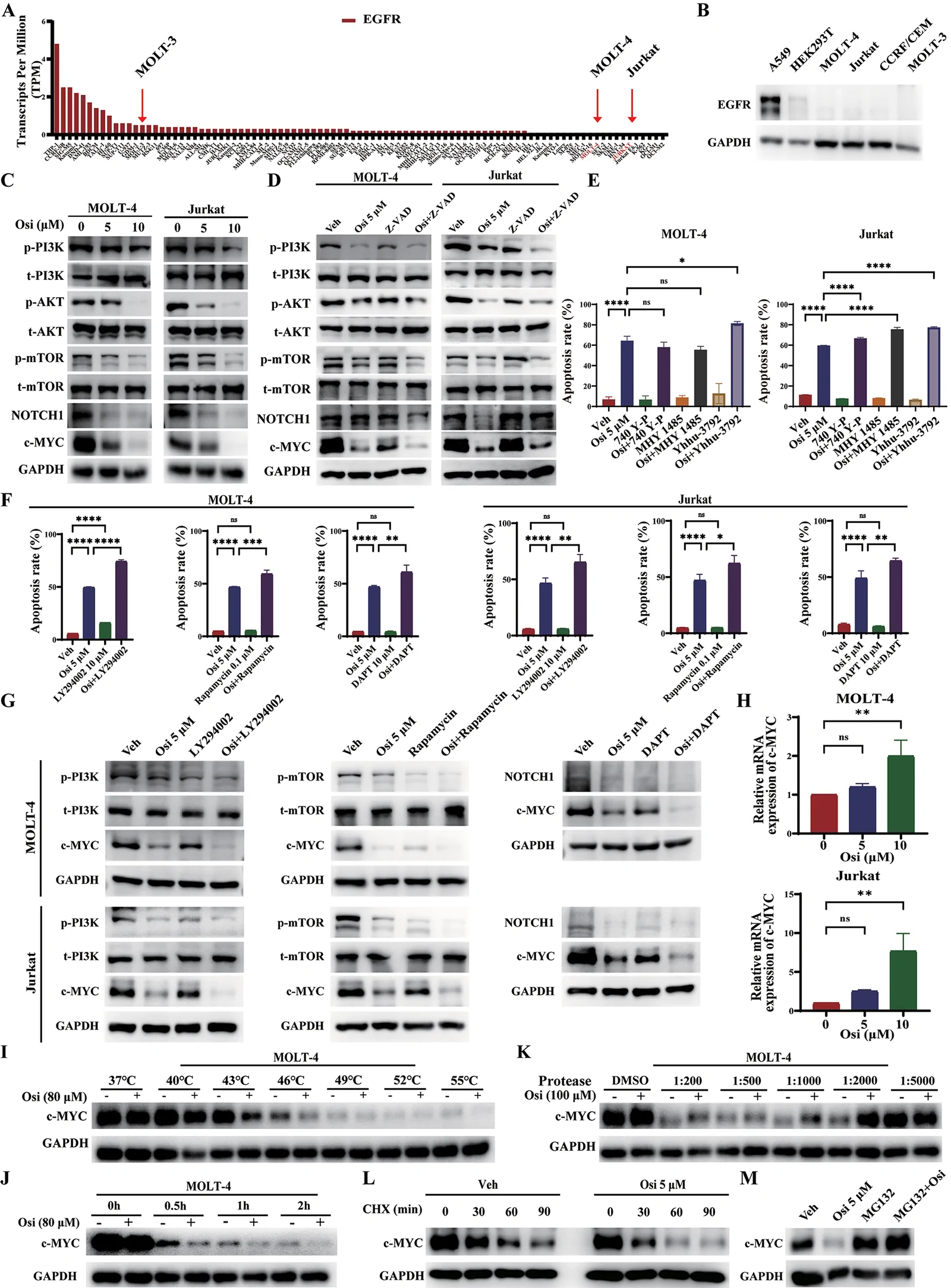
Osimertinib induces c-MYC destabilization through a non-canonical, EGFR-independent mechanism. (**A**) RNA expression levels of EGFR across 93 hematopoietic cancer cell lines from the HPA Cell Line resource. (**B**) Immunoblot analysis of EGFR in A549, HEK293T, MOLT-4, Jurkat, CCRF/CEM and MOLT-3 cells. GAPDH, loading control. (**C**) Immunoblot analysis of key signaling proteins and c-MYC in MOLT-4 and Jurkat cells treated with osimertinib (0, 5, or 10 µM) for 12 h. GAPDH, loading control (n = 3). (**D**) Immunoblot analysis of key signaling proteins and c-MYC in MOLT-4 and Jurkat cells pre-treated with Z-VAD-FMK (20 µM, 2 h) prior to osimertinib (5 µM) treatment for 24 h. GAPDH, loading control (n = 3). (**E**) Apoptosis analysis by flow cytometry in MOLT-4 and Jurkat cells treated with osimertinib (5 µM) alone or in combination with pathway activators 740 Y-P (30 µM), MHY1485 (10 µM), or Yhhu-3792 (2.5 µM) for 24 h (n = 3). (**F**) Apoptosis analysis by flow cytometry in MOLT-4 and Jurkat cells treated with osimertinib (5 µM) alone or in combination with pathway inhibitors LY294002 (10 µM), rapamycin (0.1 µM), or DAPT (10 µM) for 24 h (n = 3). (**G**) Immunoblot analysis of key signaling proteins and c-MYC in MOLT-4 and Jurkat cells treated with the indicated inhibitors alone or in combination with osimertinib as in (F). GAPDH, loading control(n = 3). (**H**) RT-qPCR analysis of c-MYC mRNA expression in MOLT-4 and Jurkat cells treated with osimertinib (0, 5, or 10 µM) for 24 h (n = 3). (**I**) CETSA measuring c-MYC thermal stability after incubation with osimertinib (80 µM) at the indicated temperatures. GAPDH, loading control (n = 3). (**J**) Time-course CETSA showing osimertinib-induced c-MYC degradation at room temperature. GAPDH, loading control (n = 3). (**K**) DARTS assay determining c-MYC stability at different protease-to-protein ratios in the presence of osimertinib (100 µM). GAPDH, loading control (n = 3). (**L**) Cycloheximide (CHX, 5 µg/mL) chase assay examining the effect of osimertinib (5 µM) on c-MYC protein stability for 0, 30, 60, and 90 min. GAPDH, loading control (n = 3). (**M**) Pre-treatment with the proteasome inhibitor MG132 (0.5 µM, 2 h) abrogated osimertinib (5 µM)-induced c-MYC degradation. GAPDH, loading control (n = 3). Data are presented as mean ± SD. *p < 0.05, **p < 0.01, ***p < 0.001, ****p < 0.0001; ns, not significant

To delineate the mechanism of action, we first examined whether osimertinib engages its canonical target, EGFR, in T-ALL cells. Analysis of the HPA Cell Line database (version 25.0) revealed minimal EGFR transcript abundance in MOLT-4, Jurkat and MOLT-3 cells (TPM < 1) (Fig. 3A). CCRF/CEM was not included in the HPA Cell Line database. Concordantly, immunoblot analysis showed minimal EGFR protein expression in all four T-ALL cell lines (MOLT-4, Jurkat, CCRF/CEM, and MOLT-3) relative to A549 and HEK293T cells (Fig. 3B). Addition of the EGFR ligand EGF did not rescue osimertinib-induced apoptosis (Supplementary Figure S2A).

Transcriptomic analysis of osimertinib-treated MOLT-4 revealed coordinated downregulation of gene sets associated with PI3K/AKT/mTOR and NOTCH1 signaling, as well as MYC targets (Supplementary Figures S2B and S2C; Supplementary file 2). Immunoblot analysis confirmed that osimertinib dose-dependently inhibited phosphorylation of PI3K, AKT, and mTOR, and reduced protein levels of NOTCH1 and c-MYC in MOLT-4 and Jurkat cells (Fig. 3C; quantification in Supplementary Figure S2D), with similar results in CCRF/CEM and MOLT-3 cells (Supplementary Figure S2E). To exclude the possibility that these effects were secondary to cell death, we pre-treated cells with Z-VAD-FMK before osimertinib addition. Z-VAD-FMK did not prevent the reduction of p-PI3K, p-AKT, p-mTOR, NOTCH1, or c-MYC (Fig. 3D; quantification in Supplementary Figure S2F; additional cell lines in Supplementary Figure S2G), indicating that these changes were not secondary to caspase-dependent apoptosis.

We then asked whether c-MYC depletion depends on PI3K/AKT/mTOR or NOTCH1 signaling. First, co-treatment with activators of PI3K (740 Y-P), mTOR (MHY1485), or NOTCH1 (Yhhu-3792) did not reverse osimertinib-induced apoptosis (Fig. 3E; flow cytometry plots in Supplementary Figure S3A). The activators effectively increased p-PI3K, p-mTOR, or NOTCH1 levels, but did not upregulate c-MYC protein (Supplementary Figure S3B). Second, cells were co-treated with the PI3K inhibitor LY294002 (10 µM), the mTOR inhibitor rapamycin (0.1 µM), or the NOTCH1 inhibitor DAPT (10 µM) together with osimertinib (5 µM) for 24 h. Pathway inhibition was confirmed by reduced p-PI3K, p-mTOR, or NOTCH1 levels (Fig. 3G; Supplementary Figure S3D). These inhibitors alone partially reduced c-MYC, consistent with the roles of these pathways in c-MYC regulation. However, osimertinib alone reduced c-MYC to a greater extent, and co-treatment with each inhibitor plus osimertinib did not further significantly reduce c-MYC compared with osimertinib alone (Fig. 3G; Supplementary Figure S3D). Although these inhibitors alone did not induce apoptosis, they enhanced osimertinib-induced apoptosis (Fig. 3F; Supplementary Figure S3C), suggesting that inhibition of these pathways may decrease the apoptotic threshold without further reducing c-MYC.

Finally, to determine whether c-MYC is regulated at the transcriptional level, we measured c-MYC mRNA by RT-qPCR. Osimertinib did not reduce c-MYC mRNA levels; rather, c-MYC mRNA was slightly increased at 5 µM and significantly increased at 10 µM in both MOLT-4 and Jurkat cells (Fig. 3H), despite marked depletion of c-MYC protein, indicating post-transcriptional regulation.

We therefore examined c-MYC protein stability. CETSA and DARTS assays showed that osimertinib altered the thermal stability and protease sensitivity of c-MYC, suggesting a potential direct interaction or conformational change (Fig. 3I-K; quantification in Supplementary Figures S3E-S3G). A cycloheximide (CHX) chase assay confirmed that osimertinib shortened the half-life of c-MYC, and pre-treatment with the proteasome inhibitor MG132 blocked osimertinib-induced c-MYC degradation (Fig. 3L and M; quantification in Supplementary Figures S3H and S3I). Together with the lack of EGFR expression and the inability of EGF to rescue apoptosis, these data indicate a non-canonical, EGFR-independent mechanism for osimertinib-induced c-MYC degradation, although the direct molecular target remains to be identified.

### Osimertinib-induced ribosome biogenesis vulnerability synergizes with selinexor

**Fig. 4:**
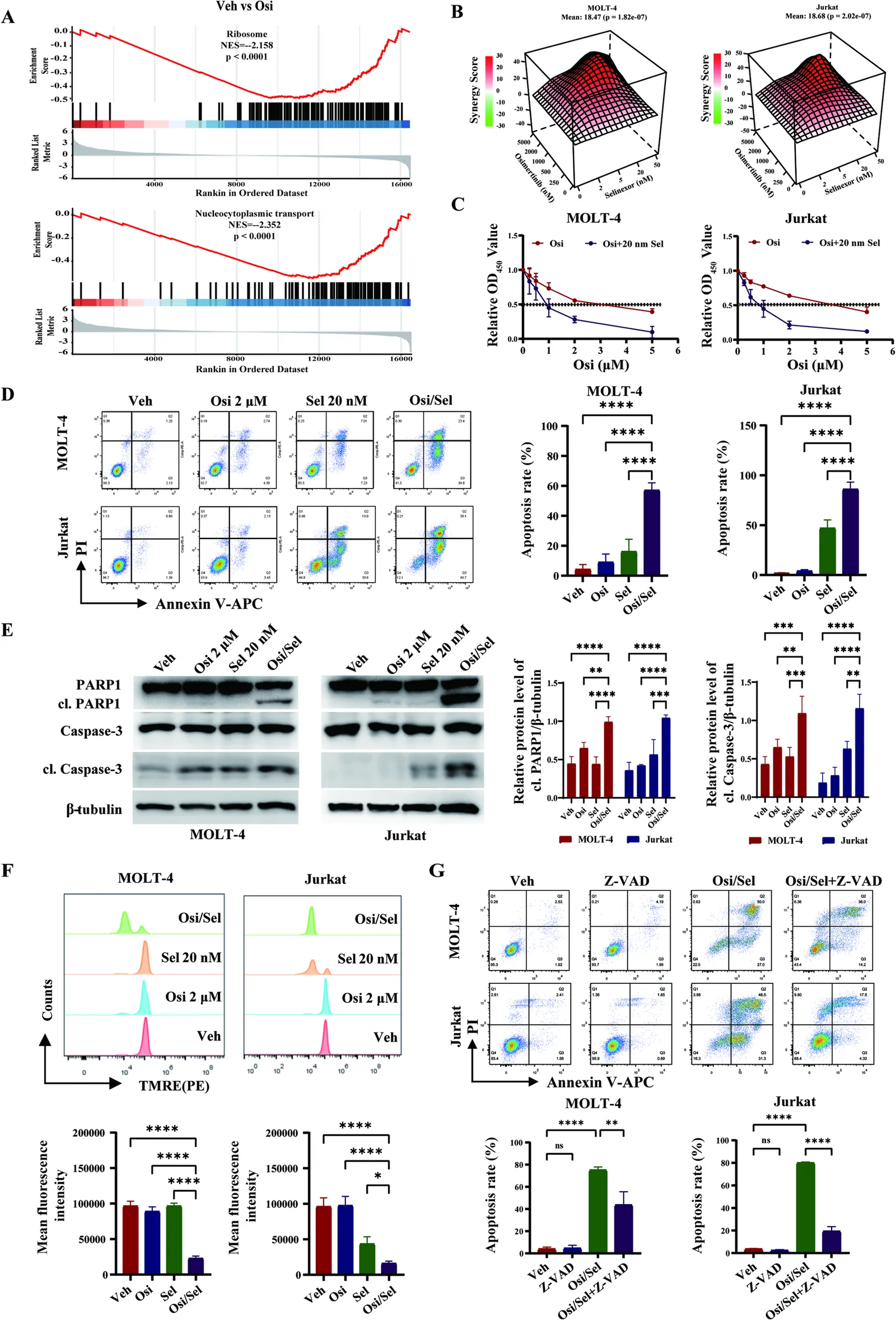
Cotargeting ribosomal biogenesis synergizes with selinexor. (**A**) GSEA enrichment analysis of transcriptomic data from MOLT-4 cells treated with 5 µM osimertinib for 16 h, showing significant downregulation of ribosome-biogenesis gene sets. (**B**) Dose-response matrix analysis (HSA model) quantifying the synergistic effect of osimertinib and selinexor in MOLT-4 and Jurkat cells. Synergy scores are indicated. (**C**) Dose-response curves of osimertinib alone or combined with a fixed dose of selinexor (20 nM) in MOLT-4 and Jurkat cells. Dashed lines indicate IC_50_ values. (**D**) Apoptosis analysis by Annexin V-APC/PI staining in MOLT-4 and Jurkat cells treated for 24 h with vehicle, osimertinib (2 µM) alone, selinexor (20 nM) alone, or the combination (n = 3). (**E**) Immunoblot analysis (left) and quantification (right) of cleaved PARP1 and cleaved Caspase-3 in MOLT-4 and Jurkat cells treated as in (**D**). β-tubulin, loading control (n = 3). (**F**) Mitochondrial membrane potential assessed by TMRE staining (top) and quantification of mean fluorescence intensity (MFI, bottom) in MOLT-4 and Jurkat cells treated as in (**D**) (n = 3). (**G**) Apoptosis in MOLT-4 and Jurkat cells treated with vehicle, vehicle plus Z-VAD-FMK (20 µM), the combination, or the combination plus Z-VAD-FMK (20 µM). For Z-VAD-FMK-containing groups, cells were pretreated with Z-VAD-FMK (20 µM) for 2 h, followed by vehicle or the combination for an additional 24 h (n = 3). Data are presented as mean ± SD. *p < 0.05, **p < 0.01, ***p < 0.001, ****p < 0.0001; ns, not significant

Given the central role of c-MYC in driving ribosome biogenesis, we examined whether osimertinib-mediated c-MYC suppression impairs this process. Transcriptomic analysis of osimertinib-treated cells revealed marked downregulation of gene sets involved in ribosome biogenesis (Fig. 4A; Supplementary file 2), suggesting a vulnerability that could be exploited by blocking nuclear export of ribosomal subunits.

We therefore combined osimertinib with selinexor, an XPO1 inhibitor that retains ribosomal subunits in the nucleus. Dose-response matrix analysis revealed synergistic cytotoxicity in four T-ALL cell lines (MOLT-4, Jurkat, CCRF/CEM, and MOLT-3), with HSA synergy scores of 18.47, 18.68, 11.20 and 16.03, respectively (Fig. 4B; Supplementary Figure S4A). A fixed low dose of selinexor (20 nM) markedly reduced the IC_50_ of osimertinib in all four cell lines (Fig. 4C; Supplementary Figure S4B).

At 2 µM, the combination of osimertinib and selinexor induced greater apoptosis than either single agent alone in all four T-ALL cell lines (Fig. 4D; Supplementary Figures S4C). This was accompanied by enhanced cleavage of PARP1 and caspase-3 (Fig. 4E; Supplementary Figure S4D) and a marked loss of mitochondrial membrane potential in MOLT-4 and Jurkat cells (Fig. 4F). Then, we tested the effect of drug combination adopting lower concentrations of osimertinib. At 1 µM of osimertinib, the combination induced greater apoptosis than either single agent alone in three of four cell lines (MOLT-4, MOLT-3, and CCRF/CEM). At 0.5 µM of osimertinib, the combination induced greater apoptosis than either single agent alone in two of four cell lines (MOLT-4 and MOLT-3). In the remaining cell lines, the low-concentration combination increased apoptosis compared with osimertinib alone but did not exceed selinexor alone (Supplementary Figures S4C and S4E). These data indicate that clear synergy is achieved at 2 µM in all four cell lines, while lower concentrations primarily enhance the effect of osimertinib in a subset of cell lines. These differential responses among the four cell lines suggest that genetic background, including *TP53* status, may influence sensitivity to the combination.

To determine whether combination-induced cell death is caspase-dependent, cells were pre-treated with Z-VAD-FMK before osimertinib plus selinexor treatment. In MOLT-4, Jurkat, and MOLT-3 cells, Z-VAD-FMK significantly reduced the apoptotic rate induced by the combination (Fig. 4G; Supplementary Figure S4F), indicating that the combination triggers caspase-dependent apoptosis in these cell lines. In CCRF/CEM cells, the combination (2 µM osimertinib plus 20 nM selinexor) significantly increased apoptosis compared with single agents, but Z-VAD-FMK did not further reduce apoptosis (Supplementary Figure S4F), suggesting that combination-induced cell death in this cell line is largely caspase-independent.

### Selectivity of the selinexor-osimertinib combination in normal cells

To assess whether the combination affects normal cells, we evaluated its cytotoxicity in normal human bone marrow mononuclear cells (BMMNCs) and HEK293T cells. At 2 µM osimertinib plus 20 nM selinexor, which showed clear synergy in all four T-ALL cell lines, the combination did not markedly reduce viability of either cell type, with approximately 85% viability in BMMNCs and 75% in HEK293T cells after 48 h. Higher concentrations (5 µM + 50 nM and 10 µM + 100 nM) reduced viability in a dose-dependent manner, but the effect was comparable to osimertinib alone at the same concentrations, indicating no synergistic toxicity (Supplementary Figure S5). These findings suggest that the combination retains a favorable safety window at low doses.

### The selinexor-osimertinib combination improves survival in T-ALL cell xenograft models

**Fig. 5:**
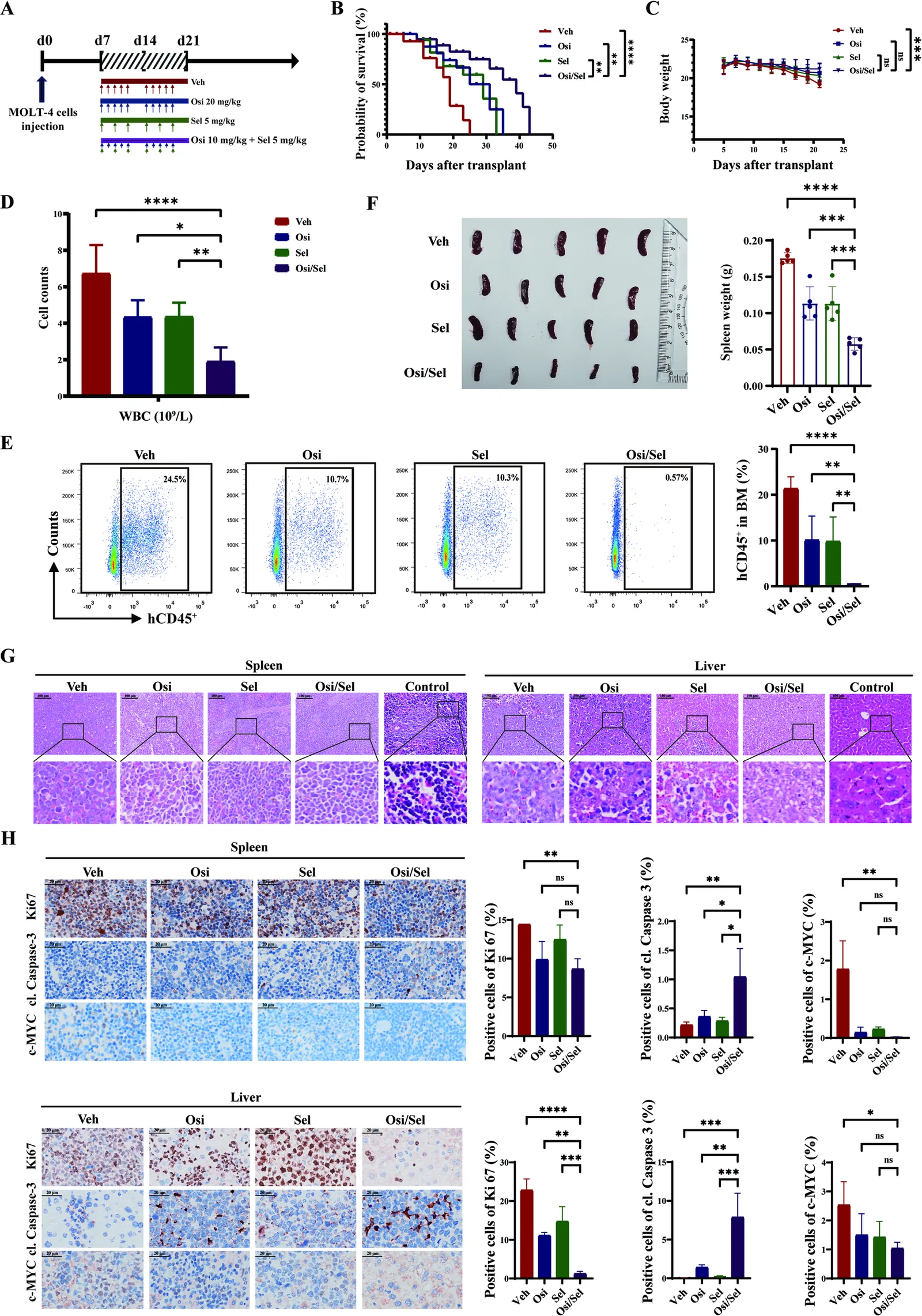
The selinexor-osimertinib combination improves survival in T-ALL xenograft models. (**A**) Schematic of the treatment schedule. Mice bearing MOLT-4 T-ALL xenografts were treated with vehicle (corn oil), osimertinib (20 mg/kg, 5-days-on/2-days-off), selinexor (5 mg/kg, three times weekly), or the combination (osimertinib 10 mg/kg, 5-days-on/2-days-off + selinexor 5 mg/kg, three times weekly) from days 7 to 21 post-engraftment. (**B**) Kaplan-Meier survival curves (n = 10 mice per group). Survival was monitored until humane endpoints (> 20% body weight loss, hindlimb paralysis, severe respiratory distress, or inability to access food/water; see Methods). (**C**-**H**) A separate cohort of mice (n = 5 per group) was used for body-weight monitoring (**C**) and endpoint analyses at day 21 (**D**-**H**); survival was assessed in an independent cohort (**B**). (**C**) Body-weight change over time (n = 5 mice per group). (**D**) White blood cell counts at endpoint (n = 5 mice per group). (**E**) Flow cytometric analysis (left) and quantification (right) of hCD45^+^ leukemia cells in bone marrow at endpoint (n = 5 mice per group). (**F**) Representative spleens images (left) and individual spleen-weight quantification (right) at endpoint (n = 5 mice per group). Bone marrow was collected on day 21 from the endpoint cohort. (**G**) Representative H&E-stained sections of spleen and liver at endpoint (scale bars, 100 μm). Naive non-leukemic NSG mice were used as histological controls. (**H**) Representative immunohistochemical staining and quantification of cleaved Caspase-3, Ki-67, and c-MYC in spleen and liver at endpoint (scale bars, 20 μm). Quantification of positive cells is provided in the right panels (n = 3 fields per group). Data are presented as mean ± SD. *p < 0.05, **p < 0.01, ***p < 0.001, ****p < 0.0001; ns, not significant

We next evaluated the therapeutic potential of the combination in T-ALL xenograft models. Mice bearing MOLT-4 xenografts were randomized to receive vehicle, osimertinib, selinexor, or the combination (Fig. 5A). The combination group showed the greatest survival benefit, significantly outperforming both monotherapies (Fig. 5B). Vehicle-treated mice lost weight due to progressive disease, whereas the combination group maintained stable body weight (Fig. 5C). No marked treatment-related body-weight loss or overt clinical signs of toxicity were observed during the treatment period.

Peripheral blood analysis revealed markedly reduced WBC counts in combination-treated mice compared with vehicle or single-agent groups (Fig. 5D). The combination also most effectively reduced bone marrow leukemic burden, as measured by the lowest frequency of hCD45^+^ cells (Fig. 5E), and alleviated splenomegaly, with the greatest reduction in spleen size and weight (Fig. 5F). Individual spleen weights are shown as scatter plots (Fig. 5F, right). Bone marrow was collected on day 21 from a separate endpoint cohort, while survival was monitored in an independent cohort until humane endpoints (see Methods). The reduction in bone marrow leukemic burden therefore reflects early treatment response and does not exclude residual extramedullary disease.

Histologic assessment confirmed the most pronounced reduction in leukemic infiltration in the combination group (Fig. 5G). Naive non-leukemic NSG mice were used as histological controls (Fig. 5G, Control). Immunohistochemical analysis of spleen and liver tissues from combination-treated mice revealed increased cleaved caspase-3 levels and decreased Ki-67 positivity in both organs (Fig. 5H). Quantification of these markers confirmed that cleaved caspase-3 was significantly increased in the combination group compared with vehicle or single-agent groups in both spleen and liver, and Ki-67 was significantly decreased in the combination group in both organs. In both spleen and liver, c-MYC expression was significantly reduced in the combination group compared with vehicle, but did not differ significantly from either single agent (Fig. 5H, right).

### The selinexor-osimertinib combination induces nucleolar stress, c-MYC depletion, and translational suppression

**Fig. 6:**
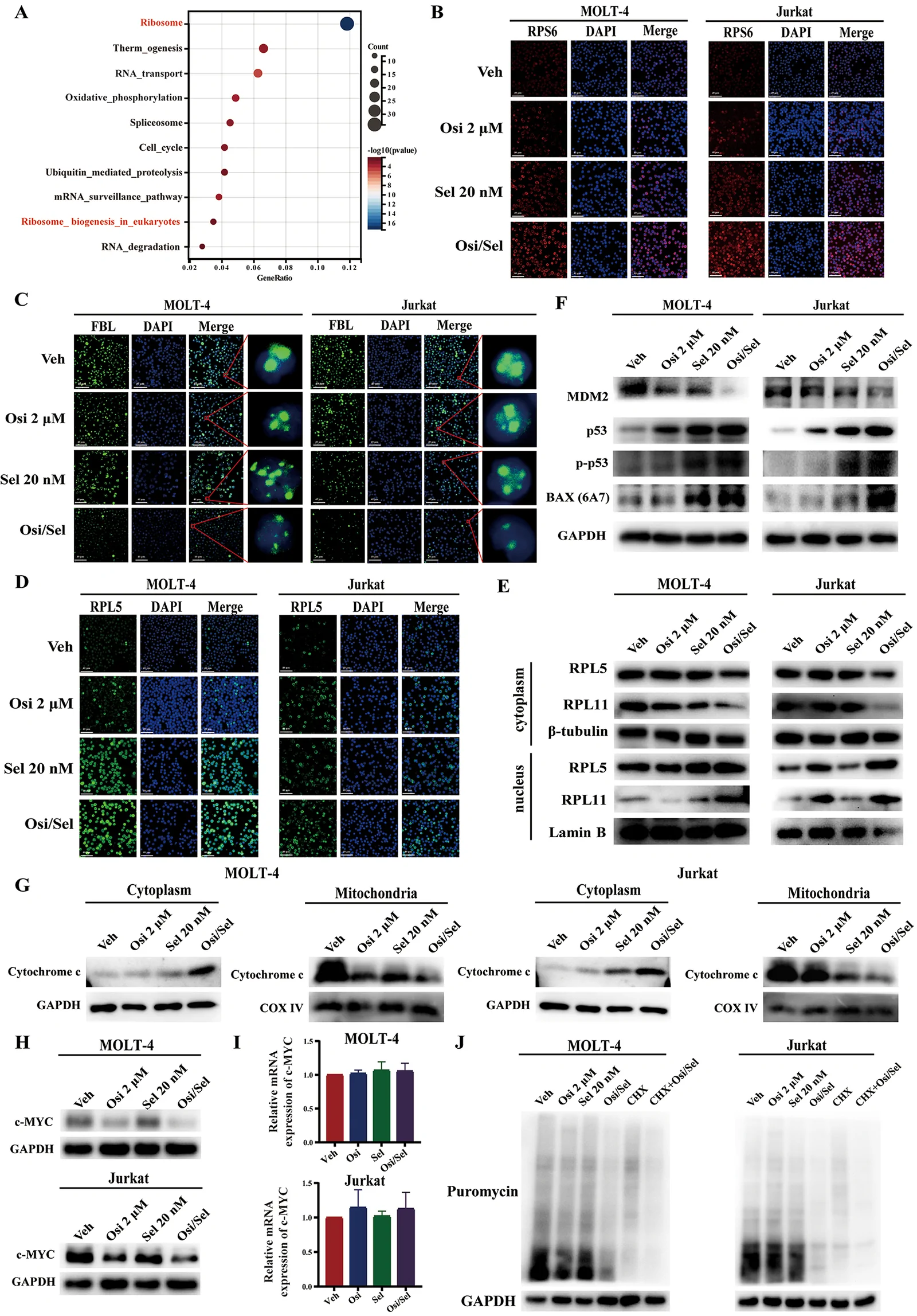
The selinexor-osimertinib combination induces nucleolar stress, translational suppression, and c-MYC depletion. (**A**) KEGG pathway enrichment analysis of MOLT-4 cells treated with the combination (osimertinib 2 µM + selinexor 20 nM) versus vehicle controls. (**B**) Representative immunofluorescence images showing the subcellular localization of RPS6 in MOLT-4 and Jurkat cells treated with vehicle, osimertinib (2 µM), selinexor (20 nM), or the combination for 12 h (scale bars, 40 μm). (**C**) Representative immunofluorescence images showing nucleolar morphology (marked by fibrillarin/FBL) in MOLT-4 and Jurkat cells treated as in (**B**) (scale bars, 40 μm), with enlarged views. (**D**) Representative immunofluorescence images showing the subcellular localization of RPL5 in MOLT-4 and Jurkat cells treated as in (**B**) (scale bars, 40 μm). (**E**) Immunoblot analysis of nuclear/cytoplasmic RPL5 and RPL11 in MOLT-4 and Jurkat cells treated as in (**B**). Lamin B and β-tubulin, loading controls (n = 3). (**F**) Immunoblot analysis of MDM2, p53, phosphorylated p53 (p-p53), and activated BAX (6A7) in MOLT-4 and Jurkat cells treated as in (**B**). GAPDH, loading control (n = 3). (**G**) Immunoblot analysis of cytoplasmic and mitochondrial cytochrome c levels in MOLT-4 and Jurkat cells treated as in (**B**). GAPDH and COX IV were used as cytoplasmic and mitochondrial loading controls, respectively. (**H**) Immunoblot analysis of c-MYC in MOLT-4 treated for 12 h and Jurkat cells treated for 24 h as in (**B**). GAPDH, loading control (n = 3). (**I**) RT-qPCR analysis of c-MYC mRNA expression in MOLT-4 and Jurkat cells treated as in (**B**) for 24 h (n = 3). (**J**) Immunoblot analysis of global protein synthesis assessed by puromycin incorporation in MOLT-4 and Jurkat cells treated with vehicle, osimertinib (2 µM), selinexor (20 nM), the combination, cycloheximide (CHX), or the combination plus CHX. Puromycin was added to all groups 30 min before sample collection. CHX (10 µg/mL) was added 1 h before sample collection to the CHX and the combination + CHX groups as positive controls for translation inhibition. GAPDH, loading control (n = 3). Data are presented as mean ± SD. *p < 0.05, **p < 0.01, ***p < 0.001, ****p < 0.0001; ns, not significant

To delineate the mechanistic basis of the synergy, we performed proteomic analysis. Global pathway analysis revealed significant downregulation of ribosome biogenesis pathways (Fig. 6A; Supplementary file 3).

Consistent with XPO1 inhibition, both single agents showed increased nuclear/cytoplasmic RPS6 ratios, with the greatest increase observed in the combination group (Fig. 6B). Quantification of nuclear/cytoplasmic RPS6 ratios confirmed these observations (Supplementary Figure S6A). We observed nucleolar stress, characterized by fibrillarin (FBL) dispersal and loss of nucleolar integrity (Fig. 6C). Total FBL fluorescence intensity in the combination group was significantly lower compared with the vehicle group in both cell lines; in Jurkat cells it was also significantly lower than osimertinib alone, whereas in MOLT-4 cells the difference between the combination group and osimertinib alone or selinexor alone was not significant (Supplementary Figure S6B). Nuclear accumulation of RPL5 and RPL11 was also observed (Fig. 6D and Supplementary Figure S6C for RPL5 immunofluorescence; Fig. 6E and Supplementary Figure S6D for RPL5/RPL11 immunoblotting).

The accumulation of RPL5 and RPL11 was accompanied by decreased MDM2 protein levels, increased stabilization and phosphorylation of p53, and BAX activation, in all four T-ALL cell lines (MOLT-4 and Jurkat in Fig. 6F and Supplementary Figure S6E; CCRF/CEM and MOLT-3 in Supplementary Figure S7A). Furthermore, cytoplasmic cytochrome c levels were significantly increased in the combination group in all four T-ALL cell lines (MOLT-4 and Jurkat in Fig. 6G and Supplementary Figure S6F; CCRF/CEM and MOLT-3 in Supplementary Figure S7B), indicating mitochondrial outer membrane permeabilization.

The combination also caused rapid post-transcriptional depletion of c-MYC. c-MYC protein was significantly downregulated in the combination group after 12 h in MOLT-4 cells and after 24 h in Jurkat, CCRF/CEM, and MOLT-3 cells (Fig. 6H; Supplementary Figures S6G and S7C). In contrast, the combination did not significantly reduce c-MYC mRNA levels (Fig. 6I), indicating post-transcriptional regulation. Global protein synthesis was reduced, as shown by decreased puromycin incorporation (Fig. 6J; Supplementary Figure S6H).

## Discussion

T-ALL remains a challenging malignancy with poor outcomes, particularly in relapsed or refractory settings, highlighting the need for novel therapeutic strategies [2, 31]. Here, we identified osimertinib, a third-generation EGFR inhibitor, as a potent anti-T-ALL agent through an FDA-approved drug library screen. Osimertinib monotherapy suppressed leukemic progression in vivo, mediated by proteasome-dependent degradation of c-MYC through a non-canonical, EGFR-independent pathway. More importantly, combining osimertinib with the XPO1 inhibitor selinexor produced a synergistic interaction driven by coordinated disruption of ribosome biogenesis and protein synthesis. This combination was effective at low concentrations across four genetically distinct T-ALL cell lines and retained a favorable safety window in normal cells.

Osimertinib promotes c-MYC degradation through a mechanism that cannot be fully explained by the canonical PI3K/AKT/mTOR and NOTCH1 pathways. Although pharmacological inhibition of PI3K, mTOR, or NOTCH1 alone partially reduced c-MYC, osimertinib produced a greater reduction, and co-treatment with each inhibitor plus osimertinib did not further reduce c-MYC beyond the level achieved by osimertinib alone. In addition, activators of these pathways effectively increased p-PI3K, p-mTOR, or NOTCH1 but did not upregulate c-MYC or rescue osimertinib-induced apoptosis. These data suggest that these pathways are not the primary drivers of c-MYC expression in T-ALL cells and that osimertinib downregulates c-MYC through a mechanism that cannot be fully explained by inhibition of these pathways alone. A cycloheximide (CHX) chase assay confirmed that osimertinib shortened the half-life of c-MYC, and pre-treatment with the proteasome inhibitor MG132 blocked osimertinib-induced c-MYC degradation (Fig. 3L and M; quantification in Supplementary Figures S3H and S3I). Importantly, Z-VAD-FMK did not prevent osimertinib-induced c-MYC degradation (Fig. 3D; Supplementary Figures S2F-S2G), confirming that this process is independent of caspase activation and apoptosis. Together with the lack of EGFR expression and the inability of EGF to rescue apoptosis, these data indicate a non-canonical, EGFR-independent mechanism for osimertinib-induced c-MYC degradation, although the direct molecular target remains to be identified.

The rationale for targeting ribosome biogenesis in T-ALL is supported by the dependence of highly proliferative cancer cells on elevated protein synthesis [32–34]. c-MYC depletion impairs ribosome biogenesis, while selinexor blocks nuclear export of ribosomal subunits. This combined disruption leads to nucleolar stress, manifested as fibrillarin dispersal and accumulation of free ribosomal proteins RPL5 and RPL11. Accumulated RPL5 and RPL11 are associated with decreased MDM2 protein levels, leading to p53 stabilization and phosphorylation, BAX activation, loss of mitochondrial membrane potential, and apoptotic cell death [35–38]. Importantly, BAX activation was confirmed using the 6A7 antibody, and cytochrome c release was detected in the cytoplasmic fraction, providing direct evidence for mitochondrial outer membrane permeabilization. In addition to nucleolar stress, the osimertinib-selinexor combination caused rapid post-transcriptional depletion of c-MYC and suppressed global protein synthesis. In Jurkat cells, osimertinib alone did not clearly reduce c-MYC at 12 h but significantly reduced it at 24 h, whereas MOLT-4 cells responded at 12 h. This suggests that the kinetics of c-MYC reduction vary among T-ALL cell lines, possibly reflecting differences in c-MYC half-life, protein turnover, or *TP53* status. Notably, the combination of osimertinib and selinexor consistently reduced c-MYC across all four cell lines.

Nucleolar stress and translational suppression are mechanistically distinct but functionally complementary. They identify c-MYC as a critical integrator of proliferative signals and cellular stress responses [39–41]. This dual mechanism may explain the superior efficacy observed with the combination compared with single-agent treatments.

Our study also points to candidate biomarkers for future evaluation. Baseline c-MYC expression/dependency and *TP53* status may serve as candidate stratification factors, whereas treatment-induced c-MYC depletion and RPL5/RPL11 nuclear accumulation may serve as candidate pharmacodynamic biomarkers. The four T-ALL cell lines have different *TP53* backgrounds: MOLT-3 is wild-type; Jurkat carries a truncating R196* mutation; CCRF/CEM carries R175H and R248Q missense mutations; and MOLT-4 carries R248Q and R306* mutations. The differential responses among these lines suggest that *TP53* status and mutation type may influence sensitivity, but *TP53* status alone is unlikely to predict response. These candidate biomarkers are hypothesis-generating and require validation in larger patient cohorts and functional studies.

The clinical potential of this combination is supported by the established clinical pharmacology and characterized toxicity profiles of both agents. Selinexor is FDA-approved for multiple myeloma and diffuse large B-cell lymphoma, while osimertinib is standard-of-care for EGFR-mutant non-small cell lung cancer [42, 43]. Although single-agent selinexor has shown limited efficacy in T-ALL [44, 45], our data suggest that combining it with osimertinib enhances apoptosis at low doses. This raises the possibility of dose reduction, which could mitigate the hematologic and gastrointestinal toxicities associated with higher selinexor doses [46, 47]. Consistently, the combination at 2 µM osimertinib plus 20 nM selinexor, which showed clear synergy in all four T-ALL cell lines, had minimal cytotoxicity in normal human bone marrow mononuclear cells and HEK293T cells (Supplementary Figures S5), indicating a favorable safety window. The clinical pharmacokinetics of osimertinib are well characterized. At the standard dose of 80 mg once daily, the steady-state Cmax is approximately 694 nM (0.69 µM), and the trough concentration is approximately 438–459 nM (0.44–0.46 µM), defining a clinically relevant plasma concentration range of approximately 0.4-1 µM [48, 49]. In vitro single-agent experiments, osimertinib was used at 5–10 µM, which is higher than clinically achievable plasma levels. To address this gap, we tested lower concentrations of osimertinib (0.5, 1, and 2 µM) alone and in combination with selinexor (20 nM). Although osimertinib alone at these concentrations did not induce significant apoptosis, the combination significantly increased apoptosis compared with either single agent in several cell lines. At 1 µM, the combination significantly exceeded both single agents in three of four T-ALL cell lines (MOLT-4, MOLT-3, and CCRF/CEM), suggesting that clinically achievable concentrations may provide benefit when combined with selinexor. In Jurkat cells, the combination significantly increased apoptosis compared with osimertinib alone but did not significantly exceed selinexor alone. However, the most pronounced synergy was observed at 2 µM, which exceeds the clinical Cmax (~ 1 µM). Tissue distribution studies using PBPK modeling and PET imaging have shown that osimertinib achieves tumor-to-blood ratios > 1 in lung tumors [50], but direct measurements in T-ALL patients are not available. Therefore, further studies with pharmacokinetic monitoring and PDX models are needed to determine whether clinically achievable concentrations produce similar anti-leukemic effects in vivo.

Several limitations of this study warrant consideration. First, the direct molecular target of osimertinib in T-ALL cells remains unidentified. While we ruled out canonical EGFR signaling and confirmed proteasome-dependent c-MYC degradation, the precise protein(s) to which osimertinib binds to trigger this effect require further investigation using chemical proteomics or kinome-wide profiling. Second, the upstream E3 ubiquitin ligase responsible for c-MYC degradation upon osimertinib treatment is not defined; candidates such as FBXW7, TRIM21, and USP28 should be systematically examined in future studies [51–53]. Third, although we expanded primary T-ALL blast analysis from two to four patients (Fig. 1G), the limited cell numbers precluded additional mechanistic experiments such as immunoblotting on primary blasts. Larger cohorts of primary T-ALL samples are therefore needed to confirm the generalizability of our findings. Fourth, our in vivo efficacy data are derived exclusively from the MOLT-4 xenograft model; validation in patient-derived xenografts (PDX) representing the genetic heterogeneity of T-ALL (e.g., NOTCH1, FBXW7, PHF6 mutations) would substantially strengthen clinical translatability [54, 55]. Due to limited primary sample availability (only two additional patients were admitted during the revision period, and the original two samples had no remaining viable cells) and the time required for PDX establishment, PDX experiments were not completed in this study; this remains a major limitation. Future multi-center collaborations and PDX models are warranted to confirm clinical translatability. Fifth, overlapping toxicities of these agents (gastrointestinal and hematologic) necessitate careful dose optimization in future clinical trials. Sixth, the relatively high osimertinib concentrations used in CETSA and DARTS were selected to maximize assay sensitivity and target saturation [56–58]. However, these concentrations exceed clinically relevant cellular exposures; therefore, these biochemical findings should be interpreted cautiously and require orthogonal validation at lower concentrations. Seventh, although 1 µM osimertinib combined with selinexor showed synergy in three of four T-ALL cell lines, the most pronounced synergy required 2 µM, which exceeds the clinical Cmax (~ 1 µM). In vivo studies did not include pharmacokinetic monitoring to confirm that clinically achievable concentrations were achieved. Therefore, further studies are needed to determine whether clinically achievable concentrations produce similar anti-leukemic effects in vivo. Eighth, our in vivo studies were performed in NSG mice, which lack an intact immune system. Therefore, the impact of this combination on the immune microenvironment and immune-mediated anti-leukemic effects could not be evaluated. Future studies using immunocompetent or humanized mouse models are warranted to address this issue. Ninth, although the combination significantly reduced bone marrow leukemic burden at day 21, residual extramedullary disease may persist and contribute to subsequent mortality. This is consistent with the observation that survival was monitored in an independent cohort until humane endpoints. Tenth, the kinetics of c-MYC protein reduction differed among cell lines (12 h in MOLT-4 vs. 24 h in Jurkat, CCRF/CEM, and MOLT-3), and c-MYC half-life was not directly measured; this may reflect cell line-specific differences in c-MYC stability or turnover. Finally, resistance mechanisms and long-term efficacy were not evaluated in this study. Despite these caveats, the FDA-approved status of both drugs and the availability of extensive pharmacokinetic and safety data support future clinical evaluation of this combination in patients with relapsed/refractory T-ALL [59, 60].

**Fig. 7:**
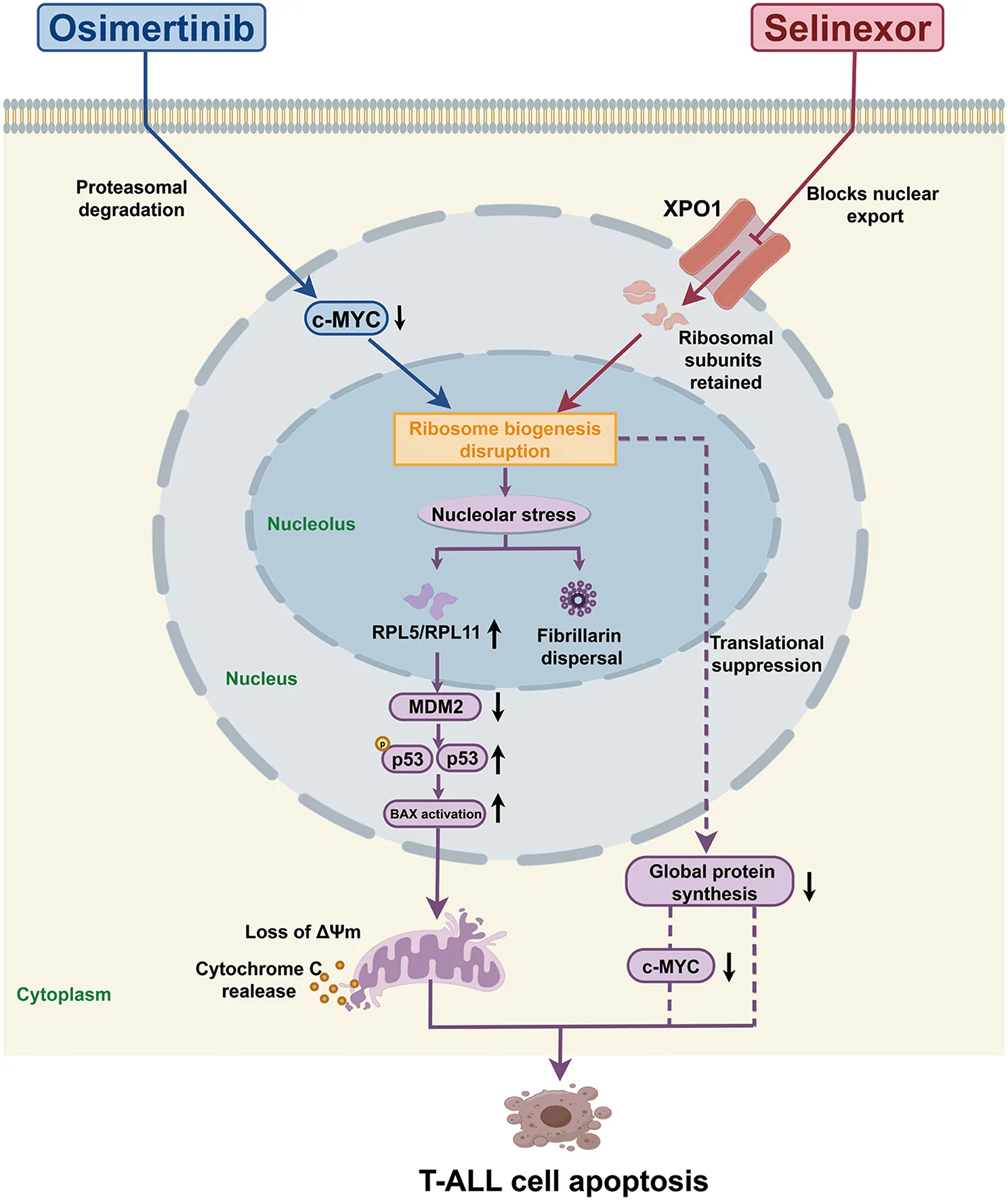
Synergistic mechanism of osimertinib and selinexor in T-ALL. Osimertinib promotes proteasomal degradation of c-MYC through a non-canonical, EGFR-independent pathway, leading to impaired ribosome biogenesis. Selinexor inhibits XPO1, blocking nuclear export of ribosomal subunits. The combination cooperatively disrupts ribosome biogenesis, triggering nucleolar stress characterized by fibrillarin dispersal and nuclear accumulation of free ribosomal proteins RPL5 and RPL11. Accumulated RPL5 and RPL11 are associated with decreased MDM2 protein levels, leading to p53 stabilization and phosphorylation, BAX activation (detected by the 6A7 antibody), cytochrome c release, loss of mitochondrial membrane potential (ΔΨm), and apoptotic cell death. Concurrently, the combination causes rapid post‑transcriptional depletion of c-MYC and suppression of global protein synthesis, cooperatively driving T-ALL cell apoptosis. Solid arrows indicate proposed pathway relationships; dashed arrows indicate indirect or convergent effects. (Created with Figdraw.)

In summary, osimertinib and selinexor impair ribosome biogenesis, triggering nucleolar stress, while concurrently depleting c-MYC and suppressing global protein synthesis. These dual downstream events promote T-ALL cell apoptosis (Fig. 7). This mechanism-driven combination represents a preclinical strategy for T-ALL and further studies incorporating pharmacokinetic monitoring, PDX models, and prospective biomarker validation are warranted before clinical translation.

## Supplementary Information

Below is the link to the electronic supplementary material.

Supplementary Material 1

Supplementary Material 2

Supplementary Material 3

Supplementary Material 4

## Data Availability

RNA Sequencing datasets are deposited in the NCBI Sequence Read Archive (PRJNA1474603). Proteomic data are deposited in the ProteomeXchange (PXD085339). All data analyzed in this study are available from the corresponding author on reasonable request.

## Abbreviations

ATCC: American Type Culture Collection
APC: Allophycocyanin
BAX: BCL2-associated X protein
BCA: Bicinchoninic acid
BMMNCs: Bone marrow mononuclear cells
CETSA: Cellular thermal shift assay
CHX: Cycloheximide
c-MYC: MYC proto-oncogene, bHLH transcription factor
DAB: Diaminobenzidine
DAPI: 4’,6-diamidino-2-phenylindole
DARTS: Drug affinity responsive target stability
DMSO: Dimethyl sulfoxide
EGF: Epidermal growth factor
EGFR: Epidermal growth factor receptor
FBL: Fibrillarin
FBS: Fetal bovine serum
FDA: Food and drug administration
Fer-1: Ferrostatin-1
GAPDH: Glyceraldehyde-3-phosphate dehydrogenase
GSEA: Gene set enrichment analysis
H&E: Histologic examination
hCD45: Human CD45
HPA: Human protein atlas
HRP: Horseradish peroxidase
HSA: Highest single agent
IC_50_: Half-maximal inhibitory concentration
IF: Immunofluorescence
IHC: Immunohistochemistry
KEGG: Kyoto encyclopedia of genes and genomes
MDM2: Mouse double minute 2 homolog
MFI: Mean fluorescence intensity
MOMP: Mitochondrial outer membrane permeabilization
Nec-1: Necrostatin-1
NSG: NOD scid gamma
nTPM: Normalized transcripts per million
OD: Optical density
PCR: Polymerase chain reaction
PDX: Patient-derived xenografts
PI: Propidium iodide
PVDF: Polyvinylidene difluoride
RIPA: Radioimmunoprecipitation assay (buffer)
RPL5: Ribosomal protein L5
RPL11: Ribosomal protein L11
RPMI: Roswell Park Memorial Institute
RPS6: Ribosomal protein S6
RT-qPCR: Reverse transcription quantitative polymerase chain reaction
SD: Standard deviation
SDS-PAGE: Sodium dodecyl sulfate–polyacrylamide gel electrophoresis
SPF: Specific pathogen-free
STR: Short tandem repeat
TMRE: Tetramethylrhodamine ethyl ester
TP53: Tumor protein p53
T-ALL: T-cell Acute Lymphoblastic Leukemia
WBC: White blood cell
XPO1: Exportin 1
