# Supplementary material for: Synergistic efficacy of the EGFR inhibitor osimertinib with the XPO1 inhibitor selinexor in T-cell acute lymphoblastic leukemia
Source: Biomark Res. 2026 Oct 28;14:152. doi: 10.1186/s40364-026-01031-w (PMC13718067; doi:10.1186/s40364-026-01031-w)

Uncropped original immunoblot images for the manuscript

Figure 1 Osimertinib induces apoptosis in T-ALL cell lines and primary patient samples.

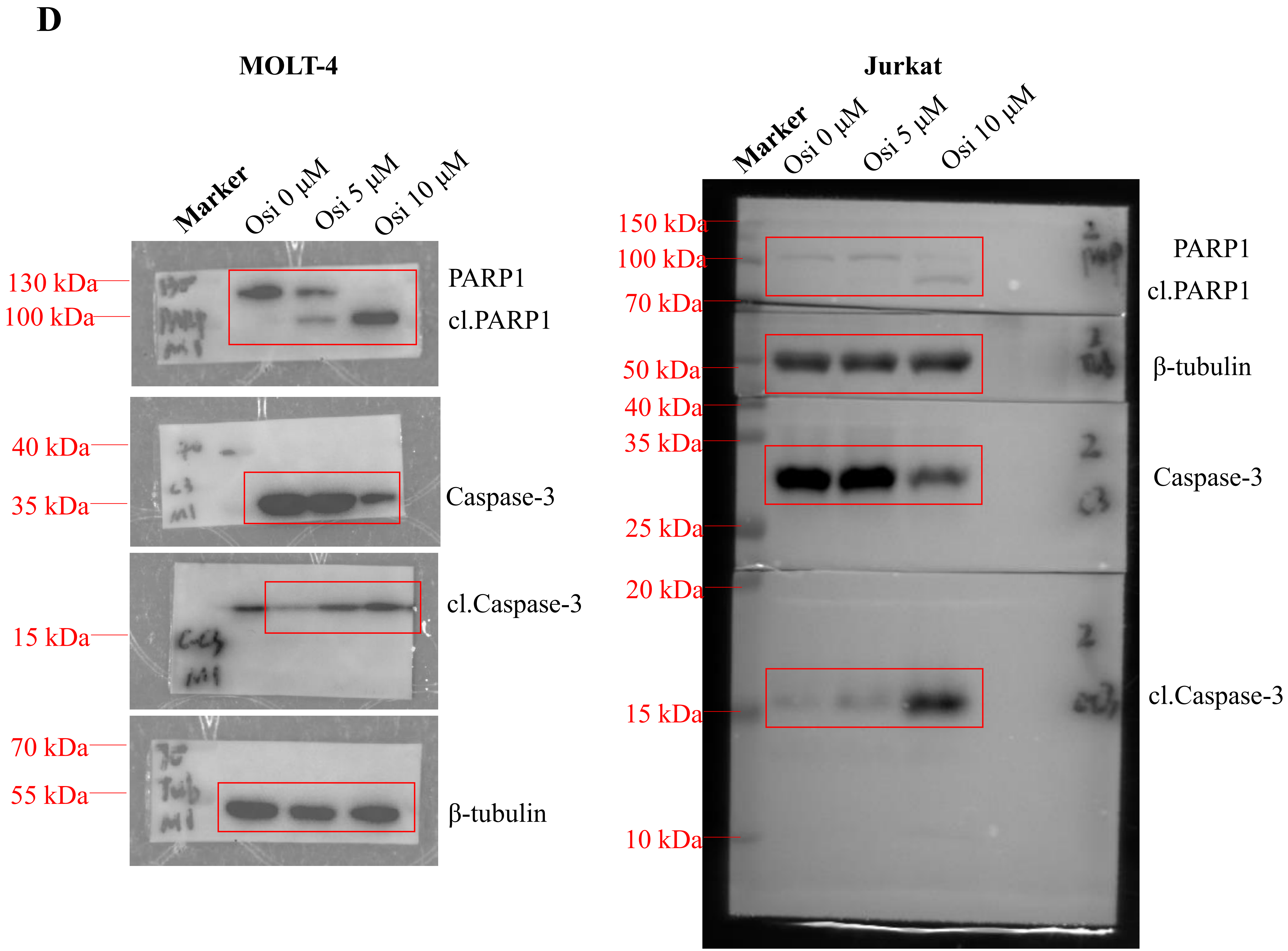

Figure 3 Osimertinib induces c-MYC destabilization through a non-canonical, EGFR-independent mechanism.

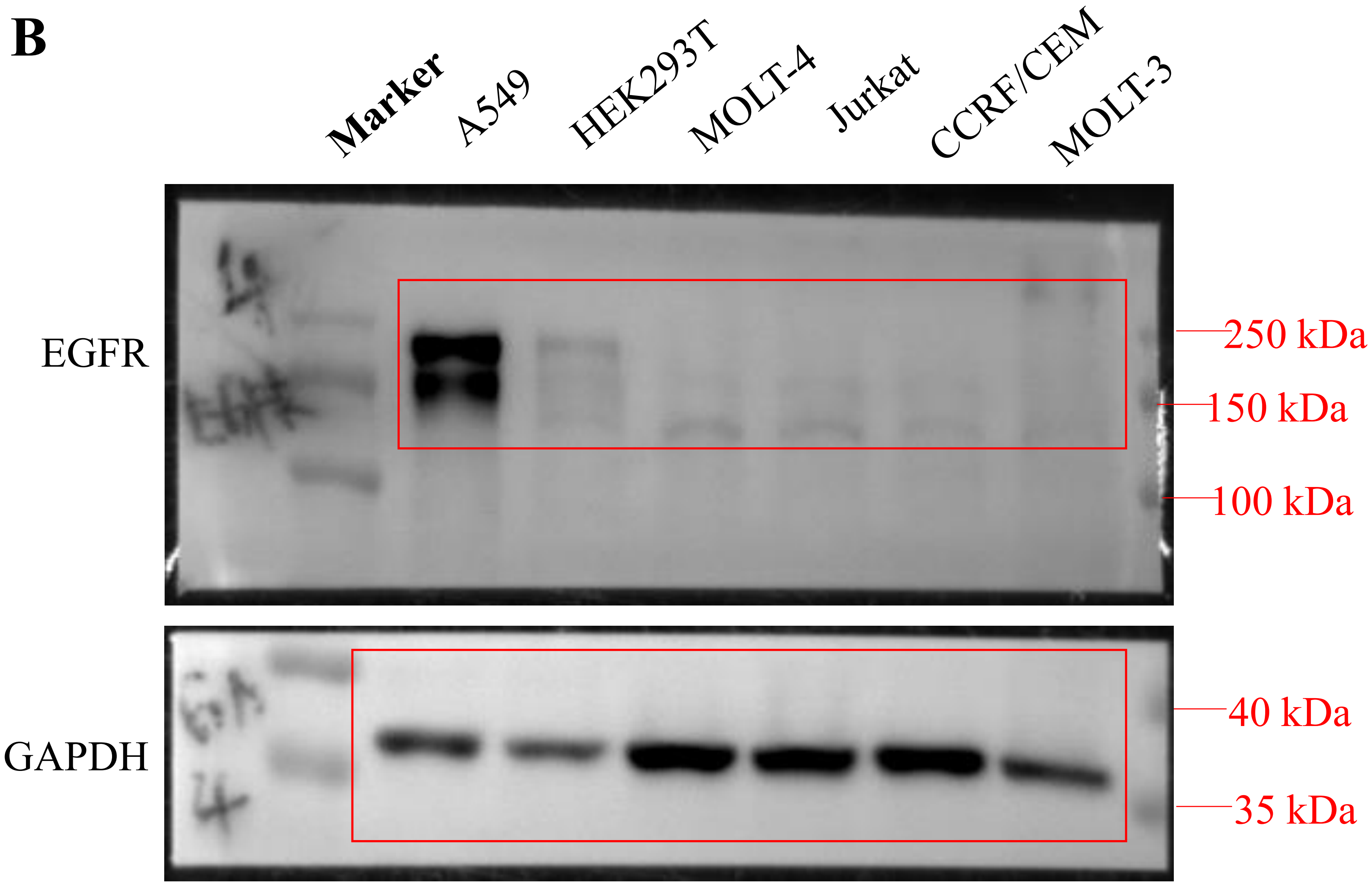

Figure 3 Osimertinib induces c-MYC destabilization through a non-canonical, EGFR-independent mechanism.

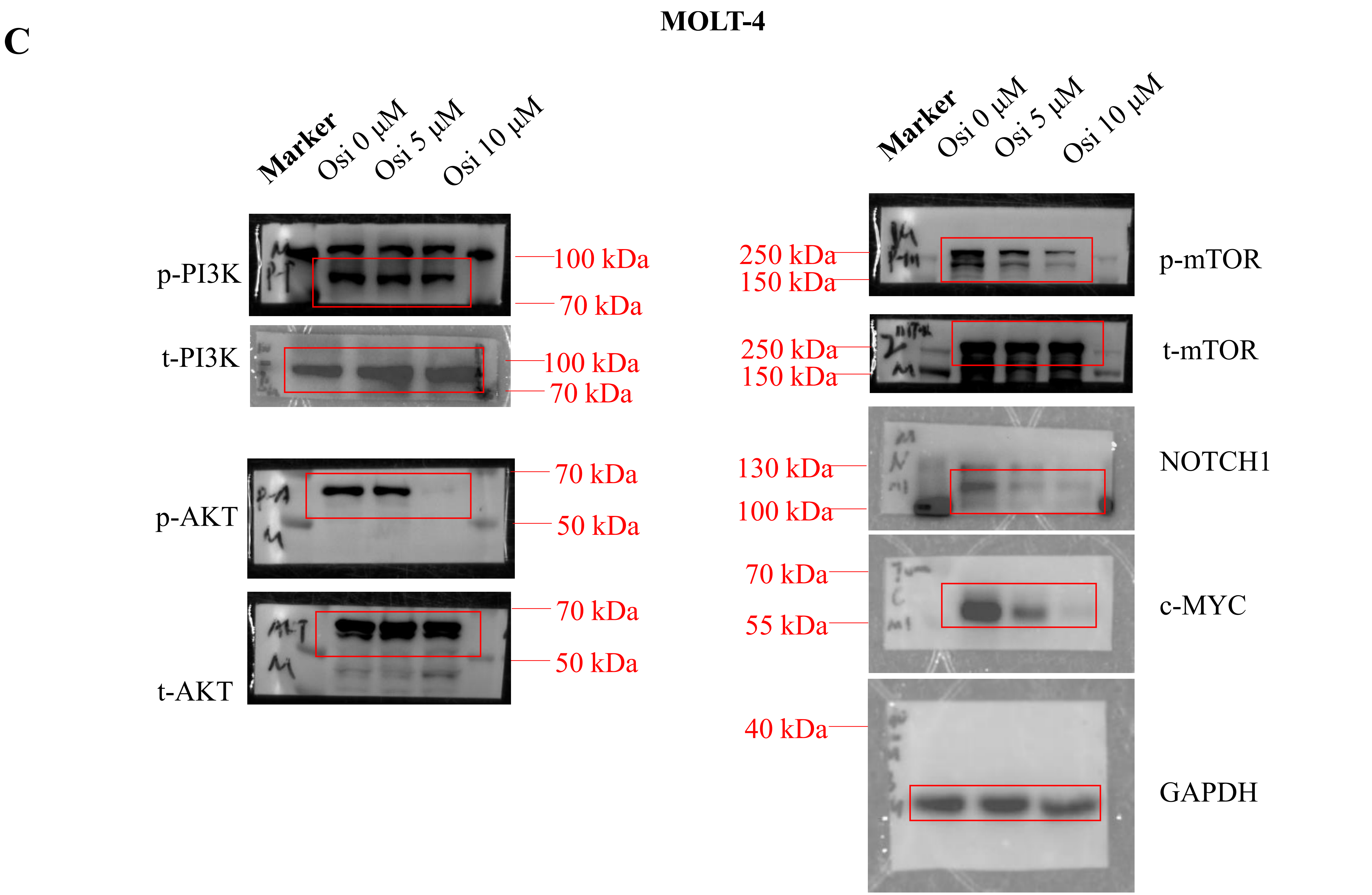

Figure 3 Osimertinib induces c-MYC destabilization through a non-canonical, EGFR-independent mechanism.

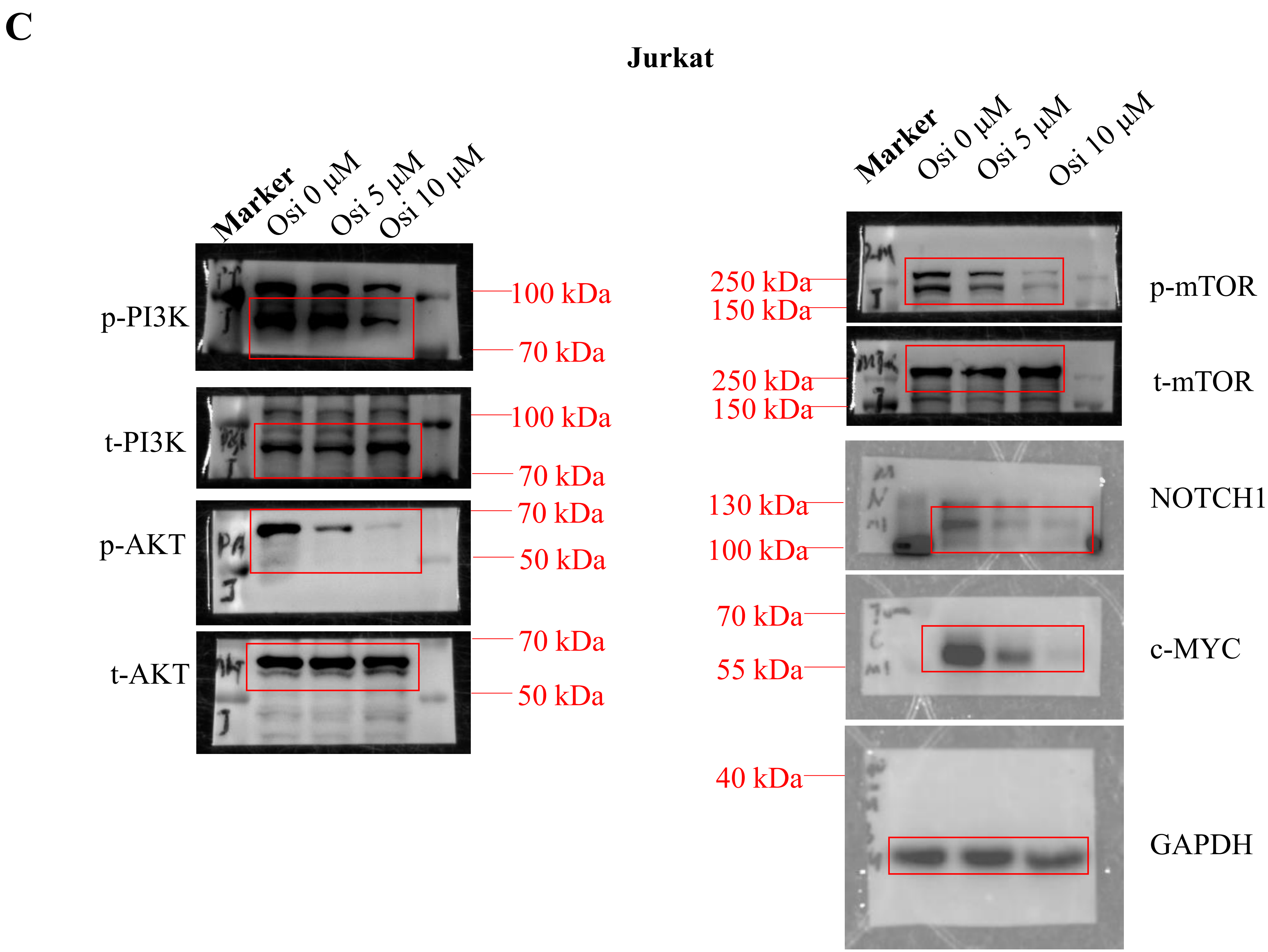

Figure 3 Osimertinib induces c-MYC destabilization through a non-canonical, EGFR-independent mechanism.

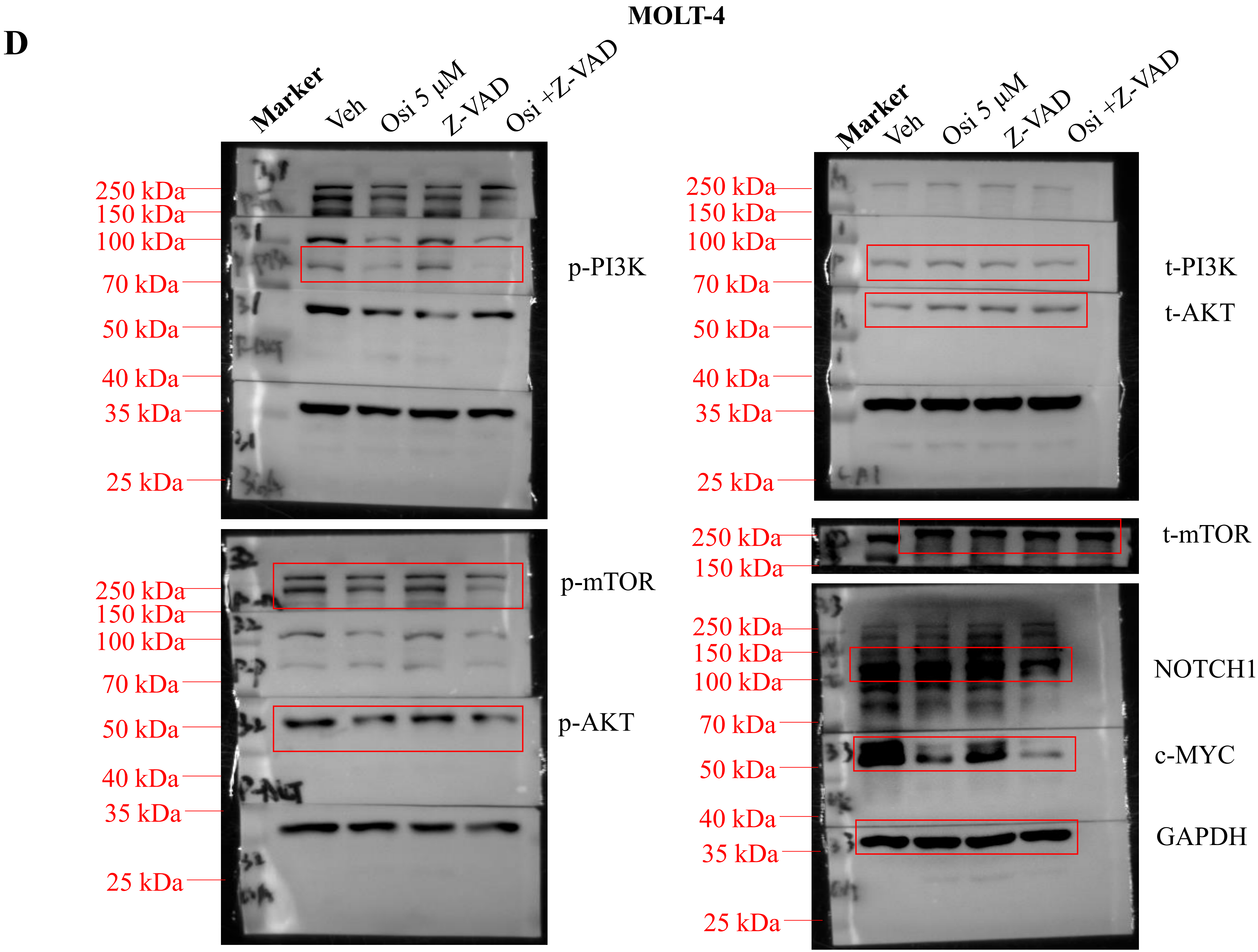

Figure 3 Osimertinib induces c-MYC destabilization through a non-canonical, EGFR-independent mechanism.

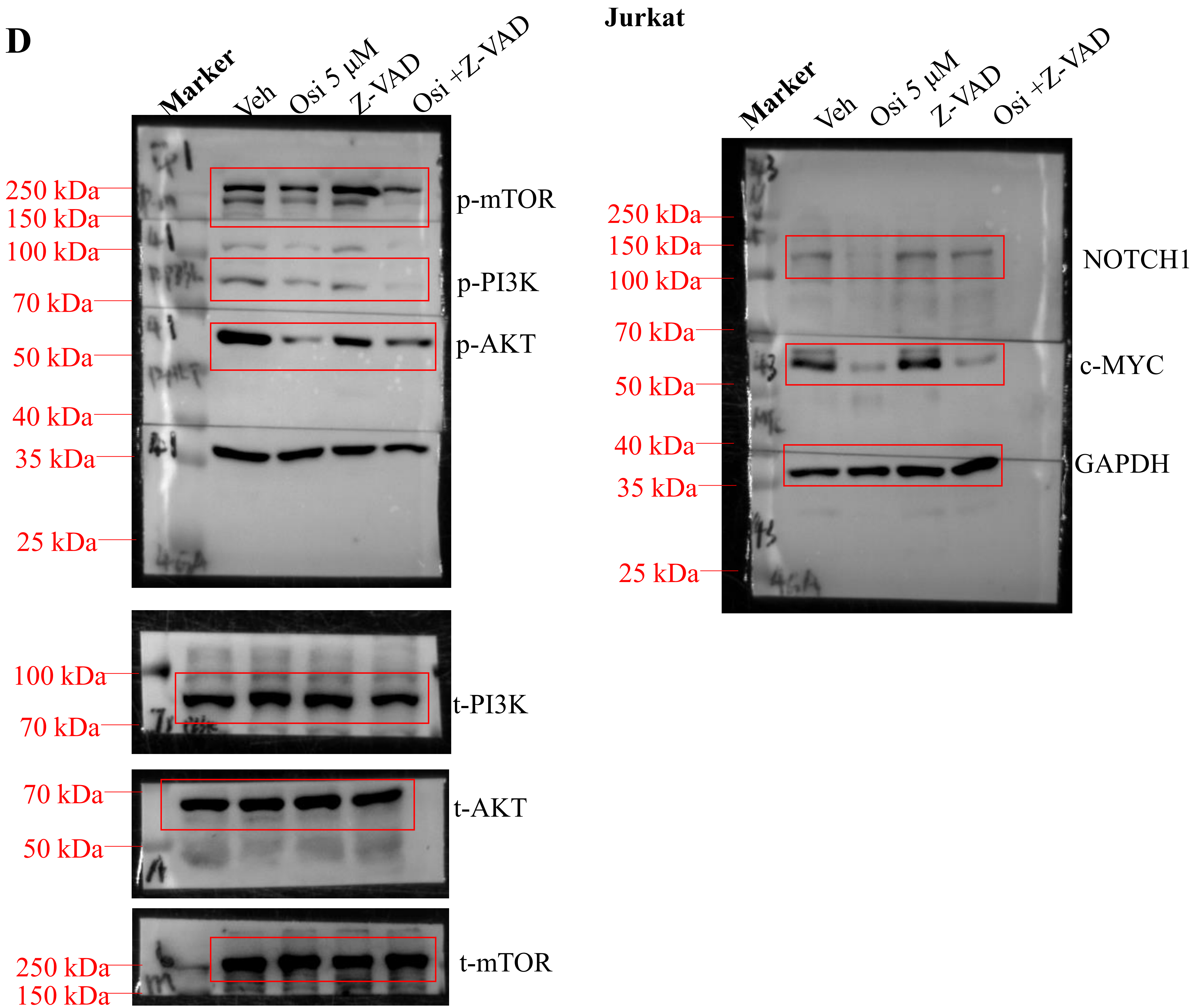

Figure 3 Osimertinib induces c-MYC destabilization through a non-canonical, EGFR-independent mechanism.

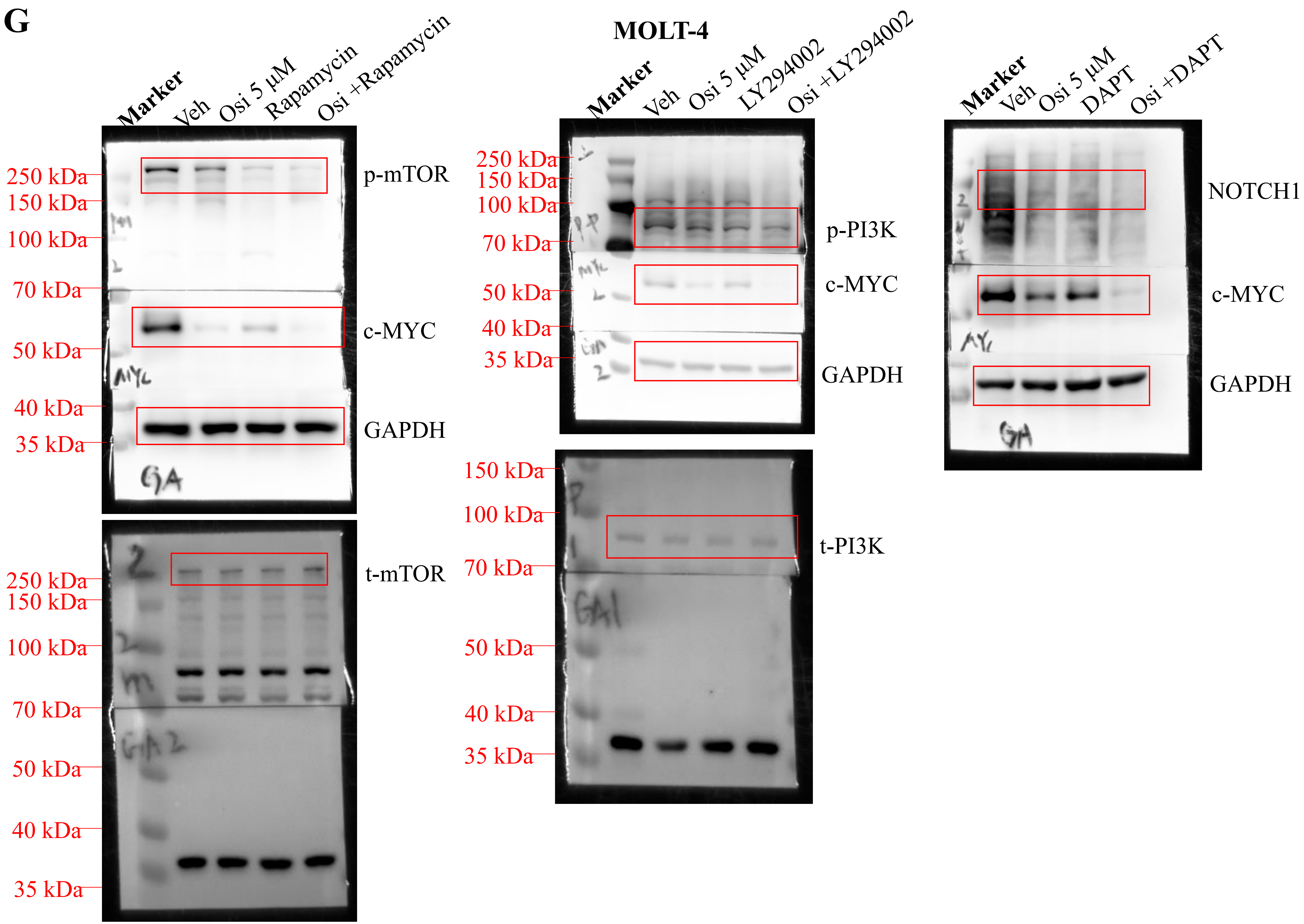

Figure 3 Osimertinib induces c-MYC destabilization through a non-canonical, EGFR-independent mechanism.

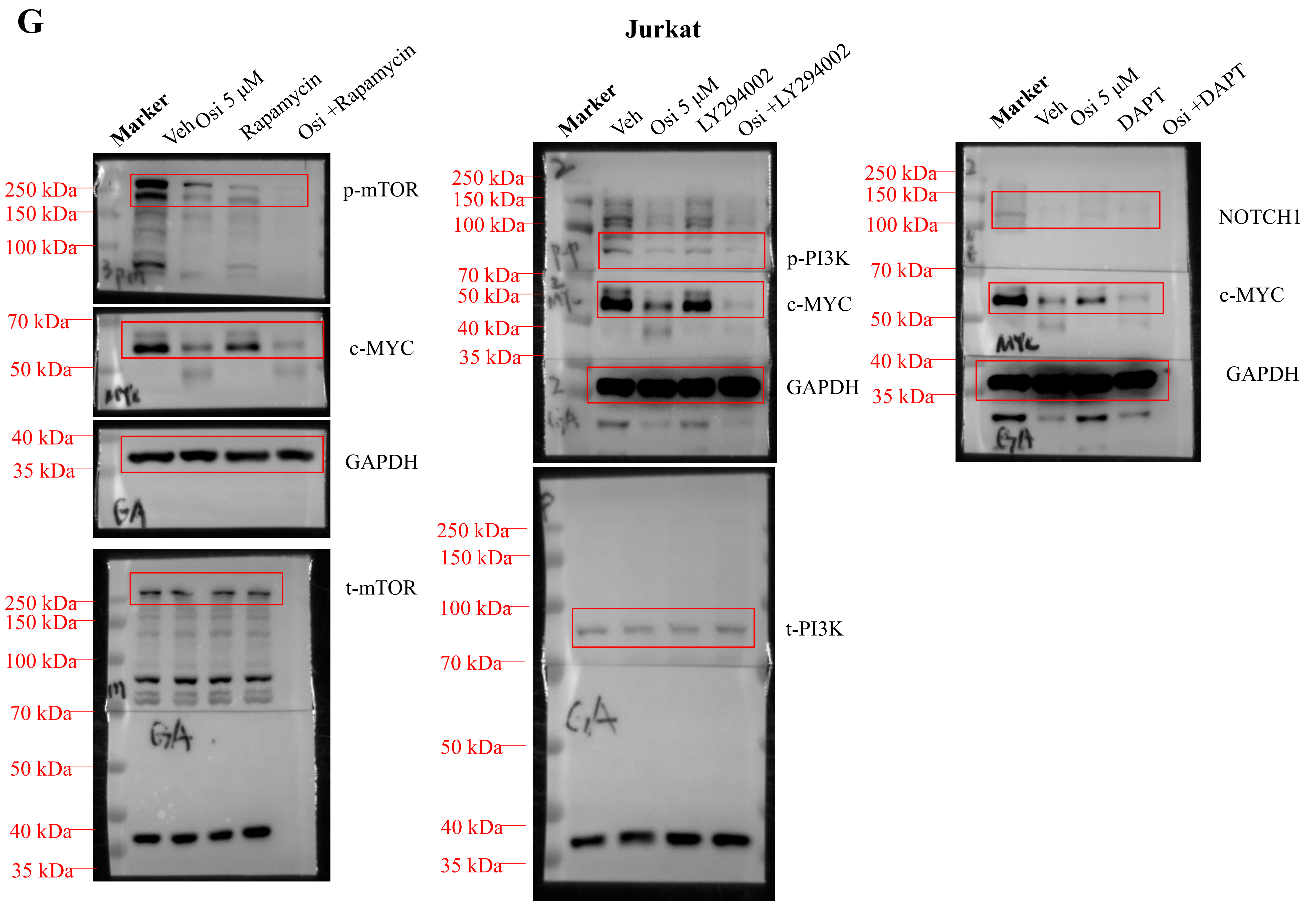

Figure 3 Osimertinib induces c-MYC destabilization through a non-canonical, EGFR-independent mechanism.

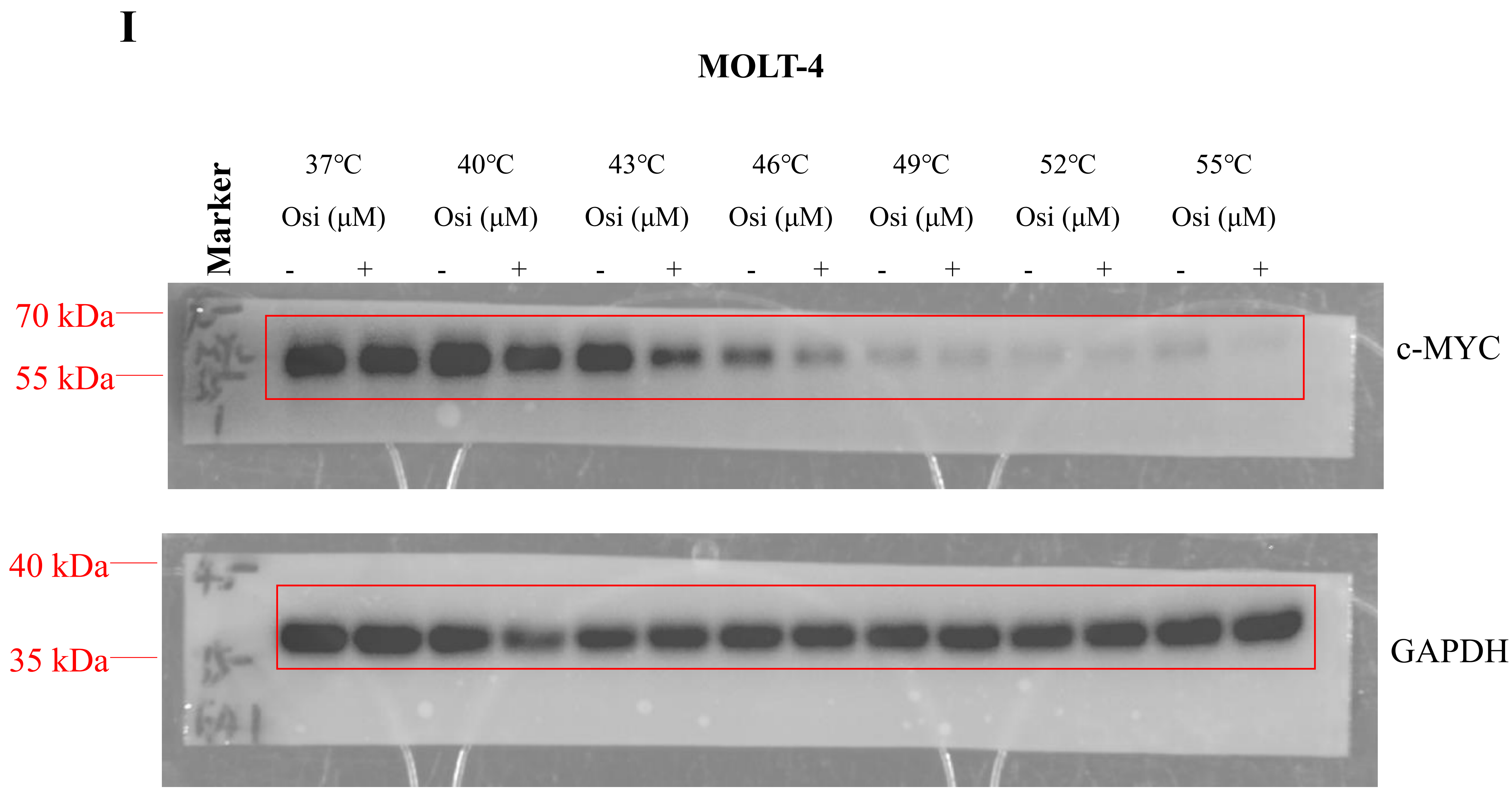

Figure 3 Osimertinib induces c-MYC destabilization through a non-canonical, EGFR-independent mechanism.

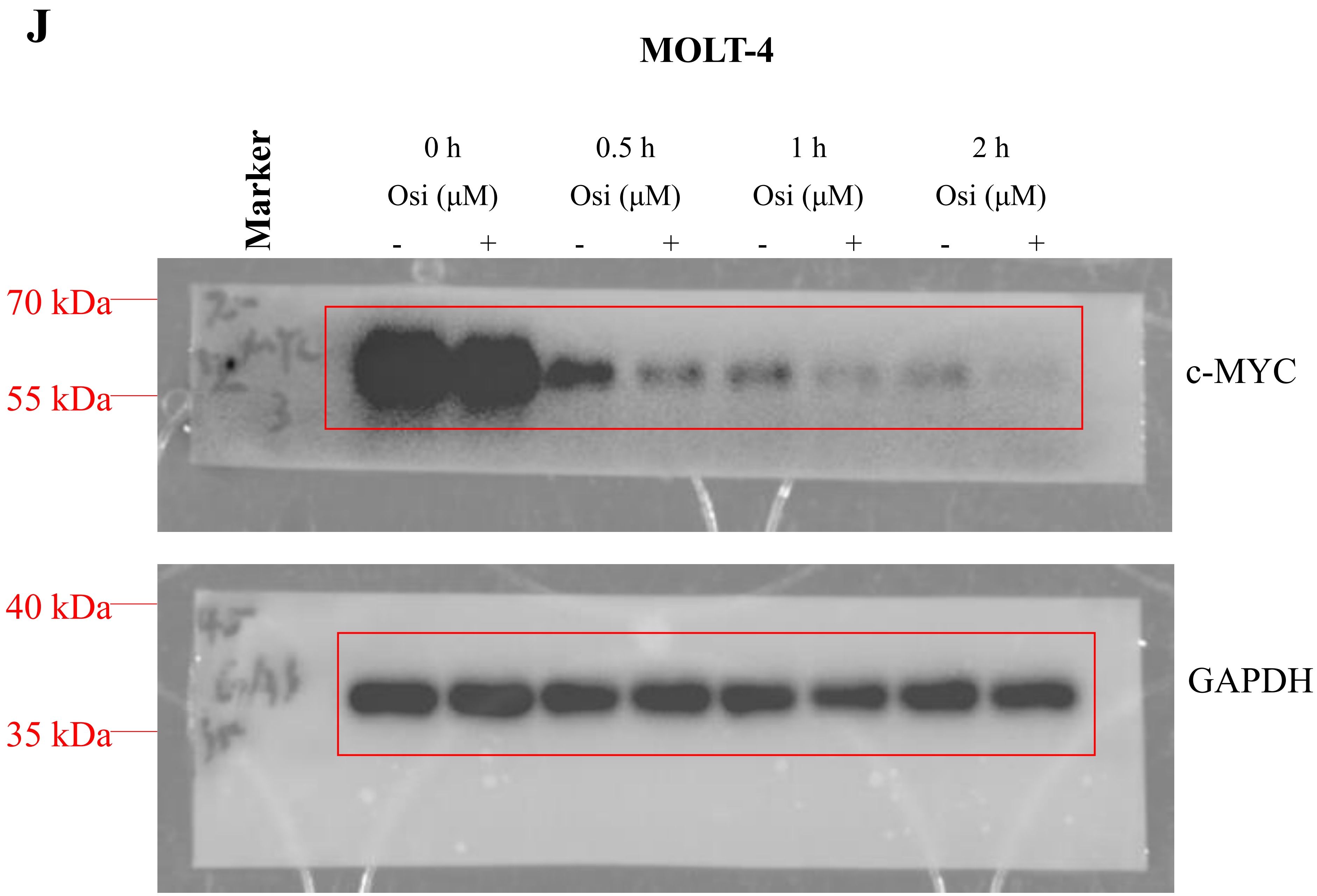

Figure 3 Osimertinib induces c-MYC destabilization through a non-canonical, EGFR-independent mechanism.

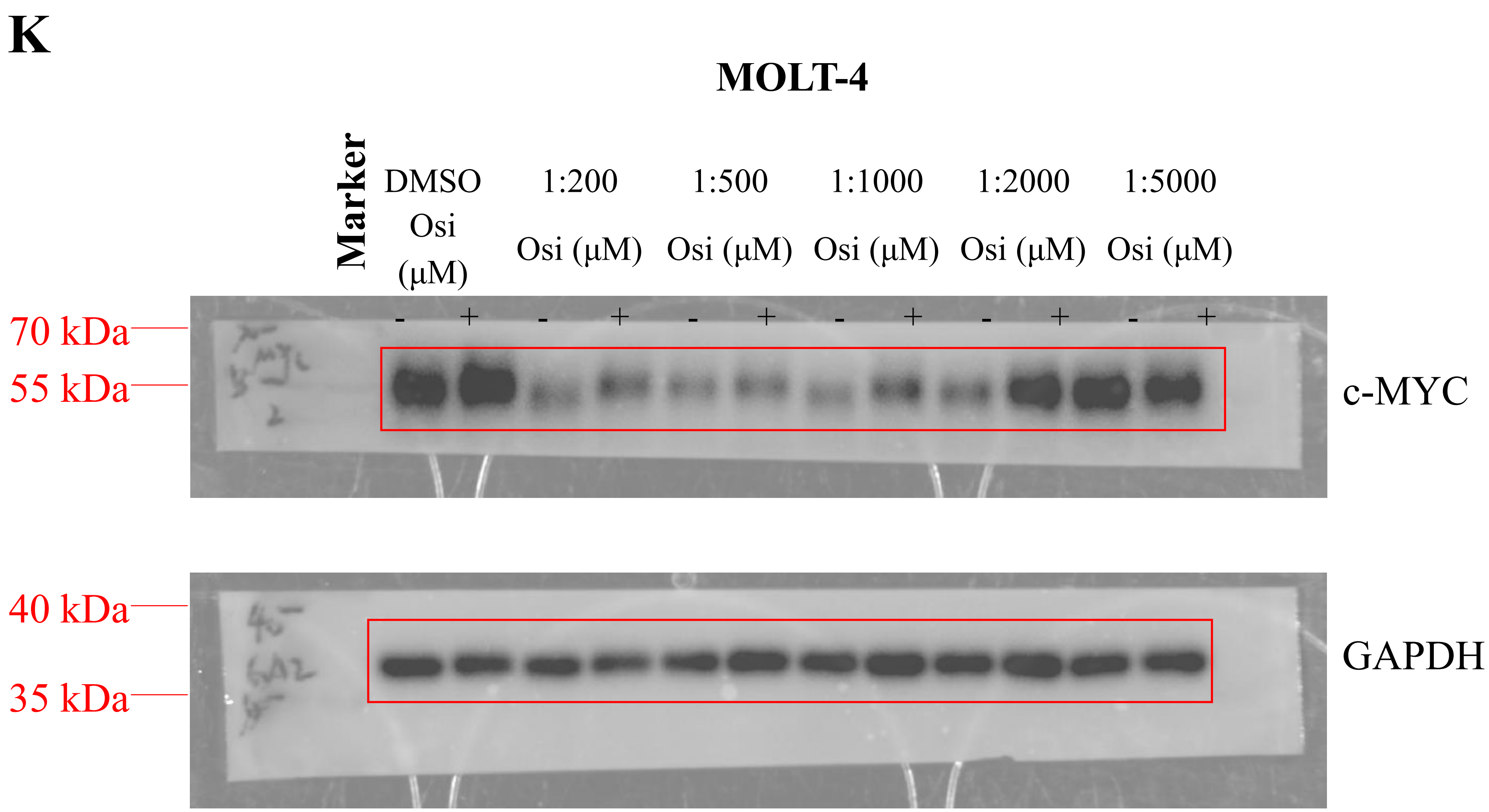

Figure 3 Osimertinib induces c-MYC destabilization through a non-canonical, EGFR-independent mechanism.

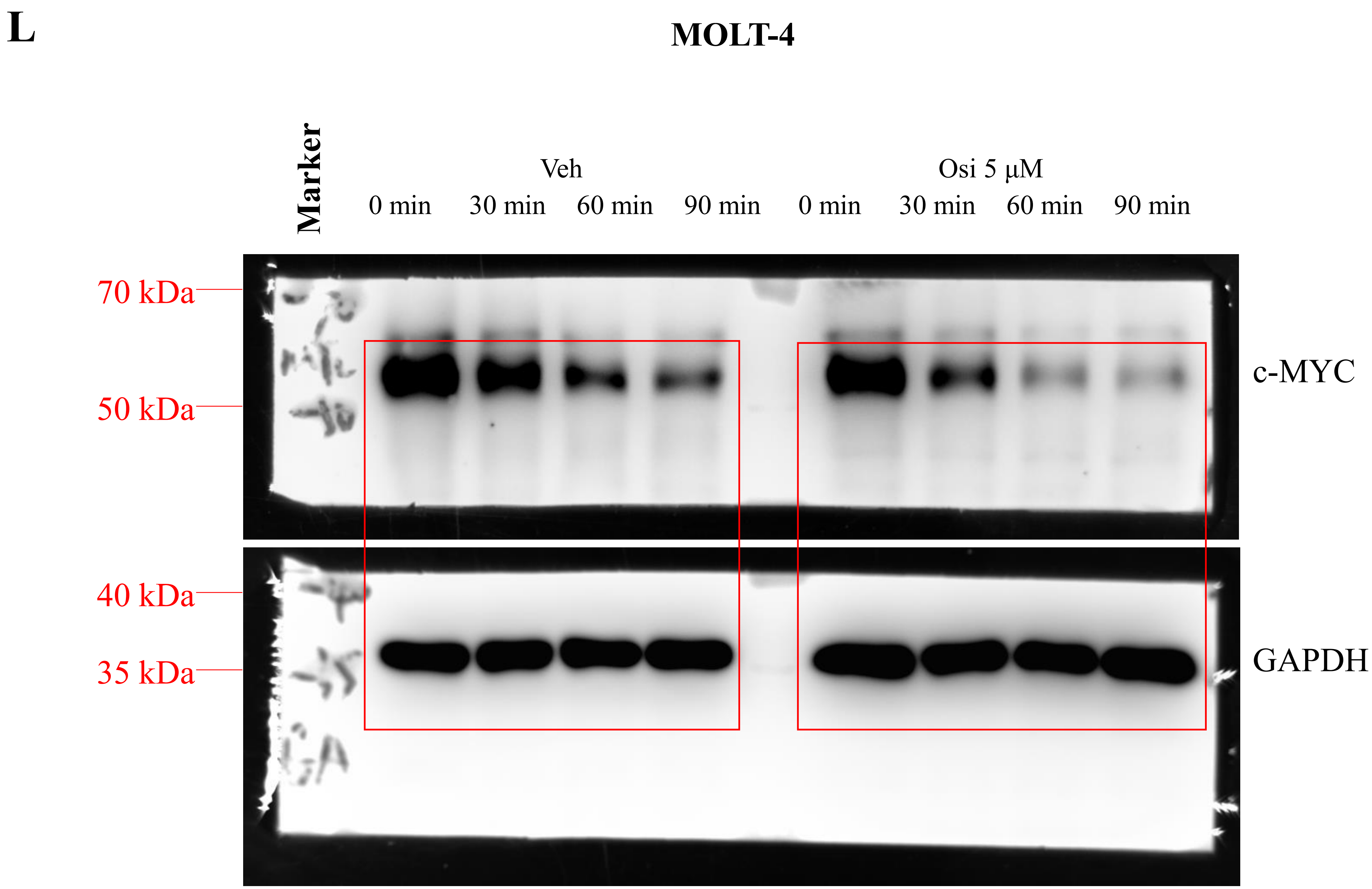

Figure 3 Osimertinib induces c-MYC destabilization through a non-canonical, EGFR-independent mechanism.

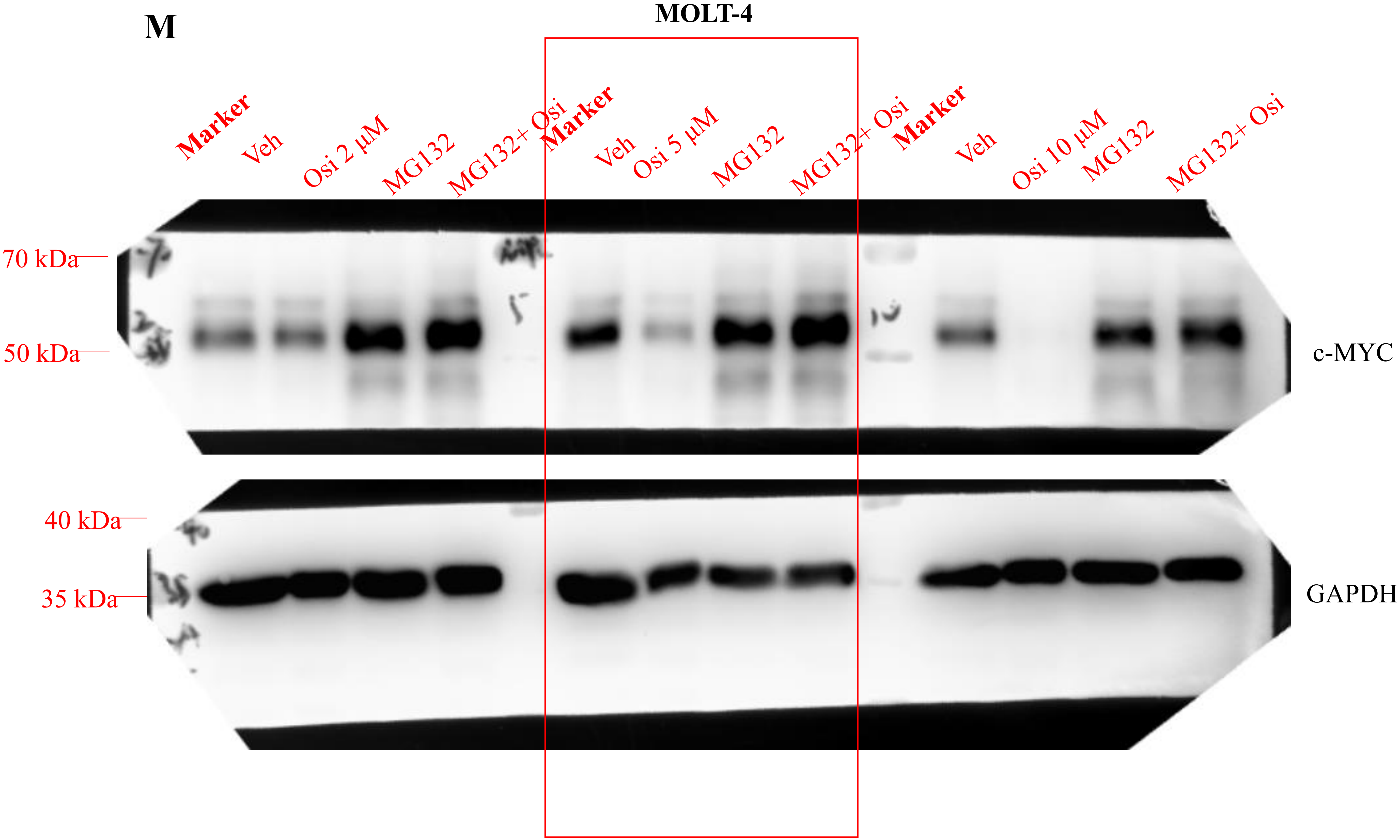

Figure 4 Cotargeting ribosomal biogenesis synergizes with selinexor.

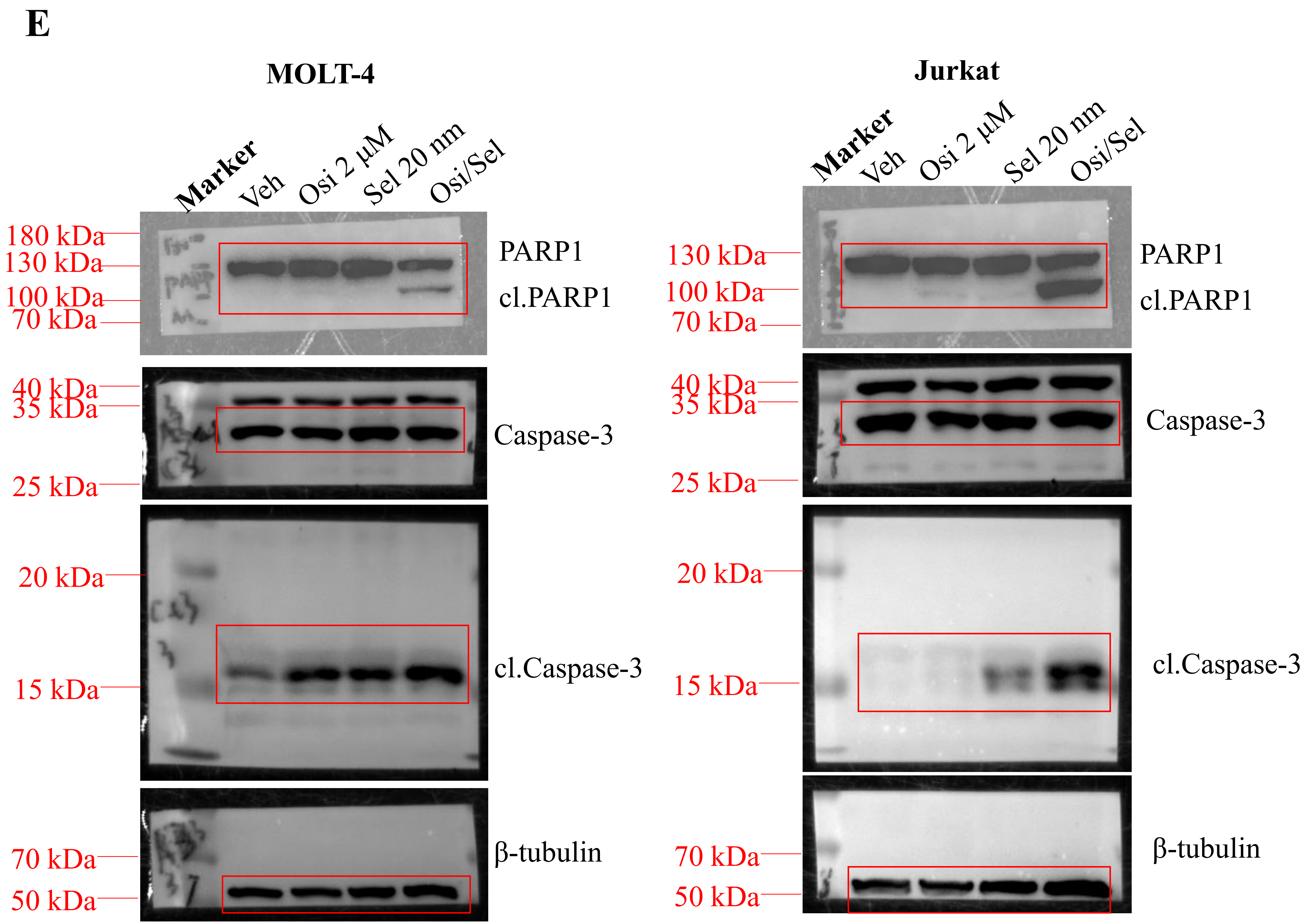

Figure 6 The selinexor-osimertinib combination induces nucleolar stress, translational suppression, and c-MYC depletion.

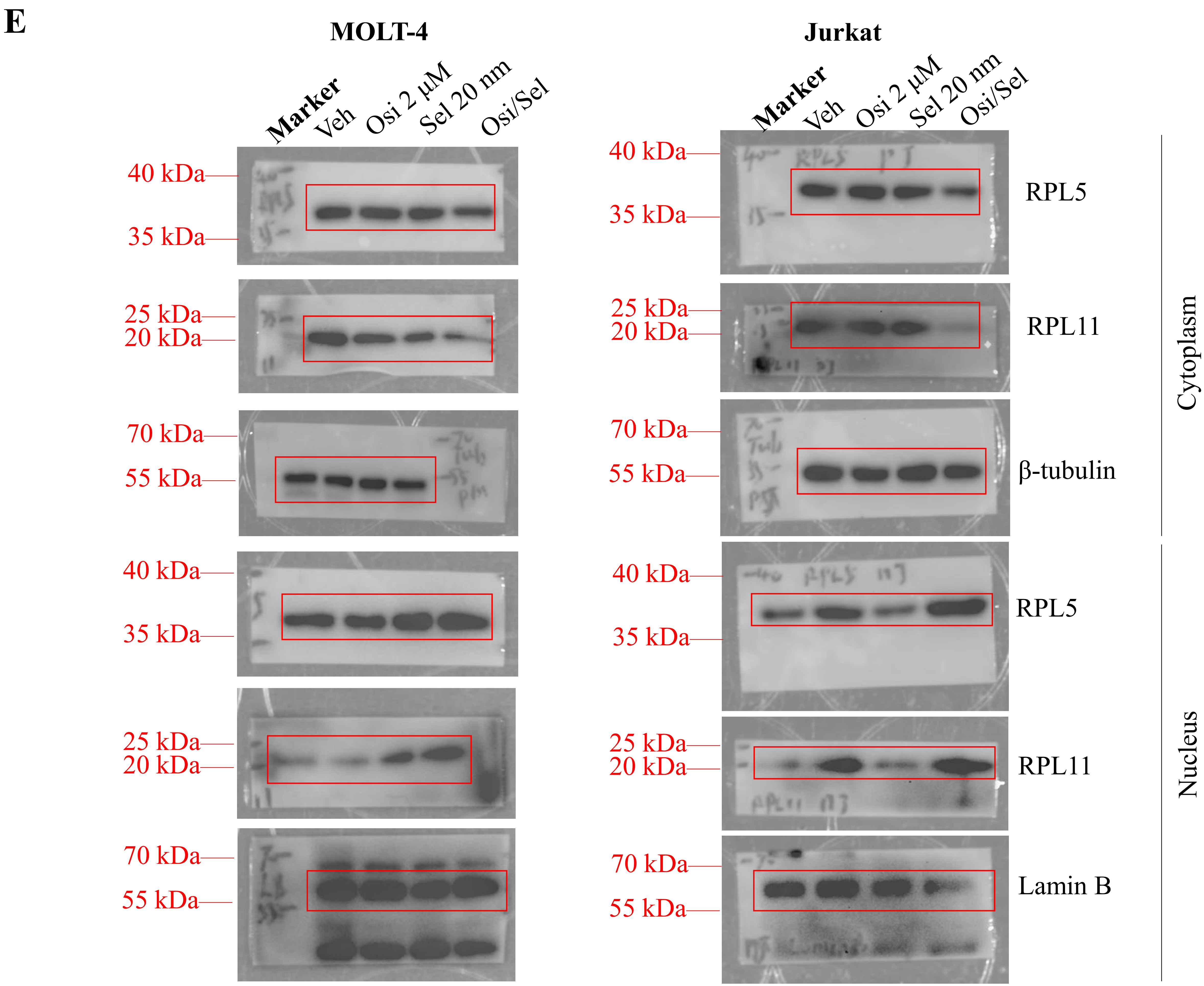

Figure 6 The selinexor-osimertinib combination induces nucleolar stress, translational suppression, and c-MYC depletion.

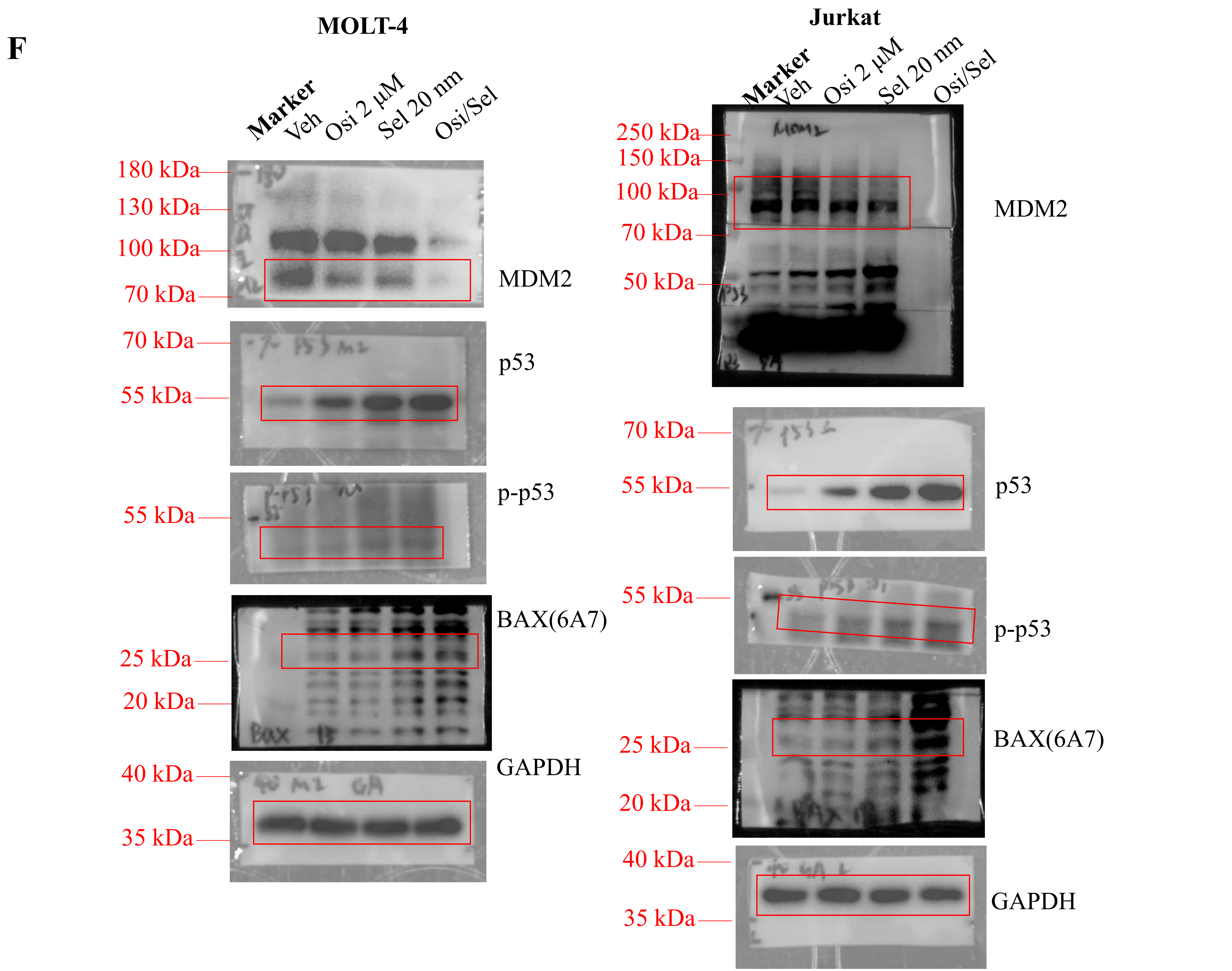

Figure 6 The selinexor-osimertinib combination induces nucleolar stress, translational suppression, and c-MYC depletion.

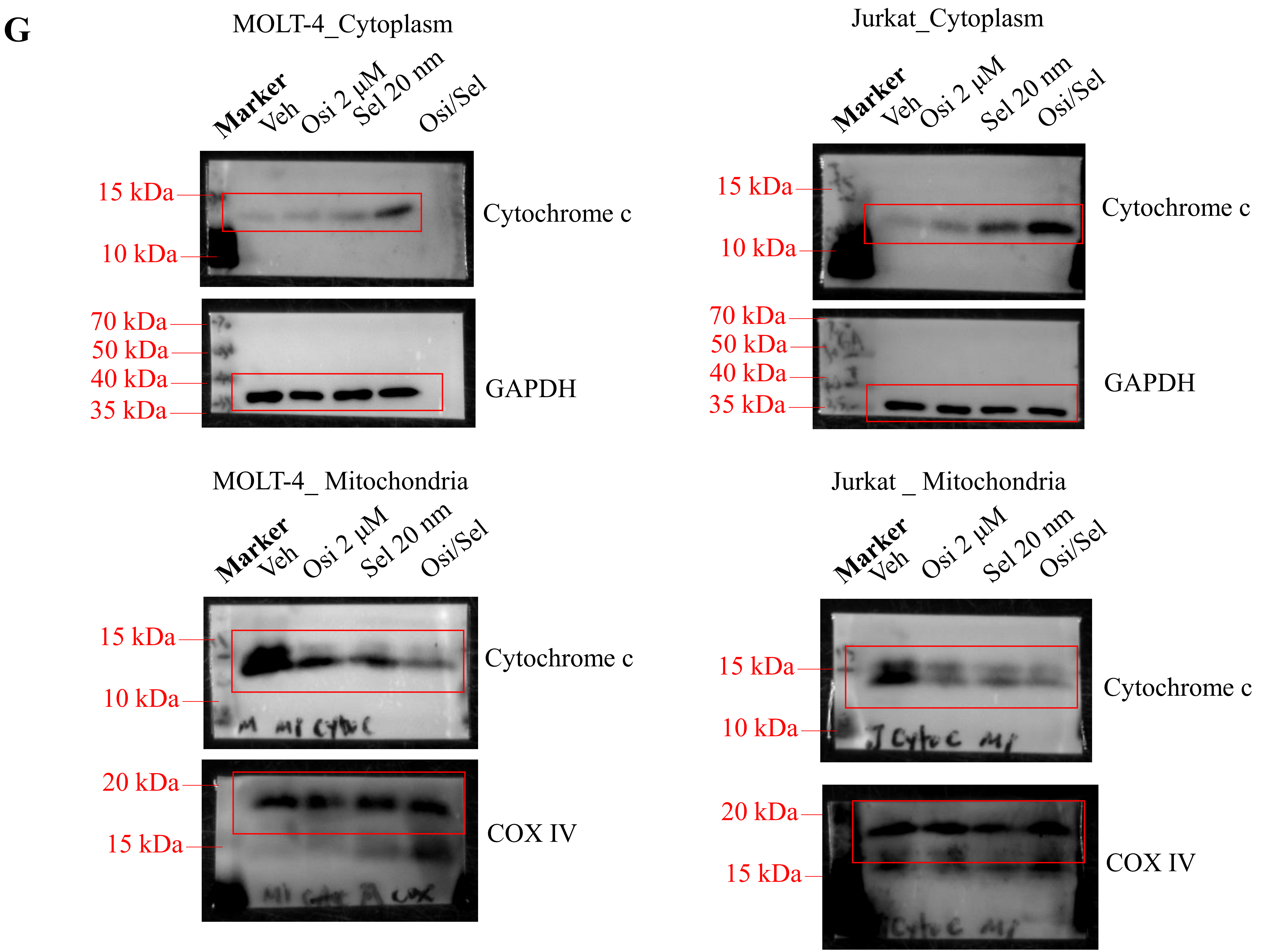

Figure 6 The selinexor-osimertinib combination induces nucleolar stress, translational suppression, and c-MYC depletion.

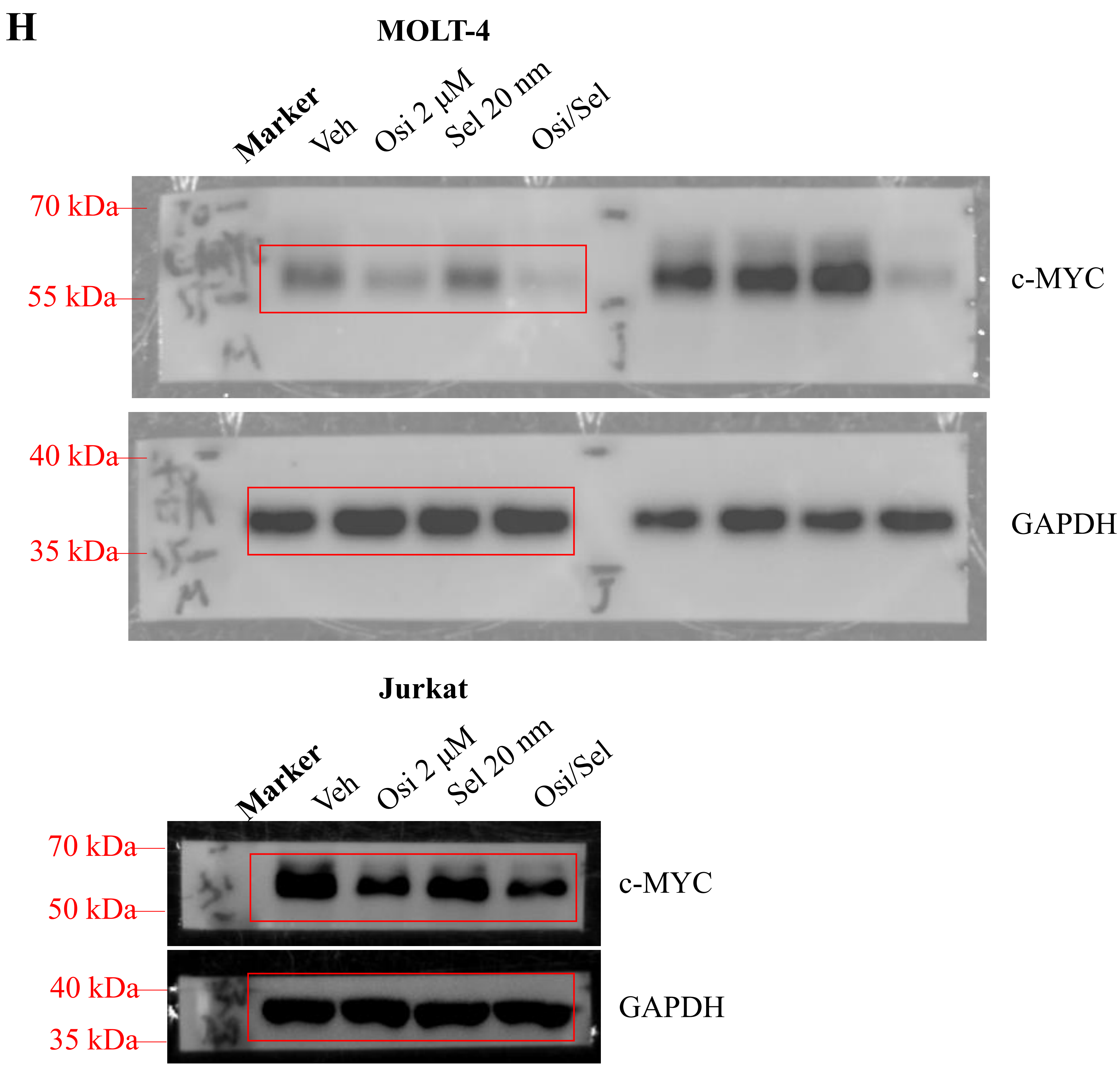

Figure 6 The selinexor-osimertinib combination induces nucleolar stress, translational suppression, and c-MYC depletion.

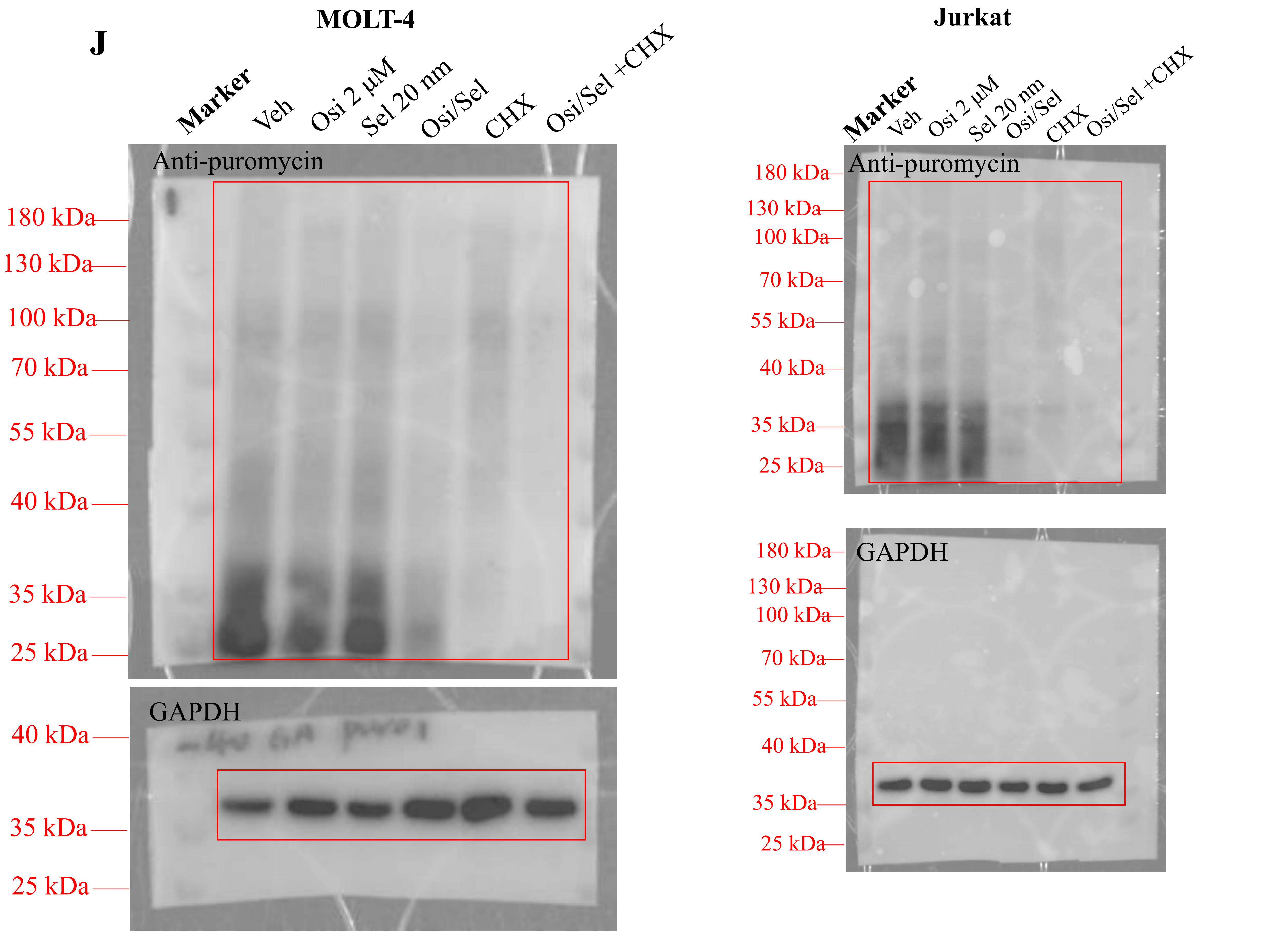

Supplementary Figure S1 Osimertinib induces caspase-dependent apoptosis, rather than necroptosis or ferroptosisin, in additional T-ALL cell lines.

C

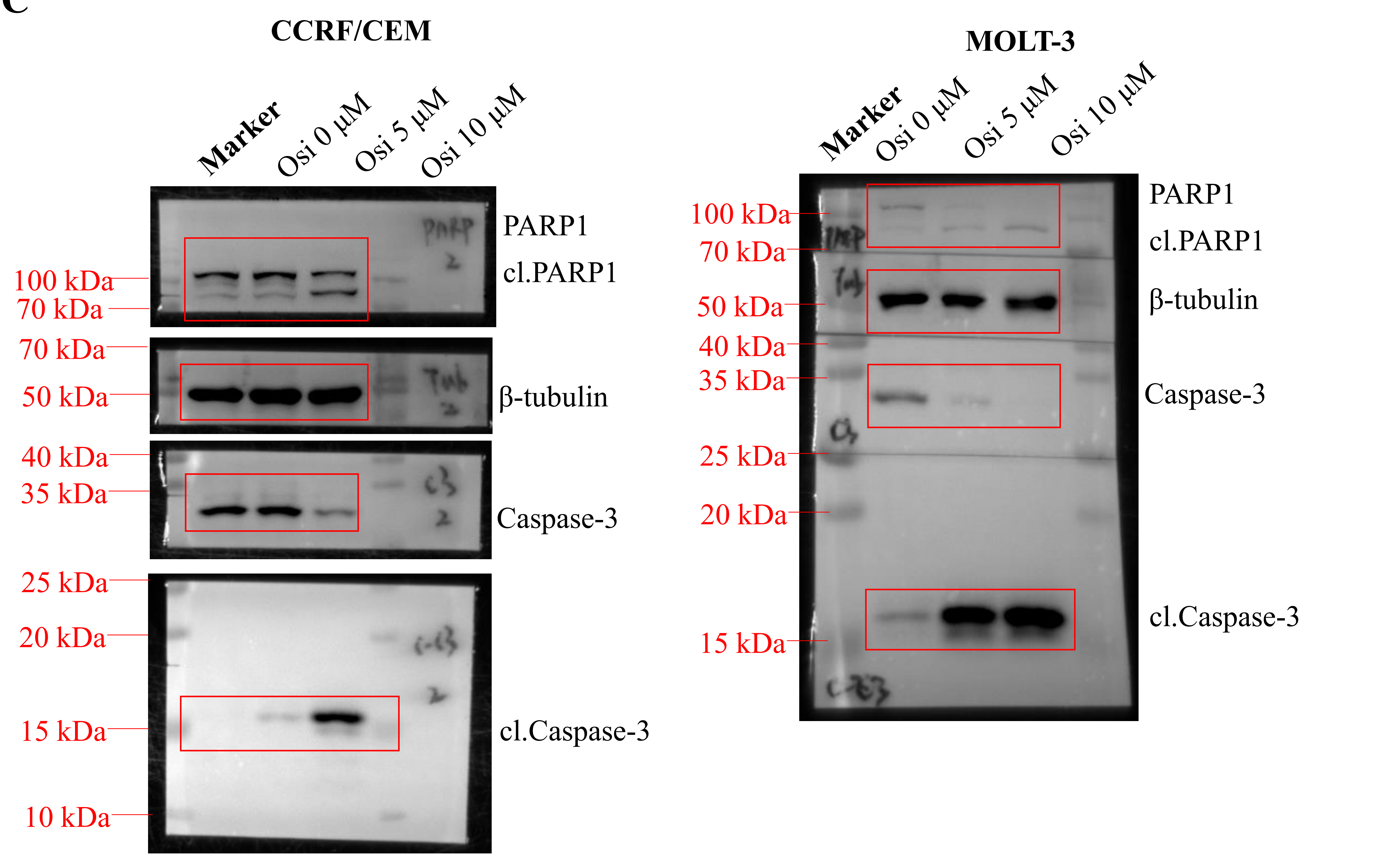

Supplementary Figure S2 Transcriptomic and biochemical validation of EGFR independence and downregulation of PI3K/AKT/mTOR and NOTCH1/c-MYC signaling.

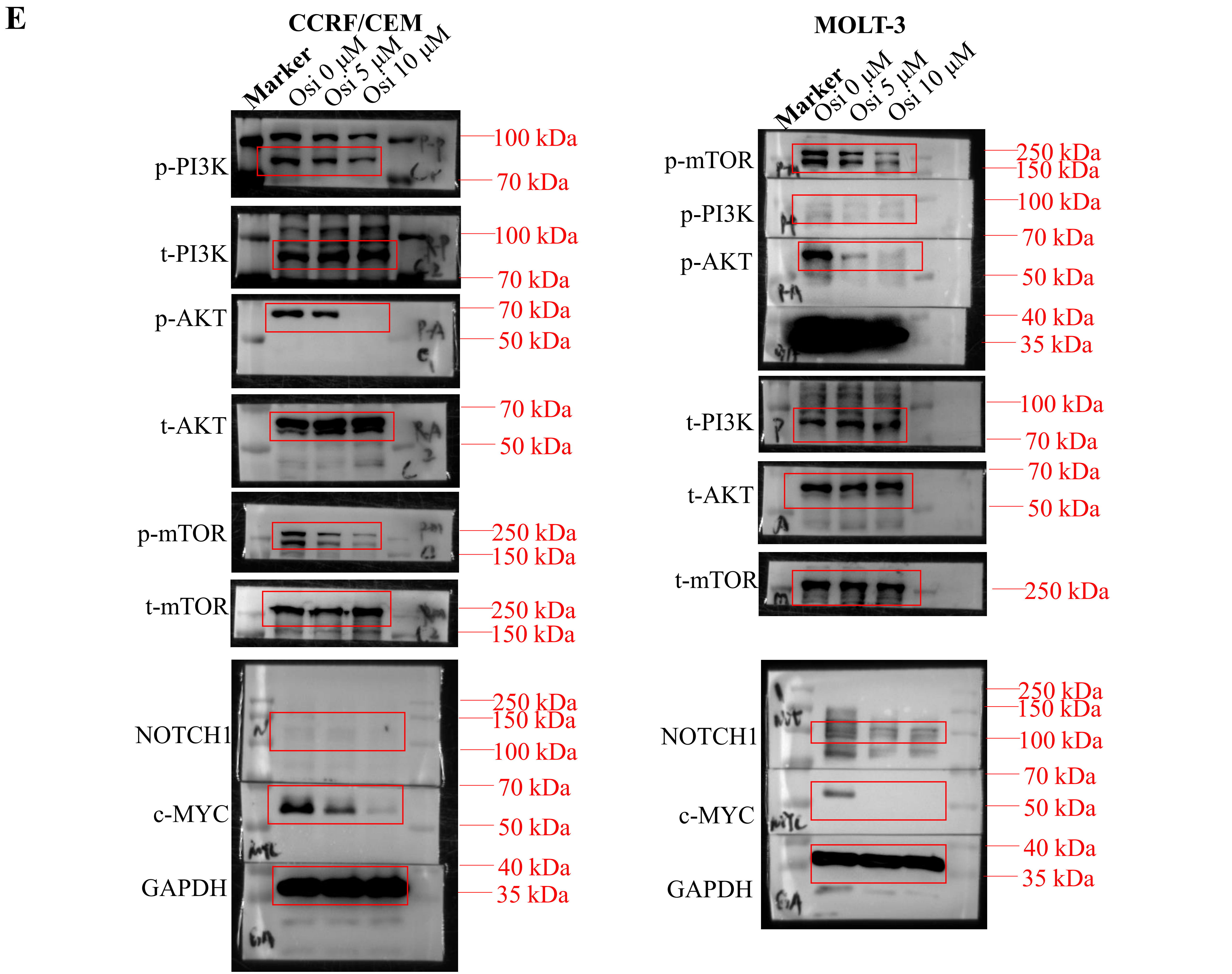

Supplementary Figure S2 Transcriptomic and biochemical validation of EGFR independence and downregulation of PI3K/AKT/mTOR and NOTCH1/c-MYC signaling.

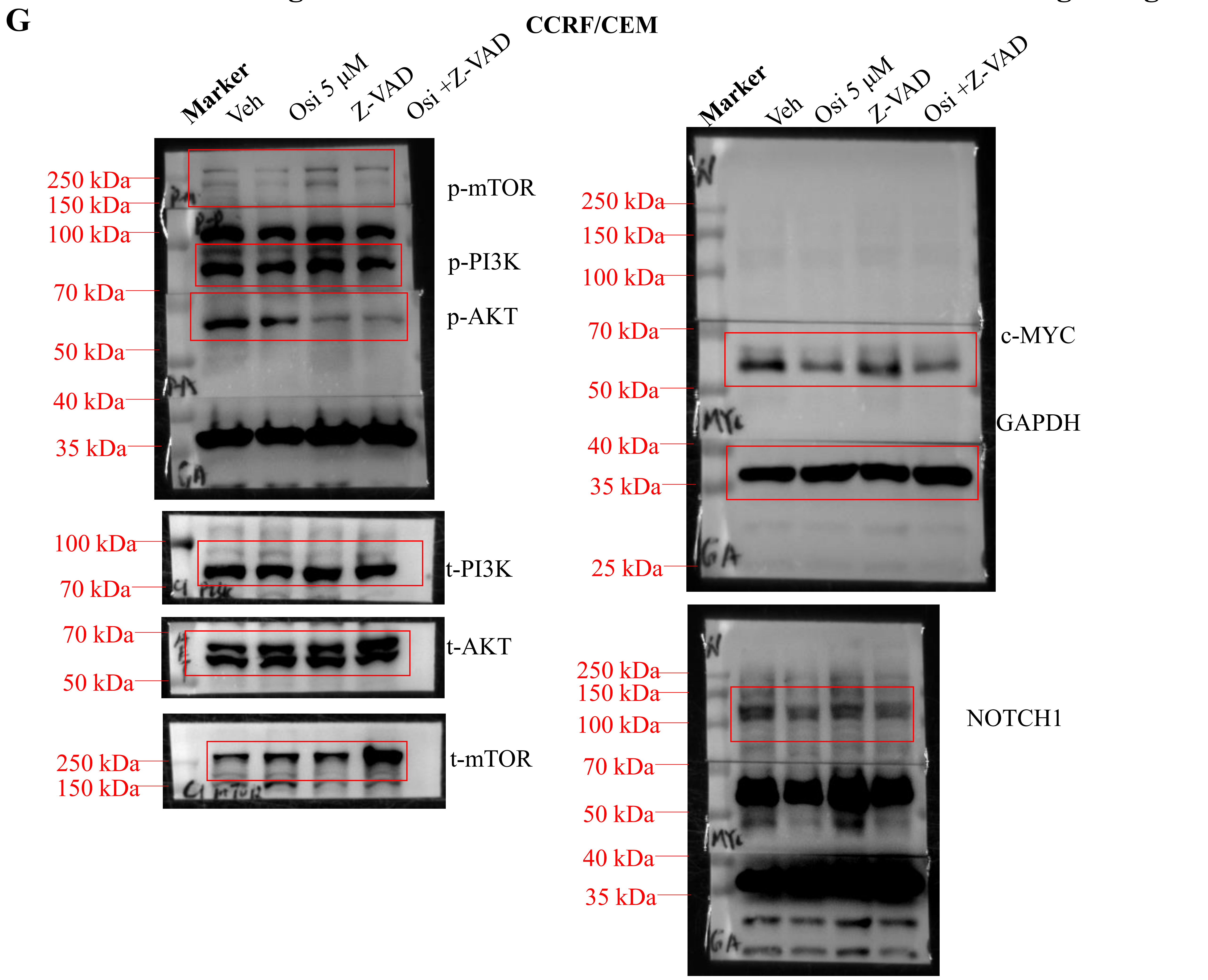

Supplementary Figure S2 Transcriptomic and biochemical validation of EGFR independence and downregulation of PI3K/AKT/mTOR and NOTCH1/c-MYC signaling.

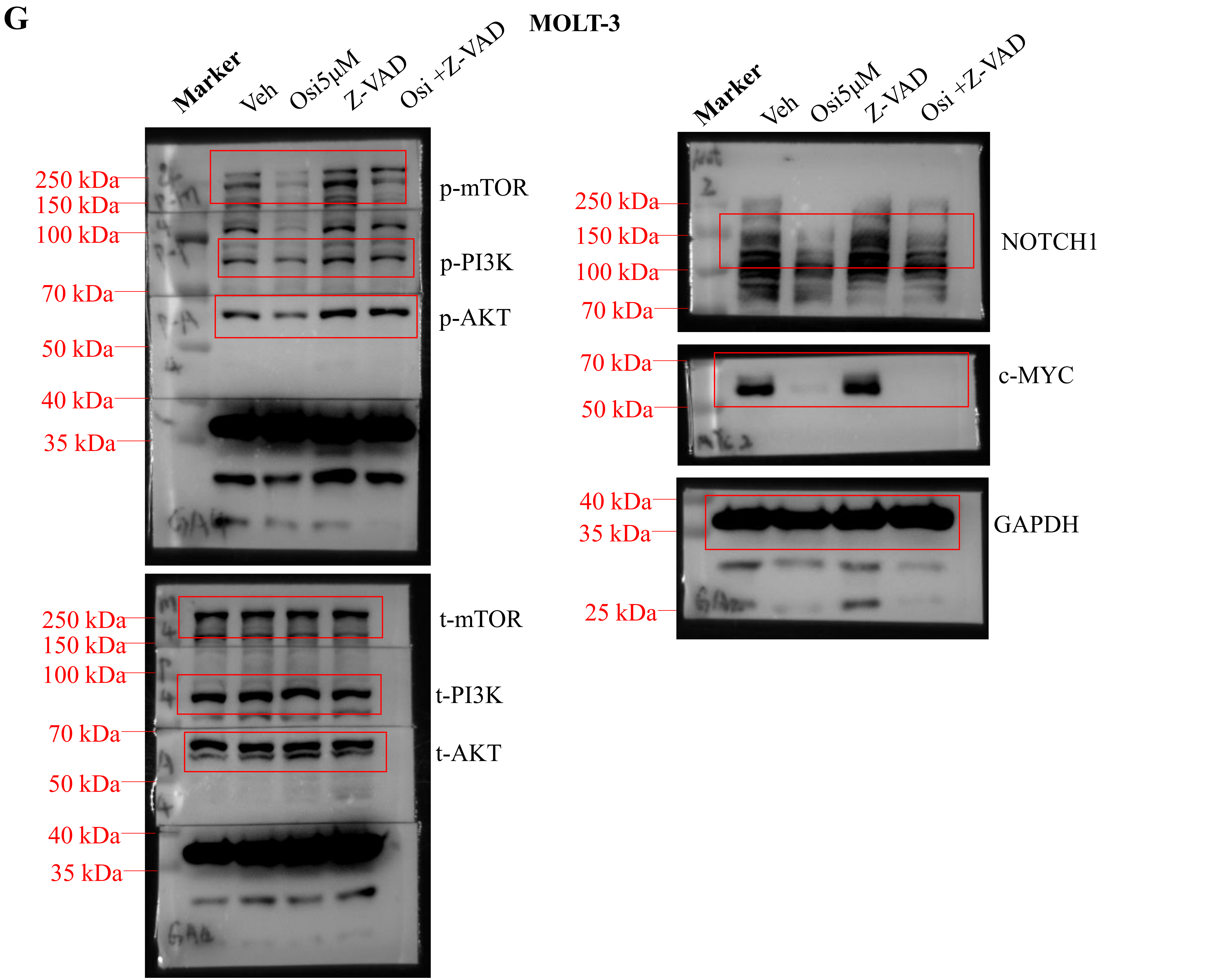

Supplementary Figure S3 Validation of pathway modulators and c-MYC stability in T-ALL cells.

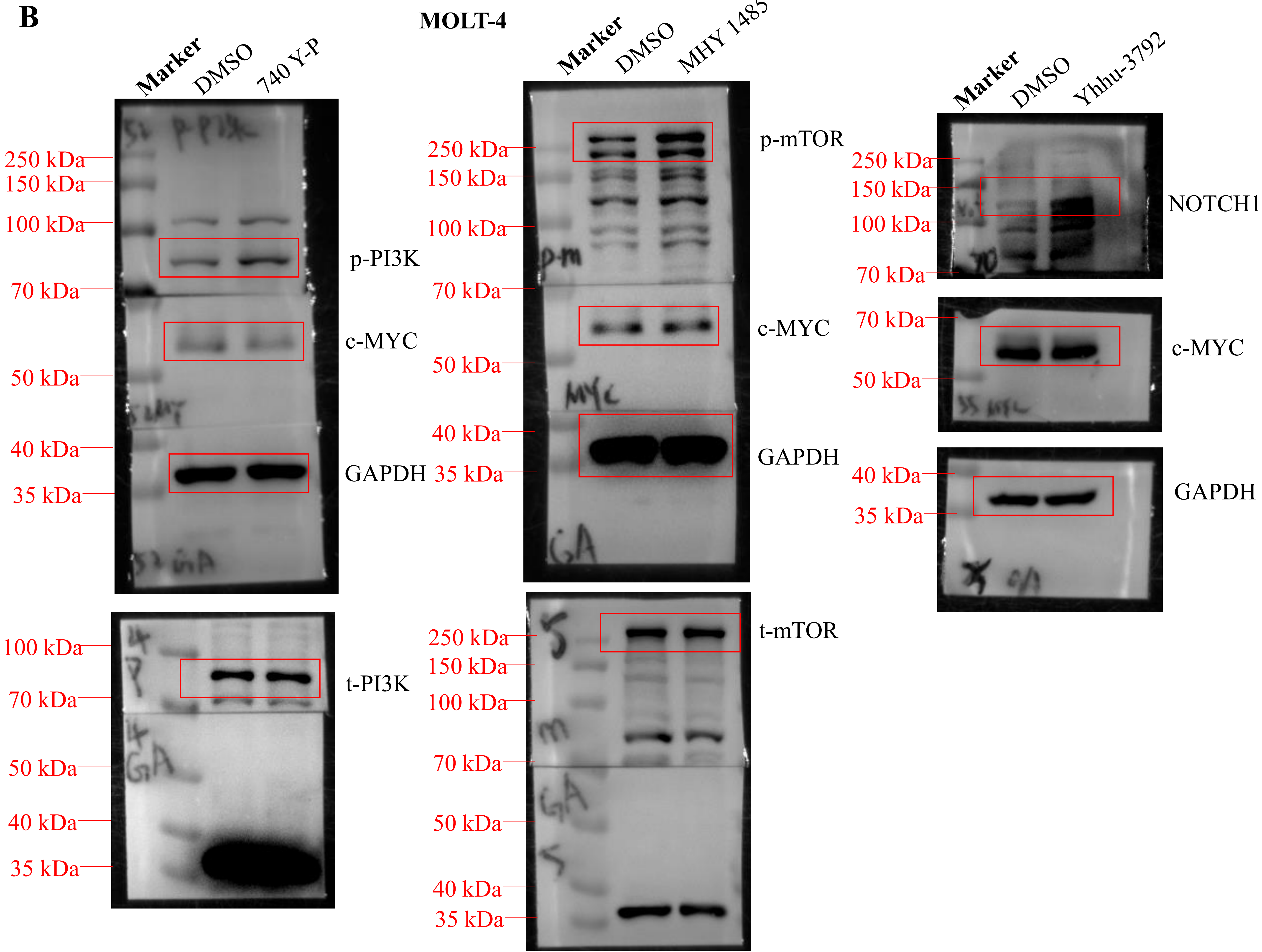

Supplementary Figure S3 Validation of pathway modulators and c-MYC stability in T-ALL cells.

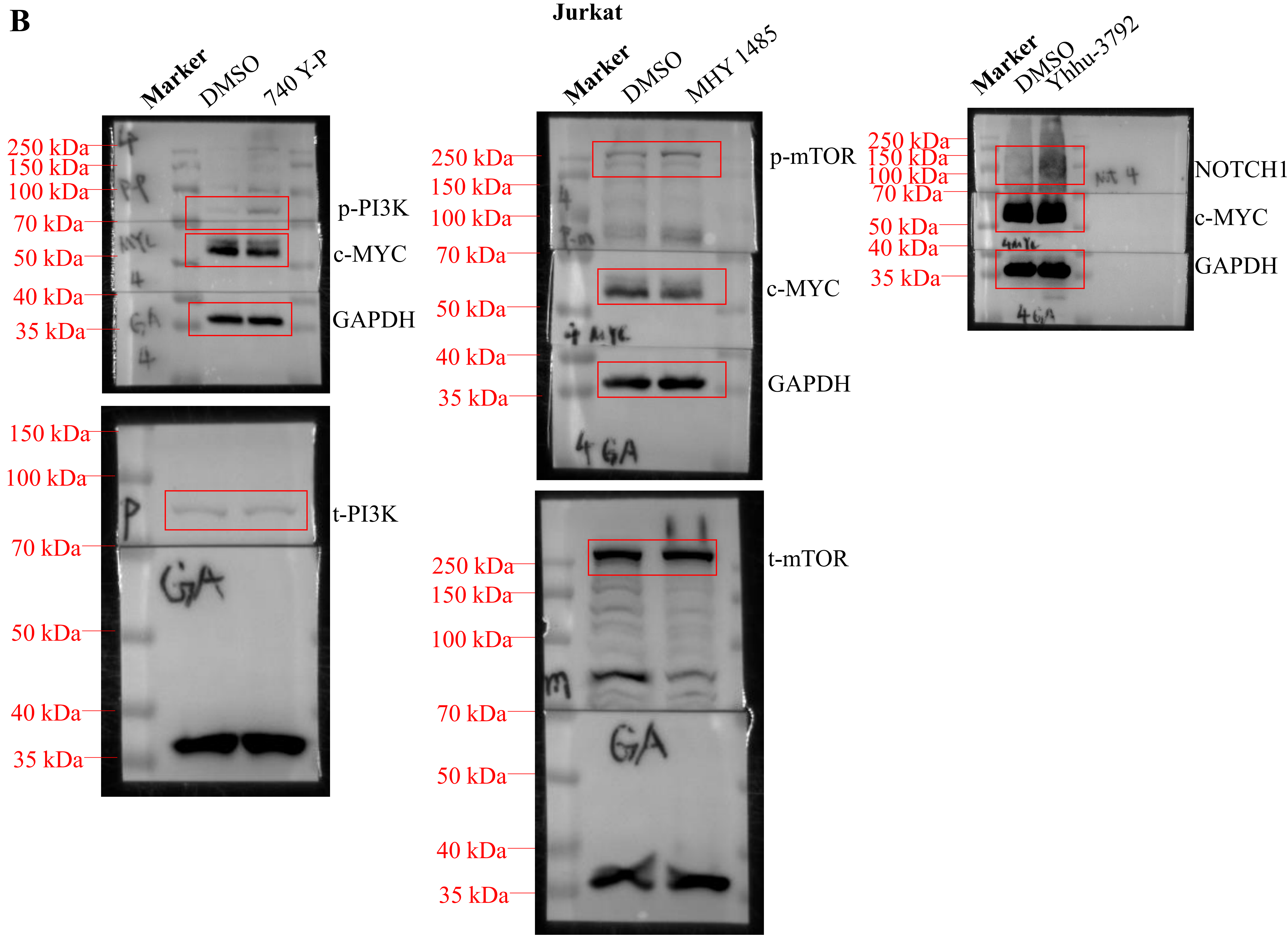

Supplementary Figure S4 Osimertinib and selinexor increase apoptosis in additional T-ALL cell lines, with clear synergy at 2 μM.

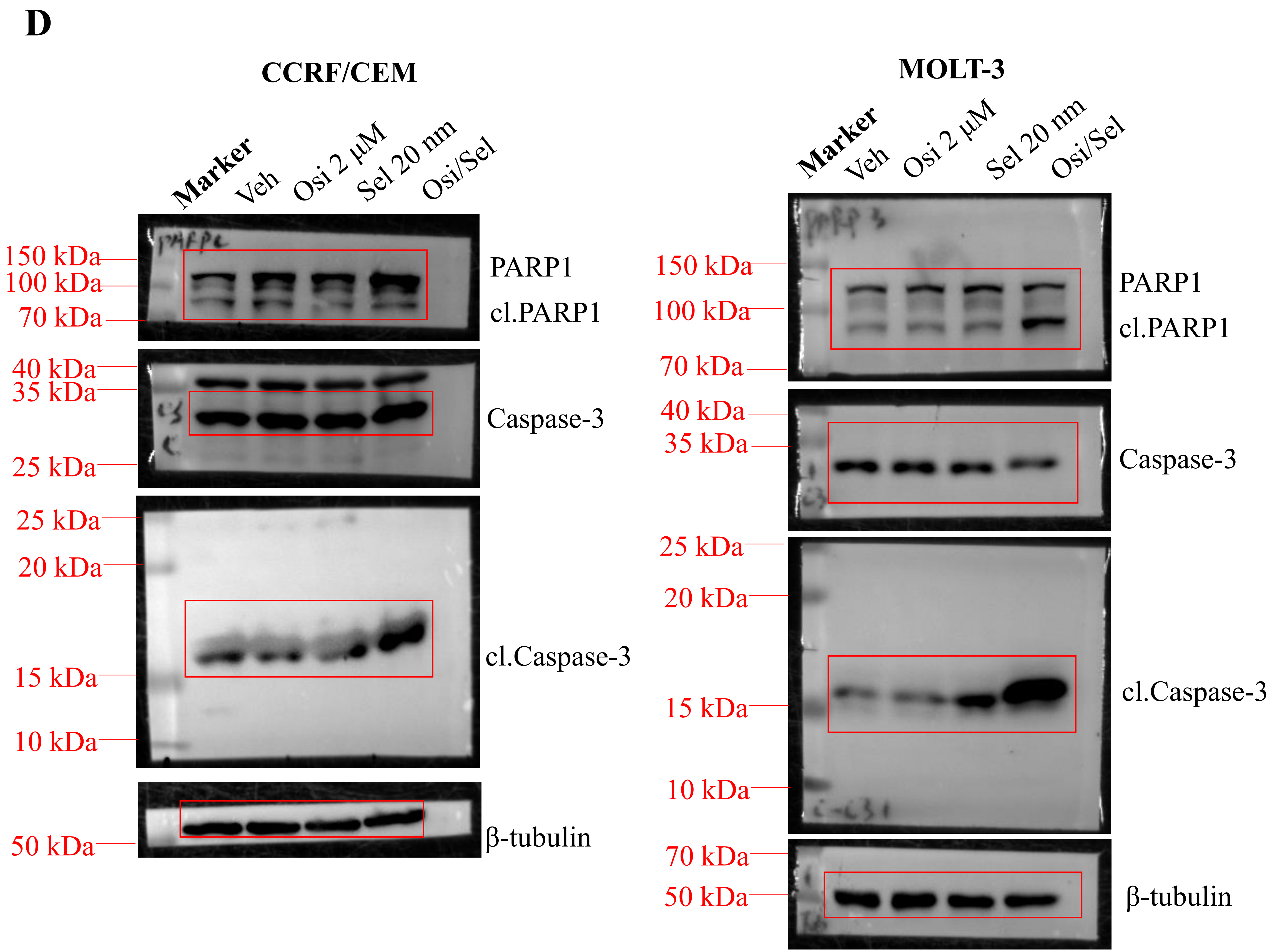

Supplementary Figure S7 Combination therapy engages MDM2/p53/BAX-associated intrinsic apoptotic signaling and reduces c-MYC in additional T-ALL cell lines.

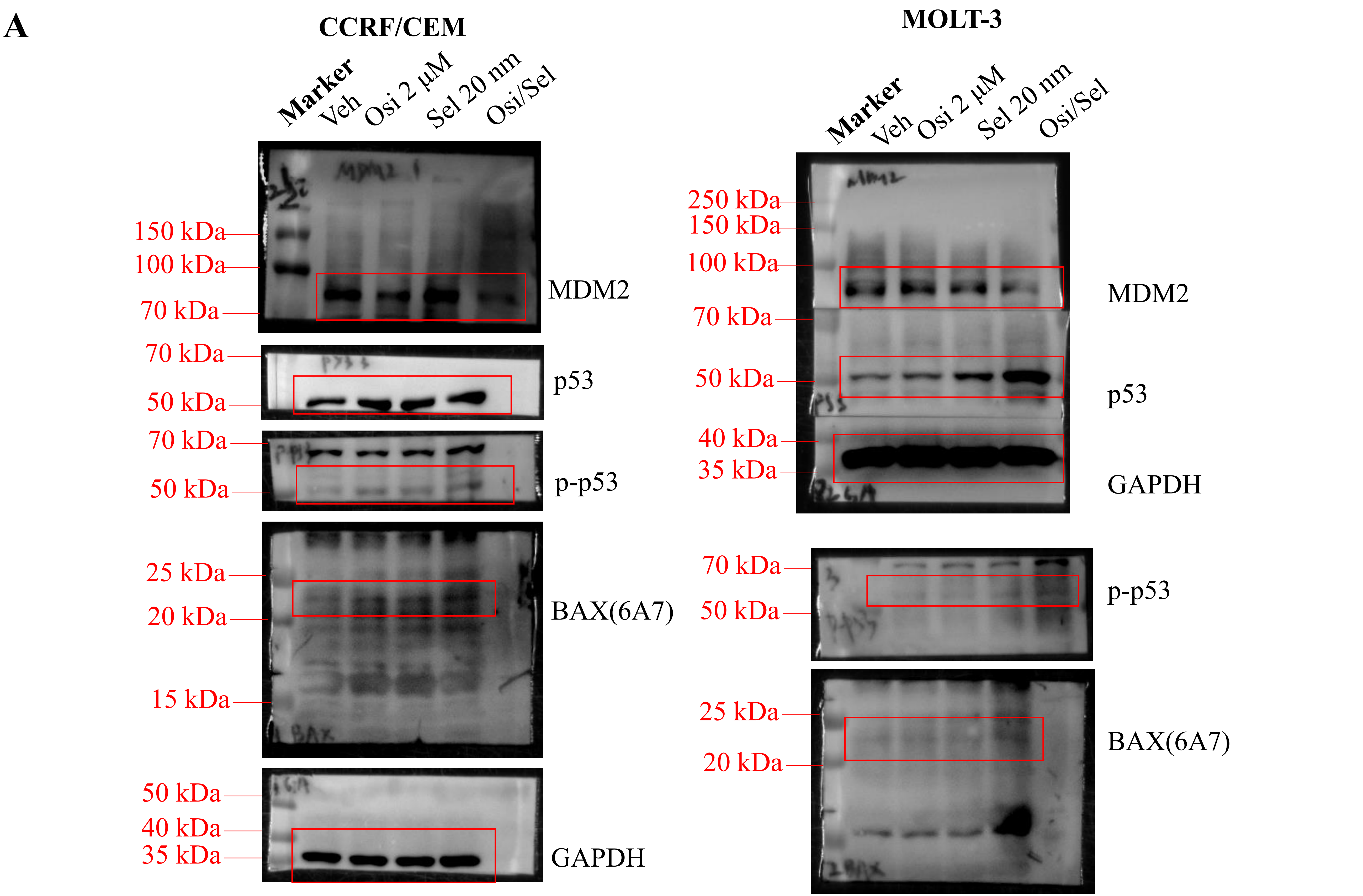

Supplementary Figure S7 Combination therapy engages MDM2/p53/BAX-associated intrinsic apoptotic signaling and reduces c-MYC in additional T-ALL cell lines.

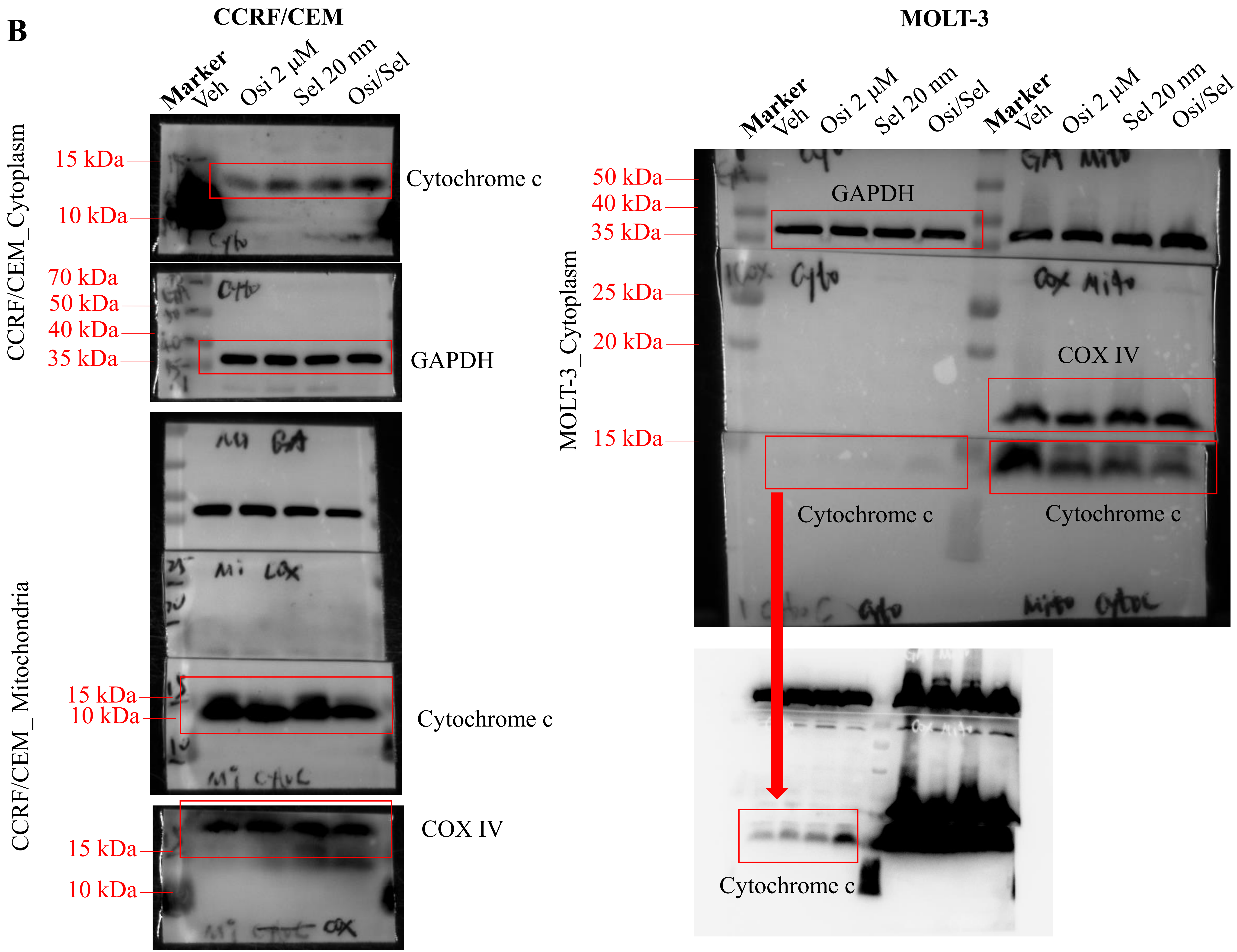

Supplementary Figure S7 Combination therapy engages MDM2/p53/BAX-associated intrinsic apoptotic signaling and reduces c-MYC in additional T-ALL cell lines.

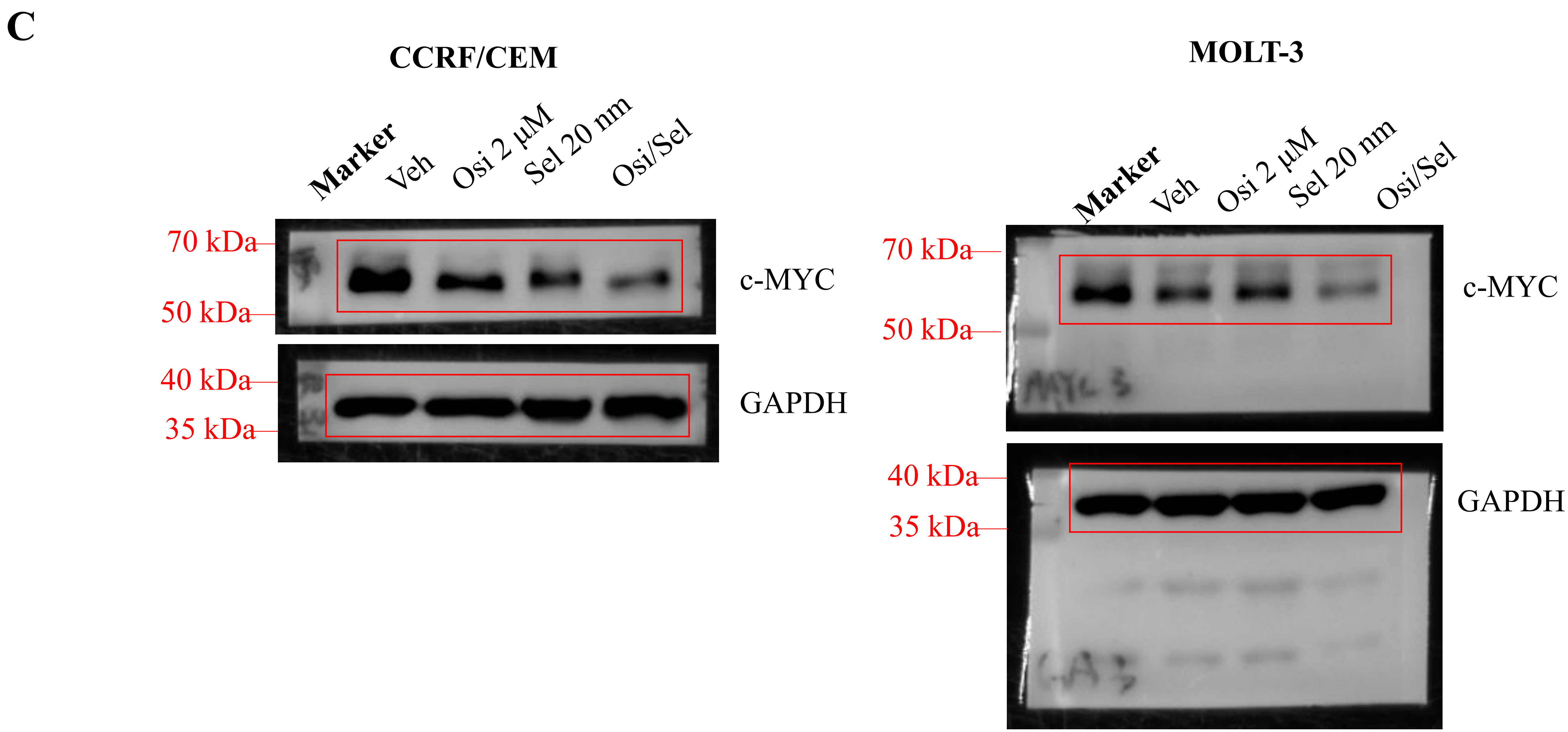

Supplement: Supplementary file 1 [file 40364_2026_1031_MOESM4_ESM.pdf]
