# Supplementary material for: Synergistic efficacy of the EGFR inhibitor osimertinib with the XPO1 inhibitor selinexor in T-cell acute lymphoblastic leukemia
Source: Biomark Res. 2026 Oct 28;14:152. doi: 10.1186/s40364-026-01031-w (PMC13718067; doi:10.1186/s40364-026-01031-w)
Supplement: Supplementary file 2 [file 40364_2026_1031_MOESM1_ESM.docx]

**Supplementary Figures**

**Supplementary Figure S1 Osimertinib induces caspase-dependent apoptosis, rather than necroptosis or ferroptosis, in additional T-ALL cell lines.**

(A) Dose-response curves and calculated IC₅₀ values of osimertinib in CCRF/CEM and MOLT-3 cells treated for 24 h, and in MOLT-4, Jurkat, CCRF/CEM, and MOLT-3 cells treated for 48 h (n = 3).

(B) Apoptosis measured by Annexin V-APC/PI staining in CCRF/CEM and MOLT-3 cells treated with the indicated concentrations of osimertinib (0, 0.5, 1, 2, 5, or 10 µM) for 24 h (n = 3).

(C) Immunoblot analysis (top) and quantification (bottom) of cleaved PARP1 and cleaved Caspase-3 in CCRF/CEM and MOLT-3 cells treated with osimertinib (0, 5, or 10 µM) for 24 h. β-tubulin, loading control (n = 3).

(D) Apoptosis in CCRF/CEM and MOLT-3 cells treated with vehicle, vehicle plus Z-VAD-FMK (20 µM), osimertinib (5 µM) alone, or osimertinib (5 µM) plus Z-VAD-FMK (20 µM). For Z-VAD-FMK-containing groups, cells were pretreated with Z-VAD-FMK for 2 h, followed by vehicle or osimertinib for an additional 24 h (n = 3).

(E) Cell proliferation in MOLT-4, Jurkat, CCRF/CEM, and MOLT-3 cells treated for 48 h with osimertinib (5 µM) in the presence or absence of necrostatin-1 (Nec-1, 5 µM) or ferrostatin-1 (Fer-1, 2 µM). Single-agent controls for Nec-1 and Fer-1 were included (n=3).

Data are presented as mean ± SD. **p*<0.05, ***p*<0.01, ****p*<0.001, *****p*<0.0001; ns, not significant.

**Supplementary Figure S2 Transcriptomic and biochemical validation of EGFR independence and downregulation of PI3K/AKT/mTOR and NOTCH1/c-MYC signaling.**

(A) Apoptosis analysis of MOLT-4 cells treated with osimertinib (5 µM) in combination with EGF (100 ng/ml) for 24 **h. Apoptotic cells were measured by Annexin V-APC/PI staining followed by flow cytometry (n = 3).**
(B) KEGG pathway enrichment analysis of downregulated genes in MOLT-4 cells after treatment with osimertinib (5 µM) for 16 **h**.
(C) GSEA analysis of down-regulated genes in MOLT-4 cells after treatment with osimertinib (5 µM) for 16 **h**.
(D) Quantification of immunoblot analysis of p-PI3K/t-PI3K, p-AKT/t-AKT, p-mTOR/t-mTOR, NOTCH1, and c-MYC in MOLT-4 and Jurkat cells treated with osimertinib (0, 5, or 10 µM) for 12 h (n = 3).
(E) Immunoblot analysis (left) and quantification (right) of p-PI3K/t-PI3K, p-AKT/t-AKT, p-mTOR/t-mTOR, NOTCH1, and c-MYC in CCRF/CEM and MOLT-3 cells treated with osimertinib (0, 5, or 10 µM) for 24 h. GAPDH, loading control (n = 3).
(F) Quantification of immunoblot analysis of p-PI3K/t-PI3K, p-AKT/t-AKT, p-mTOR/t-mTOR, NOTCH1, and c-MYC in MOLT-4 and Jurkat cells treated with vehicle, osimertinib (5 µM) alone, Z-VAD-FMK (20 µM) alone, or osimertinib in combination with Z-VAD-FMK. Where indicated, cells were pre-treated with Z-VAD-FMK for 2 h before osimertinib treatment for 24 h (n = 3).
(G) Immunoblot analysis (left) and quantification (right) of p-PI3K/t-PI3K, p-AKT/t-AKT, p-mTOR/t-mTOR, NOTCH1, and c-MYC in CCRF/CEM and MOLT-3 cells treated with vehicle, osimertinib (5 µM) alone, Z-VAD-FMK (20 µM) alone, or osimertinib in combination with Z-VAD-FMK. Where indicated, cells were pre-treated with Z-VAD-FMK for 2 h before osimertinib treatment for 24 h. GAPDH was used as a loading control (n = 3).

Data are presented as mean ± SD. **p* < 0.05, ***p* < 0.01, ****p* < 0.001, *****p* < 0.0001; ns, not significant.

**Supplementary Figure S3 Validation of pathway modulators and c-MYC stability in T-ALL cells.**

(A) Representative flow-cytometry plots of apoptosis analysis by Annexin V-APC/PI staining in MOLT-4 and Jurkat cells treated with osimertinib (5 µM) alone or in combination with 740 Y-P (30 µM), MHY1485 (10 µM), or Yhhu-3792 (2.5 µM) for 24 h (n = 3).
(B) Immunoblot analysis (left) and quantification (right) of p-PI3K/t-PI3K, p-mTOR/t-mTOR, NOTCH1, and c-MYC in MOLT-4 and Jurkat cells treated with 740 Y-P (30 µM), MHY1485 (10 µM), or Yhhu-3792 (2.5 µM) alone for 24 h. GAPDH, loading control (n = 3).
(C) Representative flow-cytometry plots of apoptosis by Annexin V-APC/PI staining in MOLT-4 and Jurkat cells treated with osimertinib (5 µM) alone or in combination with LY294002 (10 µM), rapamycin (0.1 µM), or DAPT (10 µM) for 24 h (n = 3).
(D) Quantification of p-PI3K/t-PI3K, p-mTOR/t-mTOR, NOTCH1, and c-MYC in cells treated as in (C). GAPDH, loading control (n = 3).

(E-I) All experiments shown in panels E-I were performed in MOLT-4 cells.

(E) Quantitative melting curve of c-MYC plotted as relative band intensity versus temperature (n = 3).
(F) Time-course quantification of c-MYC degradation from CETSA at room temperature (n = 3).
(G) Quantitative analysis of c-MYC stability from DARTS assay at different pronase-to-protein ratios (n = 3).
(H) Quantification of c-MYC protein decay in cycloheximide-chase assay (n = 3).
(I) Quantification of c-MYC protein levels in MOLT-4 cells treated with vehicle, osimertinib (5 µM) alone, MG132 (0.5 µM) alone, or osimertinib in combination with MG132. Where indicated, cells were pre-treated with MG132 for 2 h before osimertinib treatment for 12 h. Data are normalized to vehicle (n = 3).

Data are presented as mean ± SD. **p* < 0.05, ***p* < 0.01, ****p* < 0.001, *****p* < 0.0001; ns, not significant.

**Supplementary Figure S4 Osimertinib and selinexor increase apoptosis in additional T-ALL cell lines, with clear synergy at 2 µM.**

(A) Dose-response matrix analysis (HSA model) quantifying the synergistic effect of osimertinib and selinexor in CCRF/CEM and MOLT-3 cells. Synergy scores are indicated.

(B) Dose-response curves of osimertinib alone or combined with a fixed dose of selinexor (20 nM) in CCRF/CEM and MOLT-3 cells. Dashed lines indicate IC_50_ values.

(C) Apoptosis analysis by Annexin V-APC/PI staining in CCRF/CEM and MOLT-3 cells treated for 24 h with vehicle, osimertinib (0.5, 1, or 2 µM) alone, selinexor (20 nM) alone, or osimertinib (0.5, 1, or 2 µM) combined with selinexor (20 nM). Each concentration was analyzed separately (n = 3).

(D) Immunoblot analysis (left) and quantification (right) of cleaved PARP1 and cleaved Caspase-3 in CCRF/CEM and MOLT-3 cells treated with vehicle, osimertinib (2 µM) alone, selinexor (20 nM) alone, or osimertinib (2 µM) combined with selinexor (20 nM). β-tubulin, loading control (n = 3).

(E) Apoptosis analysis by Annexin V-APC/PI staining in MOLT-4 and Jurkat cells treated for 24 h with vehicle, osimertinib (0.5 or 1 µM) alone, selinexor (20 nM) alone, or osimertinib (0.5 or 1 µM) combined with selinexor (20 nM). Each concentration was analyzed separately (n = 3).

(F) Apoptosis in CCRF/CEM and MOLT-3 cells treated with vehicle, vehicle plus Z-VAD-FMK (20 µM), the combination (osimertinib 2 µM + selinexor 20 nM), or the combination plus Z-VAD-FMK (20 µM). For Z-VAD-FMK-containing groups, cells were pretreated with Z-VAD-FMK (20 µM) for 2 h, followed by vehicle or the combination for an additional 24 h (n = 3).

Data are presented as mean ± SD. **p*<0.05, ***p*<0.01, ****p*<0.001, *****p*<0.0001; ns, not significant.

**Supplementary Figure S5 Cytotoxicity of the osimertinib-selinexor combination in normal human BMMNCs and HEK293T cells.**

(A) Dose-response analysis by CCK-8 assay in normal human bone marrow mononuclear cells (BMMNCs) treated with vehicle, osimertinib (2, 5, or 10 µM) alone, selinexor (20, 50, or 100 nM) alone, or the corresponding combination for 24 h and 48 h. BMMNCs from two independent healthy donors were analyzed; each condition was assayed in triplicate. Low, 2 µM osimertinib + 20 nM selinexor; medium, 5 µM + 50 nM; high, 10 µM + 100 nM.
(B) Dose-response analysis by CCK-8 assay in HEK293T cells treated with vehicle, osimertinib (2, 5, or 10 µM) alone, selinexor (20, 50, or 100 nM) alone, or the corresponding combination for 24 h and 48 h (n = 3). Low, 2 µM osimertinib + 20 nM selinexor; medium, 5 µM + 50 nM; high, 10 µM + 100 nM.

Data are normalized to vehicle and presented as mean ± SD. **p* < 0.05, ***p* < 0.01, ****p* < 0.001, *****p* < 0.0001; ns, not significant.

**Supplementary Figure S6 Quantitative analysis of nucleolar stress and downstream signaling in osimertinib-selinexor-treated T-ALL cells.**

MOLT-4 and Jurkat cells were treated with vehicle, osimertinib (2 µM), selinexor (20 nM), or the combination for 12 h (A-F). For (G), MOLT-4 cells were treated for 12 h and Jurkat cells for 24 h. For (H), MOLT-4 cells were treated for 24 h and Jurkat cells for 12 h. All experiments were performed with n = 3.

(A) Quantification of nuclear/cytoplasmic RPS6 ratios. At least 30 cells per group were analyzed.
(B) Quantification of total nuclear FBL fluorescence intensity.
(C) Quantification of nuclear/cytoplasmic RPL5 ratios. At least 30 cells per group were analyzed.
(D) Quantification of nuclear/cytoplasmic atios of RPL5 and RPL11 determined by biochemical fractionation.
(E) Quantification of MDM2, p53, phosphorylated p53 (p-p53) and activated BAX (6A7) protein levels.
(F) Quantification of cytoplasmic and mitochondrial cytochrome c protein levels.
(G) Quantification of c-MYC protein levels.
(H) Quantification of global protein synthesis assessed by puromycin incorporation. Cells were treated with vehicle, osimertinib (2 µM), selinexor (20 nM), the combination, CHX alone, or the combination plus CHX. Puromycin was added to all groups 30 min before sample collection. CHX (10 µg/mL) was added 1 h before sample collection. CHX-containing groups served as controls for translation inhibition.

Data are presented as mean ± SD. **p*<0.05, ***p*<0.01, ****p*<0.001, *****p*<0.0001; ns, not significant.

**Supplementary Figure S7** **Combination therapy engages MDM2/p53/BAX-associated intrinsic apoptotic signaling and reduces c-MYC in additional T-ALL cell lines.**

(A) Immunoblot analysis (left) and quantification (right) of MDM2, p53, phosphorylated p53 (p-p53), and activated BAX (6A7) in CCRF/CEM and MOLT-3 cells treated with vehicle, osimertinib (2 µM), selinexor (20 nM), or the combination for 24 h. GAPDH, loading control (n = 3).
(B) Immunoblot analysis (left) and quantification (right) of cytochrome c levels in the cytoplasmic and mitochondrial fractions of CCRF/CEM and MOLT-3 cells treated as in (A). GAPDH and COX IV were used as cytoplasmic and mitochondrial loading controls, respectively (n = 3).
(C) Immunoblot analysis and quantification of c-MYC protein levels in CCRF/CEM cells (left) and MOLT-3 cells (right) treated as in (A) (n = 3).

Data are presented as mean ± SD. **p*<0.05, ***p*<0.01, ****p*<0.001, *****p*<0.0001; ns, not significant.

**Supplementary Table 1: Antibodies used for immunoblotting, Flow cytometry immunofluorescence and immunohistochemistry.**

| Application | Target | Manufacturer | Catalog No. | Dilution |
| --- | --- | --- | --- | --- |
| Immunoblotting | BAX（6A7） | Beyotime | AB026 | 1:1000 |
|  | β-tubulin | Proteintech | 10094-1-AP | 1:2000 |
|  | Caspase-3 | Cell Signaling Technology | #9662 | 1:1000 |
|  | Cleaved Caspase-3 | Cell Signaling Technology | #9664 | 1:1000 |
|  | c-MYC | Cell Signaling Technology | #9402 | 1:1000 |
|  | COX Ⅳ | Proteintech | 11242-1-AP | 1:5000 |
|  | Cytochrome c | Proteintech | 66264-1-Ig | 1:5000 |
|  | EGFR | Proteintech | 66455-1-Ig | 1:5000 |
|  | GAPDH | Proteintech | 10494-1-AP | 1:5000 |
|  | Lamin B | Proteintech | 12987-1-AP | 1:5000 |
|  | MDM2 | ABclonal | A0345 | 1:1000 |
|  | NOTCH1 | Proteintech | 20687-1-AP | 1:1000 |
|  | PARP1 | Cell Signaling Technology | #9542 | 1:1000 |
|  | p-AKT | ABclonal | AP0637 | 1:1000 |
|  | p-mTOR | ABclonal | AP0115 | 1:1000 |
|  | p-PI3K | ABclonal | AP0427 | 1:1000 |
|  | p-p53 | Proteintech | 80195-1-RR | 1:2000 |
|  | p53 | Proteintech | 21891-1-AP | 1:5000 |
|  | Puromycin | ABclonal | A23031 | 1:2000 |
|  | RPL5 | Proteintech | 29092-1-AP | 1:1000 |
|  | RPL11 | Proteintech | 16277-1-AP | 1:1000 |
|  | t-AKT | ABclonal | A22412 | 1:1000 |
|  | t-mTOR | ABclonal | A2445 | 1:1000 |
|  | t-PI3K | ABclonal | A18355 | 1:1000 |
| Flow cytometry | hCD45 | BioLegend | 304005 | 1:100 |
| Immunofluorescence | FBL | Proteintech | 16021-1-AP | 1:200 |
|  | RPS6 | Proteintech | 66886-1-IG | 1:200 |
|  | RPL5 | Proteintech | 29092-1-AP | 1:200 |
| Immunohistochemistry | Cleaved Caspase-3 | Cell Signaling Technology | #9664 | 1:100 |
|  | c-MYC | Cell Signaling Technology | #9402 | 1:50 |
|  | Ki-67 | Cell Signaling Technology | #9129 | 1:400 |

Abbreviations: FBL=Fibrillarin.

**Supplementary Table 2: Primers used for quantitative real-time PCR**

| Primer Name | Primer Sequence (5'→3') |
| --- | --- |
| h*c-MYC*-F | CCTGGTGCTCCATGAGGAGAC |
| h*c-MYC*-R | CAGACTCTGACCTTTTGCCAGG |
| h*GAPDH*-F | GGAGCGAGATCCCTCCAAAAT |
| h*GAPDH*-R | GGCTGTTGTCATACTTCTCATGG |
